# Supplementary material for: Synthesis of extended polycyclic aromatic hydrocarbons by oxidative tandem spirocyclization and 1,2-aryl migration
Source: Nat Commun. 2017 Apr 25;8:15073. doi: 10.1038/ncomms15073 (PMC5414065; doi:10.1038/ncomms15073)
Supplement: Supplementary Information — Supplementary figures, supplementary methods and supplementary references. [file ncomms15073-s1.pdf]

Supplementary Methods

General information. ^{1}H NMR and ^{13}C NMR spectra were recorded on JEOL JNM AL 400 (400 MHz) and JEOL JNM-ECA 700 (700 MHz) spectrometers. ^{1}H NMR spectra are reported as follows: chemical shift in ppm (δ) relative to the chemical shift of $CDCl_3$ at 7.26 ppm, CD_2Cl_2 at 5.32 ppm, $C_2D_2Cl_4$ at 6.00 ppm, integration, multiplicities (s = singlet, d = doublet, t = triplet, q = quartet, m = multiplet, and br = broadened), and coupling constants (Hz). ^{13}C NMR spectra were recorded on JEOL JNM AL 400 (100.5 MHz) and JEOL JNM-ECA 700 (176.0 MHz) spectrometers with complete proton decoupling, and chemical shift reported in ppm (δ) relative to the central line for $CDCl_3$ at 77 ppm, CD_2Cl_2 at 53.8 ppm, $C_2D_2Cl_4$ at 73.79 ppm. Accurate mass data were obtained by high resolution mass spectrometry performed on a Bruker Daltonics solariX 9.4T FT-ICR-MS spectrometer or a Bruker Daltonics microflex instrument using Matrix Assisted Laser Desorption Ionization (MALDI) at the Research and Analytical Center for Giant Molecules, Graduate School of Science, Tohoku University. Column chromatography was carried out employing silica gel 60 N (spherical, neutral, 40~100 µm, KANTO Chemical Co.) and Silica gel 60 (Merck). Analytical thin-layer chromatography (TLC) was performed on 0.2 mm precoated plate Kieselgel 60 F254 (Merck).

Materials. All chemicals were purchased and used as received. The structures of products **2a**,[1] **2b**,[2] **2k**,[3] **2l**,[4] **2n**[2] were determined by ^{1}H and ^{13}C NMR spectroscopy and high-resolution mass compared with the reported references. Other starting substrates and products were determined by ^{1}H and ^{13}C NMR spectroscopy and high-resolution mass spectrometry. The structure of **2q** was determined by various 1D and 2D NMR spectroscopy.

General procedure for synthesis of 2a via CuCl-catalyzed tandem reaction of 9-(biphenyl-2-ylmethylene)-9*H*-fluorene 1a (condition A)

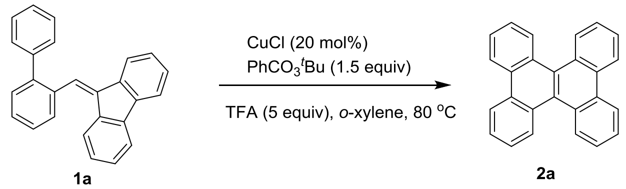

To a *o*-xylene (1.2 mL) solution of CuCl (4 mg, 20 mol %), $PhCO_3{}^tBu$ (58.3 mg, 0.3 mmol), and trifluoroacetic acid (114 mg, 1 mmol) was added 9-(biphenyl-2-ylmethylene)-9*H*-fluorene (**1a**) (66 mg, 0.2 mmol) at room temperature. The mixture was heated at 80 °C for 1.5 h. After cooling to room temperature, the reaction mixture was monitored by TLC and GC-MS. The reaction mixture was washed with water and extracted with CH_2Cl_2 for 2 times. After concentration of the CH_2Cl_2 solution, the resulting residue was purified by flash silica gel chromatography using a mixture of CH_2Cl_2/hexane as eluent to give the corresponding product **2a** in 94% yield (61.8 mg) as a colourless solid.

General procedure for synthesis of 2a via DDQ-mediated tandem reaction of 9-(biphenyl-2-ylmethylene)-9*H*-fluorene 1a (condition B)

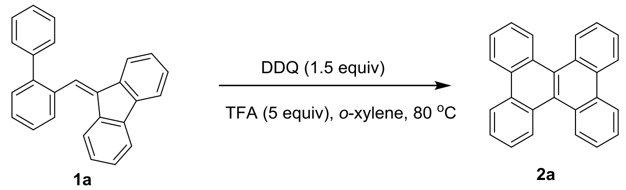

To a *o*-xylene (1.2 mL) solution of DDQ (68.1 mg, 0.3 mmol), and trifluoroacetic acid (114 mg, 1 mmol) was added 9-(biphenyl-2-ylmethylene)-9*H*-fluorene (**1a**) (66 mg, 0.2 mmol) at room temperature. The mixture was heated at 80 °C for 12 h. After cooling to room temperature, the reaction mixture was monitored by TLC and GC-MS. The reaction mixture was washed with water and extracted with CH_2Cl_2 for 2 times. After concentration of the CH_2Cl_2 solution, the resulting residue was purified by flash silica gel chromatography using a mixture of CH_2Cl_2/hexane as eluent to give the corresponding product **2a** in 87% yield (57.2 mg) as a colourless solid.

General procedure for synthesis of 1

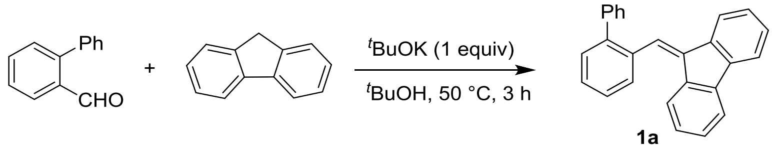

To a suspension of 9*H*-fluorene (498.6 mg, 3 mmol, purchased from TCI) in ^{t}BuOH (15 mL) was added ^{t}BuOK (336.6 mg, 3 mmol), which was stirred at 40 °C for 30 min. Then, [1,1'-biphenyl]-2-carbaldehyde (546.6 mg, 3 mmol, purchased from Aldrich) was added and the reaction mixture was allowed to warm to 50 °C and stirred for 3 h. After monitoring with TLC, the reaction mixture was extracted with dichloromethane and the organic layer was dried with Na_2SO_4. After filtration and evaporation, the residue was purified through silica gel chromatography using hexane/CH_2Cl_2 (10/1) as eluents to give the corresponding product **1a** in 93% isolated yield as a light yellow solid (921.9 mg).

Synthesis of the corresponding aldehyde precursor of 1k

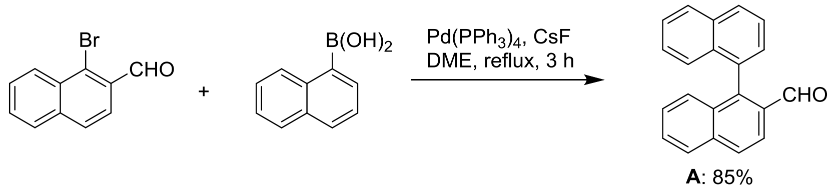

The aldehyde precursor of **1k**, [1,1'-binaphthalene]-2-carbaldehyde (**A**) was prepared through a Pd-catalyzed Suzuki-Miyaura coupling of the commercially available 1-bromo-2-naphthaldehyde (TCI) and naphthalen-1-ylboronic acid (WAKO). The structure **A** was determined by comparison with the reported authentic sample.[5]

Synthesis of the corresponding aldehyde precursor of 1l

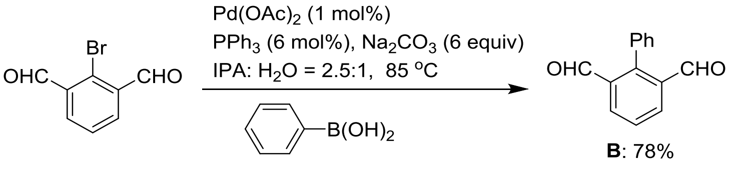

The aldehyde precursor of **1l**, biphenyl-2,6-dicarbaldehyde (**B**) was prepared through a Pd-catalyzed Suzuki-Miyaura coupling of the commercially available 2-bromoisophthalaldehyde (TCI) and phenylboronic acid (WAKO).[6] The structure **B** was determined by comparison with the reported authentic sample.[7]

Synthesis of the corresponding aldehyde precursor of 1m and 1n

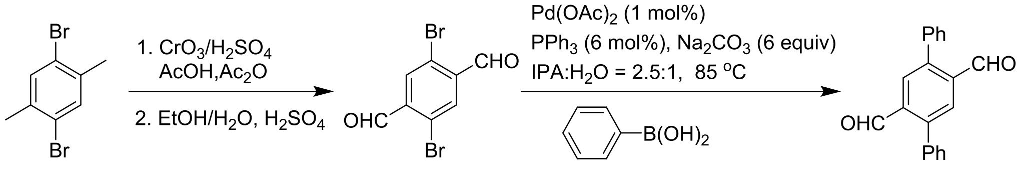

C: 48% **D**: 90%

The aldehyde precursor of **1m** and **1n**, [1,1':4',1''-terphenyl]-2',5'-dicarbaldehyde (**D**) was prepared through a Pd-catalyzed Suzuki-Miyaura coupling of the commercially available phenylboronic acid (WAKO) and 2,5-dibromoterephthalaldehyde (**C**).[6] The compound **C** was prepared by the CrO_3/H_2SO_4-mediated oxidation of the commercially available 1,4-dibromo-2,5-dimethylbenzene (TCI) following the reported method.[8] The structures **C** and **D** were determined by comparison with the reported authentic samples.[8]

Synthesis of the corresponding aldehyde precursor of 1o

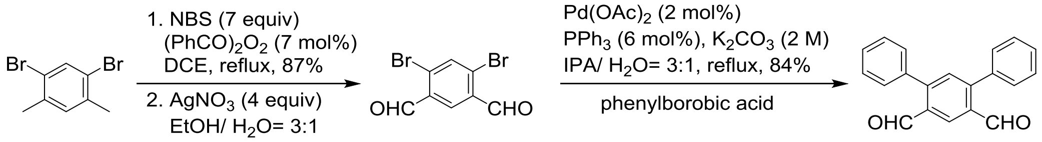

E **F**

The aldehyde precursor of **1o**, [1,1':3',1''-terphenyl]-4',6'-dicarbaldehyde (**F**) was prepared through a Pd-catalyzed Suzuki-Miyaura coupling of the commercially available phenylboronic acid (WAKO) and 4,6-dibromoisophthalaldehyde (**E**). The compound **E** was prepared by the methyl dibromination of the commercially available 1,5-dibromo-2,4-dimethylbenzene (TCI) followed by $AgNO_3$-mediated formylation following the reported method.[9]

Synthesis of the corresponding aldehyde precursor of 1p

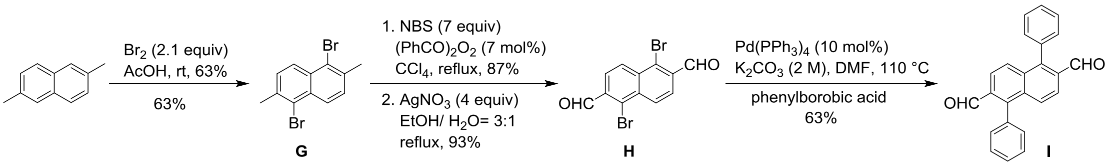

G **H** **I**

The aldehyde precursor of **1p**, 1,5-diphenylnaphthalene-2,6-dicarbaldehyde (**I**) was prepared through a Pd-catalyzed Suzuki-Miyaura coupling of the commercially available phenylboronic acid (WAKO) and 1,5-dibromonaphthalene-2,6-dicarbaldehyde (**H**). The compound **H** was prepared by the methyl dibromination and $AgNO_3$-mediated formylation of 1,5-dibromo-2,6-dimethylnaphthalene (**G**), which was prepared by the bromination of the commercially available 2,6-dimethylnaphthalene (TCI) following the reported method.[10]

Synthesis of the corresponding aldehyde precursor of 1q

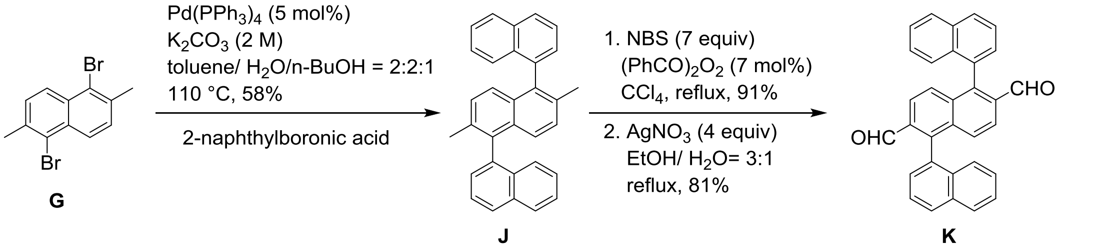

The aldehyde precursor of **1q**, [1,1':5',1''-ternaphthalene]-2',6'-dicarbaldehyde (**K**) was prepared by the methyl dibromination and $AgNO_3$-mediated formylation of 2',6'-dimethyl-1,1':5',1''-ternaphthalene (**J**),[10] which was prepared through a Pd-catalyzed Suzuki-Miyaura coupling of the compound (**G**).

DFT calculation

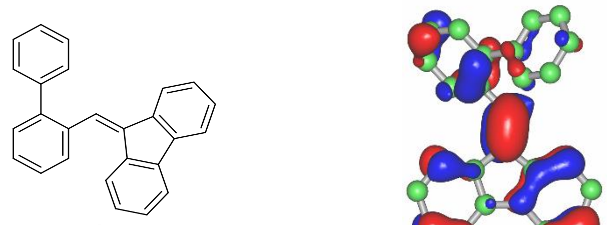

Supplementary Figure 1. DFT calculation of the HOMO of **1a**. The ground-state geometries and their molecular orbitals were calculated using the hybrid DFT energy functional B3LYP/6-31G.

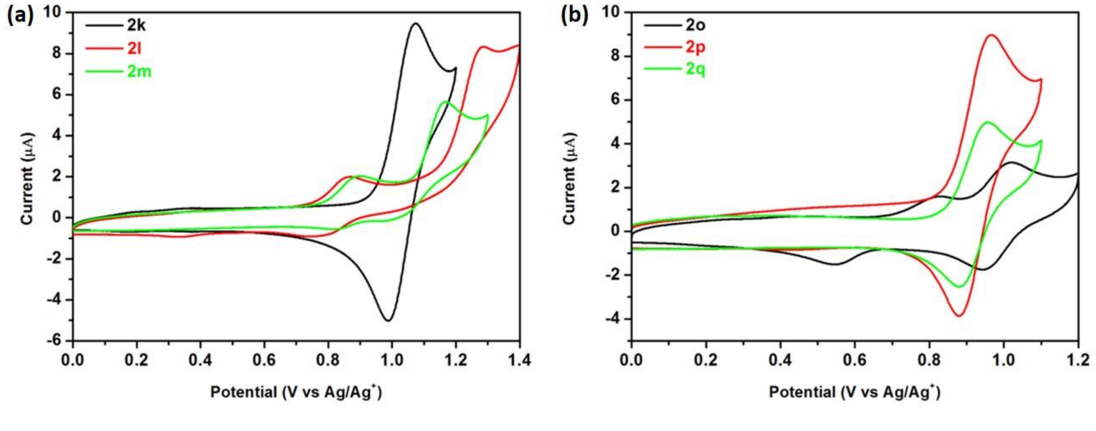

Supplementary Figure 2. Cyclic voltammograms (CV) of (a) **2k**-**m** and (b) **2o**-**q**. Potential values are versus Ag/AgCl reference electrode; 0.1 M $TBAPF_6$ as a supporting electrolyte in dichloromethane; scan rate is 50 mV s^{-1}, and the energy level of Fc/Fc^+ as -4.80 eV. The oxidation potentials ($E_{1/2}^{ox}$ *vs* Fc/Fc^+) are 0.85 eV for **2k**, 0.62 eV for **2l**, 0.68 eV and 0.88 eV for **2m**, 0.45 eV and 0.75 eV for **2o**, 0.67 eV for **2p**, and 0.70 eV for **2q**. The HOMO energy levels were calculated from the oxidation potentials ($E_{1/2}^{ox}$) according to the equation, HOMO = $-(4.80 + E_{1/2}^{ox})$ (eV).

Intermolecular competing reaction between 1i and 1i-d_5 in the same reaction vessel

To a *o*-xylene (1.2 mL) solution of CuCl (4 mg, 20 mol %), $PhCO_3{}^tBu$ (58.3 mg, 0.3 mmol), and trifluoroacetic acid (114 mg, 1 mmol) [**condition A**] or DDQ (68.1 mg, 0.3 mmol), and trifluoroacetic acid (114 mg, 1 mmol) [**condition B**] were added the protonated **1i** (47.2 mg, 0.1 mmol) and the perdeuterated **1i-d_5** (47.7 mg, 0.1 mmol) at room temperature. The mixture was heated at 40 °C for 1 h. After cooling to room temperature, the reaction mixture was washed with water and extracted with CH_2Cl_2 for 2 times. After concentration under vacuum, the resulting residue was analyzed by the ^1H NMR spectrum using CH_2Br_2 as an internal standard without further purification. The individual yield of the recovered **1i** and **1i-d_5** cannot be identified from the ^1H NMR spectra due to the complete overlap of the peaks.

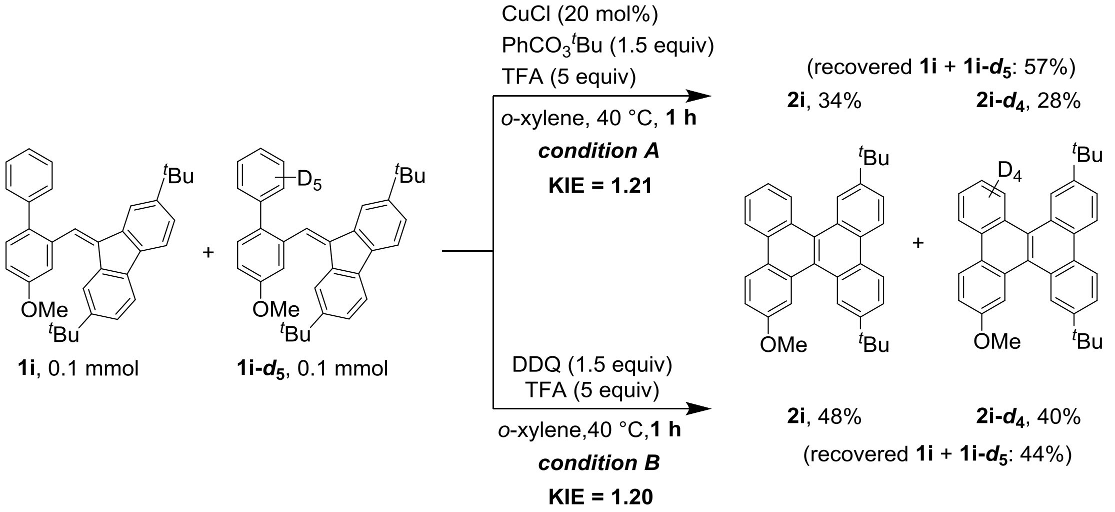

Supplementary Figure 3. Kinetic isotope effects under conditions A and B using **1i** and **1i-d_5** in the same reaction vessel. The ^1H NMR yields were determined by using CH_2Br_2 as an internal standard.

Analytic data

9-(Biphenyl-2-ylmethylene)-9*H*-fluorene (1a)

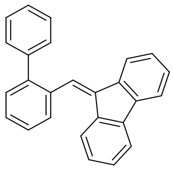

1a

Light yellow solid; 921.9 mg, isolated yield 93%; ^{1}H NMR (400 MHz, $CDCl_3$) δ 7.79–7.73 (m, 3H), 7.68 (d, *J* = 8.0 Hz, 1H), 7.59–7.54 (m, 2H), 7.50 (t, *J* = 7.2 Hz, 1H), 7.45–7.38 (m, 4H), 7.36–7.31 (m, 5H), 7.25 (t, *J* = 7.6 Hz, 1H), 7.12 (dd, *J* = 7.6, 7.2 Hz, 1H); ^{13}C NMR (100 MHz, $CDCl_3$) δ 141.5, 141.1, 140.5, 139.3, 138.8, 136.8, 136.1, 134.8, 131.0, 129.9, 129.5, 128.6, 128.3, 128.10, 128.08, 127.9, 127.3, 127.1, 126.8, 126.6, 124.4, 120.3, 119.7, 119.4; HRMS (MALDI) calcd for $C_{26}H_{18}^+$ $[M]^+$: 330.14030, found: 330.14045.

Dibenzo[*g,p*]chrysene (2a)

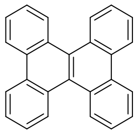

2a

Colourless solid; **Condition A**: 61.8 mg, isolated yield 94%; **Condition B**: 57.2 mg, isolated yield 87%; ^{1}H NMR (400 MHz, $CDCl_3$) δ 8.71 (d, *J* = 8.8 Hz, 8H), 7.71–7.62 (m, 8H); ^{13}C NMR (100 MHz, $CDCl_3$) δ 130.8, 129.1, 128.8, 127.4, 126.5, 123.5 (one peak is not shown due to the complete overlap); HRMS (MALDI) calcd for $C_{26}H_{16}^+$ $[M]^+$: 328.12465, found: 328.12467.

9-(Biphenyl-2-ylmethylene)-2,7-di-*tert*-butyl-9*H*-fluorene (1b)

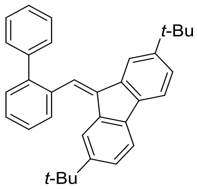

1b

Light yellow solid; 1.208 g, isolated yield 91%; ^{1}H NMR (400 MHz, $CDCl_3$) δ 7.74 (d, *J* = 7.6 Hz, 1H), 7.62–7.31 (m, 15H), 1.35 (s, 9H), 1.18 (s, 9H); ^{13}C NMR (100 MHz, $CDCl_3$) δ 149.6, 149.2, 141.1, 140.7, 139.2, 138.5, 137.3, 137.2, 136.6, 135.3, 131.1, 130.0, 129.5, 128.4, 128.1, 127.2, 126.9, 126.8, 125.3, 121.7, 118.83, 118.80, 117.0, 35.0, 34.8, 31.6, 31.3; HRMS (MALDI) calcd for $C_{34}H_{34}^+$ $[M]^+$: 442.26550, found: 442.26551.

3,14-Di-*tert*-butyldibenzo[*g,p*]chrysene (2b)

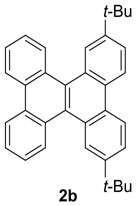

2b

Colourless solid; **Condition A**: 76.7 mg, isolated yield 87%; **Condition B**: 55.5 mg, isolated yield 63%; ^{1}H NMR (400 MHz, $CDCl_3$) δ 8.75–8.73 (m, 6H), 8.63 (d, *J* = 8.8 Hz, 2H), 7.77–7.64 (m, 6H), 1.49 (s, 18H); ^{13}C NMR (100 MHz, $CDCl_3$) δ 148.7, 130.7, 129.4, 128.6, 128.5, 127.8 (×2), 126.34, 126.30, 125.2, 124.3, 123.6, 123.0, 35.1, 31.5; HRMS (MALDI) calcd for $C_{34}H_{32}^{+}$ [M]$^{+}$: 440.24985, found: 440.24983.

9-(Biphenyl-2-ylmethylene)-2,7-dibromo-9*H*-fluorene (1c)

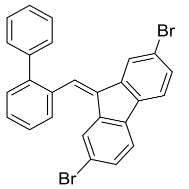

1c

Orange solid; 1.244 g, isolated yield 85%; ^{1}H NMR (400 MHz, $CDCl_3$) δ 7.77 (s, 1H), 7.71 (d, *J* = 7.6 Hz, 1H), 7.67 (s, 1H), 7.58–7.52 (m, 4H), 7.48–7.44 (m, 4H), 7.42–7.34 (m, 5H); ^{13}C NMR (100 MHz, $CDCl_3$) δ 141.8, 140.9, 140.2, 138.9, 138.3, 136.7, 134.2, 133.6, 131.3, 130.9, 130.8, 130.6, 130.2, 129.5, 129.4, 128.2, 127.6, 127.3, 127.2, 123.6, 121.1, 121.0, 120.8, 120.7; HRMS (MALDI) calcd for $C_{26}H_{16}Br_2^{+}$ [M]$^{+}$: 485.96133, found: 485.96132.

3,14-Dibromodibenzo[*g,p*]chrysene (2c)

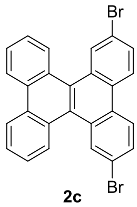

2c

Colourless solid; **Condition A**: 66.2 mg, isolated yield 68%; **Condition B**: 29.2 mg, isolated yield 30%; ^{1}H NMR (400 MHz, $CDCl_3$) δ 8.80 (s, 2H), 8.71 (d, *J* = 8.0 Hz, 2H), 8.60 (d, *J* = 7.2 Hz, 2H), 8.49 (d, *J* = 8.8 Hz, 2H), 7.78–7.67 (m, 6H); ^{13}C NMR (100 MHz, $CDCl_3$) δ 131.1, 131.0, 130.6, 129.7, 128.8, 128.5, 128.3, 127.15, 127.10, 127.0, 125.1, 123.7, 121.0; HRMS (MALDI) calcd for $C_{26}H_{14}Br_2^{+}$ [M]$^{+}$: 483.94568, found: 483.94566.

9-(Biphenyl-2-ylmethylene)-2,7-diiodo-9*H*-fluorene (1d)

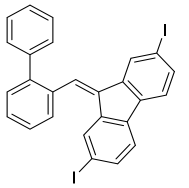

1d

Yellow solid; 1.45 g, isolated yield 83%; ^{1}H NMR (400 MHz, $CDCl_3$) δ 7.95 (s, 1H), 7.88 (s, 1H), 7.70 –7.65 (m, 3H), 7.57–7.52 (m, 2H), 7.47–7.34 (m, 9H); ^{13}C NMR (100 MHz, $CDCl_3$) δ 141.7, 140.7, 140.2, 139.5, 138.2, 137.3, 137.1, 136.8, 134.0, 133.7, 133.2, 130.7, 130.6, 130.2, 129.5, 129.44, 129.35, 128.2, 127.6, 127.2, 121.3, 121.1, 92.5, 92.2; HRMS (MALDI) calcd for $C_{26}H_{16}I_2^{+}$ [M]$^{+}$: 581.93359, found: 581.93361.

3,14-Diiododibenzo[*g,p*]chrysene (2d)

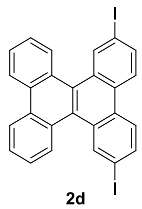

2d

Colourless solid; **Condition A**: 88.2 mg, isolated yield 76%; **Condition B**: 38.3 mg, isolated yield 33%; ^{1}H NMR (400 MHz, CDCl$_3$) δ 9.00 (s, 2H), 8.70 (d, *J* = 7.6 Hz, 2H), 8.57 (d, *J* = 7.6 Hz, 2H), 8.34 (d, *J* = 8.4 Hz, 2H), 7.94 (dd, *J* = 8.4, 1.6 Hz, 2H), 7.74–7.67 (m, 4H); ^{13}C NMR (100 MHz, CDCl$_3$) δ 137.2, 135.3, 131.0, 130.8, 129.3, 128.4, 128.3, 127.11, 127.06, 126.7, 125.0, 123.7, 92.6; HRMS (MALDI) calcd for $C_{26}H_{14}I_2^+$ [M]$^+$: 579.91794, found: 579.91791.

9-((4,4'-Dimethyl-biphenyl-2-yl)methylene)-9*H*-fluorene (1e)

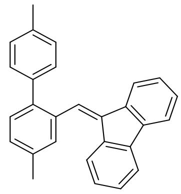

1e

Light yellow solid; 978.7 mg, isolated yield 91%; ^{1}H NMR (400 MHz, CDCl$_3$) δ 7.78 –7.72 (m, 3H), 7.60 (d, *J* = 6.8 Hz, 2H), 7.45–7.43 (m, 2H), 7.38–7.24 (m, 6H), 7.15 –7.12 (m, 3H), 2.42 (s, 3H), 2.36 (s, 3H); ^{13}C NMR (100 MHz, CDCl$_3$) δ 141.1, 139.4, 138.8, 138.7, 137.6, 136.9, 136.8, 136.5, 135.8, 134.5, 131.4, 129.7, 129.4, 129.3, 128.8, 128.6, 128.2, 127.8, 126.8, 126.6, 124.3, 120.3, 119.7, 119.4, 21.2, 21.0; HRMS (MALDI) calcd for $C_{28}H_{22}^+$ [M]$^+$: 358.17160, found: 358.17165.

3,14-Dimethyldibenzo[*g,p*]chrysene (2e)

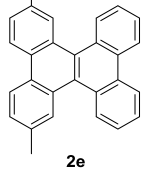

2e

Light yellow solid; **Condition A**: 58.5 mg, isolated yield 82%; **Condition B**: 59.2 mg, isolated yield 83%; ^{1}H NMR (400 MHz, CDCl$_3$) δ 8.72 (dt, *J* = 8.0, 2.0 Hz, 4H), 8.56 (d, *J* = 8.4 Hz, 2H), 8.49 (s, 2H), 7.70–7.63 (m, 4H), 7.50 (dd, *J* = 8.4, 1.2 Hz, 2H), 2.59 (s, 6H); ^{13}C NMR (100 MHz, CDCl$_3$) δ 135.6, 130.6, 129.3, 128.9, 128.7, 128.6, 128.5, 127.9, 127.4, 126.33, 126.27, 123.5, 123.2, 21.9; HRMS (MALDI) calcd for $C_{28}H_{20}^+$ [M]$^+$: 356.15595, found: 356.15595.

9-((4,4'-Dimethoxy-biphenyl-2-yl)methylene)-9*H*-fluorene (1f)

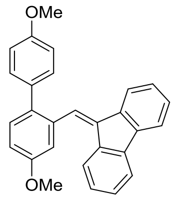

1f

Light yellow solid; 1.078 g, isolated yield 92%; ^{1}H NMR (400 MHz, $CDCl_3$) δ 7.79 –7.73 (m, 3H), 7.60 (d, *J* = 7.6 Hz, 1H), 7.45–7.41 (m, 2H), 7.38–7.31 (m, 5H), 7.28 –7.24 (m, 1H), 7.14 (t, *J* = 7.6 Hz, 1H), 7.04 (dd, *J* = 2.8, 8.8 Hz, 1H), 6.86 (d, *J* = 8.8 Hz, 2H), 3.82 (s, 3H), 3.80 (s, 3H); ^{13}C NMR (100 MHz, $CDCl_3$) δ 158.6, 158.1, 141.1, 139.3, 138.8, 136.7, 136.0, 135.5, 133.9, 132.7, 130.8, 130.6, 128.4, 128.3, 127.9, 126.8, 126.6, 124.6, 120.4, 119.7, 119.4, 115.21, 115.19, 113.5, 55.6, 55.2; HRMS (MALDI) calcd for $C_{28}H_{22}O_2^+$ $[M]^+$: 390.16143, found: 390.16145.

3,14-Dimethoxydibenzo[*g,p*]chrysene (2f)

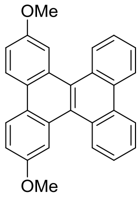

2f

Light yellow solid; **Condition A**: 56.0 mg, isolated yield 72%; **Condition B**: 50.5 mg, isolated yield 65%; ^{1}H NMR (400 MHz, $CDCl_3$) δ 8.79 (dd, *J* = 8.0, 1.2 Hz, 2H), 8.70 (dd, *J* = 8.0, 1.2 Hz, 2H), 8.52 (d, *J* = 8.8 Hz, 2H), 8.14 (d, *J* = 2.8 Hz, 2H), 7.70–7.60 (m, 4H), 7.29 (dd, *J* = 8.8, 2.8 Hz, 2H), 3.94 (s, 6H); ^{13}C NMR (100 MHz, $CDCl_3$) δ 157.5, 130.7, 129.5, 129.3, 128.1, 127.9, 126.5, 126.4, 125.1, 124.5, 123.6, 115.7, 111.0, 55.6; HRMS (MALDI) calcd for $C_{28}H_{20}O_2^+$ $[M]^+$: 388.14578, found: 388.14579.

9-((4,4'-Difluoro-biphenyl-2-yl)methylene)-9*H*-fluorene (1g)

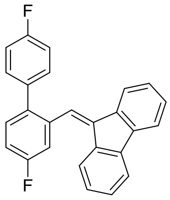

1g

Light yellow solid; 1.022 g, isolated yield 93%; ^{1}H NMR (400 MHz, $CDCl_3$) δ 7.73 (t, *J* = 8.4 Hz, 2H), 7.61 (d, *J* = 8.0 Hz, 1H), 7.56 (d, *J* = 7.6 Hz, 1H), 7.48–7.44 (m, 2H), 7.39–7.32 (m, 4H), 7.28 –7.24 (m, 2H), 7.21 –7.13 (m, 2H), 7.01 (t, *J* = 8.8 Hz, 2H); ^{13}C NMR (100 MHz, $CDCl_3$) δ 162.2 (d, J^1 = 245.8 Hz), 161.6 (d, J^1 = 245.9 Hz), 141.1, 139.0 (d, J^3 = 9.9 Hz), 137.2, 136.7 (d, J^3 = 8.2 Hz), 136.62, 136.59, 136.2, 135.5 (d, J^4 = 3.3 Hz), 131.4 (d, J^3 = 8.3 Hz), 131.1 (d, J^3 = 8.3 Hz), 128.8, 128.4, 127.0, 126.9, 125.9 (d, J^4 = 1.7 Hz), 124.3, 120.3, 119.9, 119.6, 117.4 (d, J^2 = 20.5 Hz), 115.5 (d, J^2 = 21.4 Hz), 115.1 (d, J^2 = 20.6 Hz); HRMS (MALDI) calcd for $C_{26}H_{16}F_2^+$ $[M]^+$: 366.12146, found: 366.12149.

3,14-Difluorodibenzo[*g,p*]chrysene (2g)

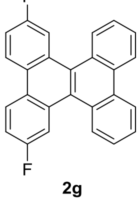

2g

Colourless solid; **Condition A**: 51.0 mg, isolated yield 70%; **Condition B**: 14.6 mg, isolated yield 20%;

^{1}H NMR (400 MHz, $CDCl_3$) δ 8.70 (d, *J* = 7.6 Hz, 2H), 8.63 (d, *J* = 8.4 Hz, 2H), 8.59–8.55 (m, 2H), 8.32 (dd, *J* = 11.6, 2.4, Hz, 2H), 7.71 (t, *J* = 7.2 Hz, 2H), 7.65 (t, *J* = 7.2 Hz, 2H), 7.40 (dt, *J* = 8.0, 2.4 Hz, 2H); ^{13}C NMR (100 MHz, $CDCl_3$) δ 161.3 (d, J^1 = 242.8 Hz), 130.9, 130.3 (d, J^3 = 9.9 Hz), 128.8, 128.2, 127.3 (d, J^4 = 3.3 Hz), 127.1, 127.0, 126.9, 125.6 (d, J^3 = 9.9 Hz), 123.7, 115.2 (d, J^2 = 23.0 Hz), 114.0 (d, J^2 = 23.0 Hz); HRMS (MALDI) calcd for $C_{26}H_{14}F_2^+$ $[M]^+$: 364.10581, found: 364.10582.

9-((4,4'-Dichloro-biphenyl-2-yl)methylene)-9*H*-fluorene (1h)

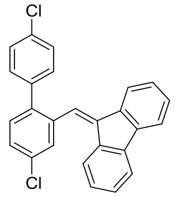

1h

Light yellow solid; 1.114 g, isolated yield 93%; ^{1}H NMR (400 MHz, $CDCl_3$) δ 7.71–7.67 (m, 3H), 7.54–7.51 (m, 2H), 7.43–7.37 (m, 2H), 7.33 (t, *J* = 7.2 Hz, 2H), 7.28–7.20 (m, 6H), 7.11 (t, *J* = 8.0 Hz, 1H); ^{13}C NMR (100 MHz, $CDCl_3$) δ 141.4, 139.0, 138.8, 138.6, 137.8, 137.4, 136.5, 136.2, 133.8, 133.3, 131.0, 130.7, 130.6, 128.9, 128.6, 128.4, 127.0, 126.9, 125.5, 124.2, 120.4, 119.9, 119.6; HRMS (MALDI) calcd for $C_{26}H_{16}Cl_2^+$ $[M]^+$: 398.06236, found: 398.06239.

3,14-Dichlorodibenzo[*g,p*]chrysene (2h)

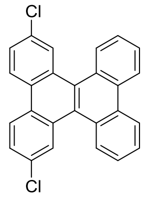

2h

Colourless solid; **Condition A**: 44.5 mg, isolated yield 56%; **Condition B**: 17.5 mg, isolated yield 22%; ^{1}H NMR (400 MHz, CD_2Cl_2) δ 8.72 (d, *J* = 8.0 Hz, 2H), 8.63–8.56 (m, 6H), 7.75–7.63 (m, 6H); ^{13}C NMR (100 MHz, CD_2Cl_2) δ 133.0, 131.2, 130.5, 128.83, 128.81, 128.6, 128.2, 127.6, 127.5 (×2), 127.3, 125.5, 124.0; HRMS (MALDI) calcd for $C_{26}H_{14}Cl_2^+$ $[M]^+$: 396.04671, found: 396.04673.

2,7-Di-*tert*-butyl-9-((4-methoxy-biphenyl-2-yl)methylene)-9H-fluorene (1i)

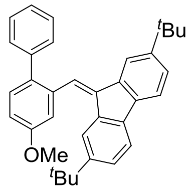

1i

Yellow solid; 1.26 g, isolated yield 89%; ^{1}H NMR (400 MHz, $CDCl_3$) δ 7.61-7.56 (m, 4H), 7.49 (d, *J* = 8.8 Hz, 1H), 7.45-7.44 (m, 3H), 7.39 (d, *J* = 9.2 Hz, 1H), 7.35-7.27 (m, 4H), 7.25 (d, *J* = 2.4 Hz, 1H), 7.06 (dd, *J* = 8.0, 2.4 Hz, 1H), 3.79 (s, 3H), 1.34 (s, 9H), 1.18 (s, 9H); ^{13}C NMR (100 MHz, $CDCl_3$) δ; 158.5, 149.7, 149.3, 140.5, 139.2, 138.6, 137.5, 137.1, 136.7, 136.3, 133.7, 131.2, 129.5, 128.1, 126.9, 126.8, 125.4(4), 125.4(2), 121.9, 118.9, 118.8, 117.1, 115.3, 114.9, 55.4, 34.9, 34.7, 31.5, 31.2; HRMS (MALDI) calcd for $C_{35}H_{36}O^+$ $[M]^+$: 472.27607, found: 472.27607.

6,11-Di-*tert*-butyl-3-methoxydibenzo[*g,p*]chrysene (2i)

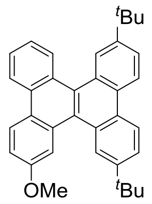

2i

Yellow solid; **Condition A**: 66 mg, isolated yield 70%; **Condition B**: 60.2 mg, isolated yield 64%; ^{1}H NMR (400 MHz, $CDCl_3$) δ 8.79 (d, *J* = 2.0 Hz, 1H), 8.71 (d, *J* = 1.6 Hz, 1H), 8.69 (d, *J* = 8.0 Hz, 1H), 8.65-8.60 (m, 4H), 8.19 (d, *J* = 2.4 Hz, 1H), 7.74 (dt, *J* = 6.4, 2.0 Hz, 2H), 7.68-7.64 (m, 1H), 7.61-7.57 (m, 1H), 7.32 (dd, *J* = 8.8, 2.4 Hz, 1H), 3.96 (s, 3H), 1.47 (s, 9H), 1.46 (s, 9H); ^{13}C NMR (100 MHz, $CDCl_3$) δ 158.2, 148.8(4), 148.8(2), 130.9, 130.7, 128.9, 128.7(7), 128.7(4), 128.6, 128.5, 127.6, 126.5, 125.4(6), 125.3(9), 125.2, 124.9, 124.4(9), 124.4(7), 124.3(7), 123.2, 123.1, 123.0, 116.3, 109.9, 55.4, 35.0(7), 35.0(2), 31.5, 31.4, two aromatic peaks are not shown due to superimposition; HRMS (MALDI) calcd for $C_{35}H_{34}O^+$ [M]$^+$: 470.26042, found: 470.26043.

2,7-Di-*tert*-butyl-9-((4-methoxy-biphenyl-2-yl-2',3',4',5',6'-*d*$_5$)methylene)-9*H*-fluorene (1i-*d*$_5$)

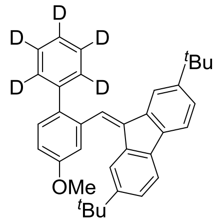

1i-*d*$_5$

Yellow solid; 1.23 g, isolated yield 86%; ^{1}H NMR (400 MHz, $CDCl_3$) δ 7.66 (d, *J* = 8.0 Hz, 4H), 7.55 (d, *J* = 8.4 Hz, 1H), 7.51 (s, 1H), 7.44 (d, *J* = 7.6 Hz, 1H), 7.40 (d, *J* = 8.4 Hz, 1H), 7.31 (d, *J* = 2.0 Hz, 1H), 7.11 (dd, *J* = 8.0, 2.0 Hz,1H), 3.83 (s, 3H), 1.40 (s, 9H), 1.24 (s, 9H); ^{13}C NMR (100 MHz, $CDCl_3$) δ 158.5, 149.7, 149.2, 140.2, 139.2, 138.6, 137.5, 137.1, 136.7, 136.2, 133.6, 131.2, 129.1 (weak t, *J* = 24 Hz), 127.6 (weak t, *J* = 24 Hz), 126.9, 126.3 (weak t, *J* = 24 Hz), 125.4(5), 125.4(3), 121.9, 118.9(2), 118.8(9), 117.1, 115.3, 114.9, 55.3, 34.9, 34.7, 31.5, 31.2; HRMS (MALDI) calcd for $C_{35}H_{31}D_5O^+$ [M]$^+$: 477.30745, found: 477.30745.

6,11-Di-*tert*-butyl-3-methoxydibenzo[*g,p*]chrysene-13,14,15,16-d$_4$ (2i-*d*$_4$)

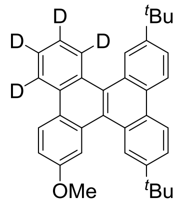

2i-*d*$_4$

Yellow solid; **Condition A**: 57 mg, isolated yield 60%; **Condition B**: 68.3 mg, isolated yield 72%; ^{1}H NMR (400 MHz, $CDCl_3$) δ 8.81 (s, 1H), 8.72 (s, 1H), 8.65-8.61 (m, 3H), 8.20 (d, *J* = 2.0 Hz, 1H), 7.75 (d, *J* = 8.8 Hz, 2H), 7.33 (dd, *J* = 8.8, 2.4 Hz, 1H), 3.97 (s, 3H), 1.48(3) (s, 9H), 1.48(1) (s, 9H); ^{13}C NMR (100 MHz, $CDCl_3$) δ 158.2, 148.8, 148.7, 130.8, 130.7, 128.9, 128.7, 128.6, 128.4(8), 128.4(6), 128.3

(weak t, J = 24 Hz), 127.6(1), 127.5(9), 126.0 (weak t, J = 24 Hz), 125.3, 125.2, 124.9 (weak t, J = 24 Hz), 124.8, 124.4, 124.3, 123.2, 123.0, 122.7 (weak t, J = 24 Hz), 116.3, 109.8, 55.3, 35.0(5), 35.0(1), 31.5, 31.4, one aromatic peak is not shown due to superimposition; HRMS (MALDI) calcd for $C_{35}H_{30}D_4O^+$ $[M]^+$: 474.28552, found: 474.28551.

2-(2-((9*H*-Fluoren-9-ylidene)methyl)phenyl)benzo[*b*]thiophene (1j)

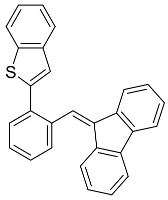

1j

Light yellow solid; 997.2 mg, isolated yield 86%; ^{1}H NMR (400 MHz, $CDCl_3$) δ 7.82–7.76 (m, 5H), 7.73–7.68 (m, 3H), 7.60 (d, J = 8.0 Hz, 1H), 7.52 (dd, J = 8.0, 7.2 Hz, 1H), 7.46–7.28 (m, 7H), 7.11 (dd, J = 8.0, 7.6 Hz, 1H); ^{13}C NMR (100 MHz, $CDCl_3$) δ 142.5, 141.3, 140.3, 140.2, 139.3, 139.1, 136.9, 136.7, 135.1, 133.9, 131.5, 130.0, 128.7, 128.6, 128.2, 128.0, 127.4, 127.1, 126.7, 124.5, 124.39, 124.36, 124.3, 123.8, 122.0, 120.6, 119.8, 119.6; HRMS (MALDI) calcd for $C_{28}H_{18}S^+$ $[M]^+$: 386.11237, found: 386.11239.

Benzo[*b*]benzo[11,12]chryseno[5,6-*d*]thiophene (2j)

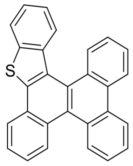

2j

brown solid; **Condition A**: 17.7 mg, isolated yield 23%; **Condition B**: 42.3 mg, isolated yield 55%; ^{1}H NMR (400 MHz, $CDCl_3$) δ 8.34–8.30 (m, 2H), 7.82 (d, J = 7.6 Hz, 1H), 7.76 (d, J = 7.2 Hz, 1H), 7.73 (d, J = 7.6 Hz, 1H), 7.66 (d, J = 7.6 Hz, 1H), 7.59 (d, J = 7.6 Hz, 1H), 7.42 (d, J = 7.2 Hz, 1H), 7.39–7.31 (m, 2H), 7.29–7.12 (m, 6H); ^{13}C NMR (100 MHz, $CDCl_3$) δ 150.3, 143.8, 141.93, 141.87, 140.7, 139.1, 138.6, 138.4, 136.9, 136.3, 134.8, 133.6, 129.1, 129.0, 128.9, 128.5, 128.0, 126.9, 126.8, 126.7, 126.0, 125.5, 124.8, 124.0, 123.9, 120.2, 120.0, 119.7; HRMS (MALDI) calcd for $C_{28}H_{16}S^+$ $[M]^+$: 384.09672, found: 384.09674.

9-([1,1'-Binaphthalen]-2-ylmethylene)-9*H*-fluorene (1k)

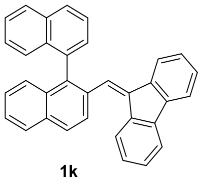

1k

Light yellow solid; 1.136 g, isolated yield 88%; ^{1}H NMR (400 MHz, $CDCl_3$) δ 8.02 (dd, J = 8.4, 6.8 Hz, 2H), 7.98–7.93 (m, 3H), 7.72 (d, J = 7.6 Hz, 1H), 7.65 (dd, J = 8.8, 7.6 Hz, 2H), 7.57–7.43 (m, 4H), 7.39–7.24 (m, 8H), 7.12 (t, J = 7.6 Hz, 2H); ^{13}C NMR (100 MHz, $CDCl_3$) δ 141.1, 139.3, 138.8, 137.8, 136.6, 136.2, 136.1, 134.0, 133.5, 133.3, 133.2, 132.2, 128.6, 128.31, 128.29, 128.2, 128.1, 127.9, 127.8, 127.6, 127.2, 126.8, 126.6, 126.5, 126.3, 126.2, 126.1, 126.0, 1258, 125.5, 124.6, 120.2,

119.6, 119.3; HRMS (MALDI) calcd for $C_{34}H_{22}^+$ [M]$^+$: 430.17160, found: 430.17162.

Benzo[*f*]naphtho[1,2-*s*]picene (2k)

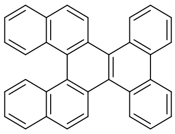

2k

Light yellow solid; **Condition A**: 73.7 mg, isolated yield 86%; **Condition B**: 79.7 mg, isolated yield 93%; ^{1}H NMR (400 MHz, CD_2Cl_2) δ 8.79–8.72 (m, 6H), 8.40 (d, *J* = 8.8 Hz, 2H), 8.07 (d, *J* = 8.8 Hz, 2H), 7.98 (d, *J* = 8.0 Hz, 2H), 7.74–7.71 (m, 4H), 7.54–7.50 (m, 2H), 7.34–7.30 (m, 2H); ^{13}C NMR (100 MHz, CD_2Cl_2) δ 132.2, 131.4, 131.1, 129.4, 129.2, 129.1, 129.0, 128.9, 128.5, 128.3, 127.0 (×2), 126.5, 126.2, 125.3, 125.2, 123.9; HRMS (MALDI) calcd for $C_{34}H_{20}^+$ [M]$^+$: 428.15595, found: 428.15593.

2,6-Bis((9*H*-fluoren-9-ylidene)methyl)-1,1'-biphenyl (1l)

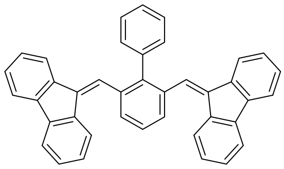

1l

Light yellow solid; 1.353 g, isolated yield 89%; ^{1}H NMR (400 MHz, $CDCl_3$) δ 7.84 (d, *J* = 7.6 Hz, 2H), 7.78–7.72 (m, 6H), 7.52–7.46 (m, 3H), 7.39–7.32 (m, 6H), 7.28–7.21 (m, 7H), 7.16 (dd, *J* = 8.0, 7.2 Hz, 2H); ^{13}C NMR (100 MHz, $CDCl_3$) δ 141.2, 141.1, 139.3, 138.8, 138.4, 136.8, 136.4, 136.2, 130.9, 130.5, 128.4, 128.0, 127.8, 127.64, 127.59, 127.0, 126.8, 126.7, 124.4, 120.3, 119.8, 119.4; HRMS (MALDI) calcd for $C_{40}H_{26}^+$ [M]$^+$: 506.20290, found: 506.20290.

Hexabenzo[*a,c,fg,j,l,op*]tetracene (2l)

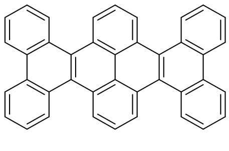

2l

Yellow solid; **Condition A**: 10.1 mg, isolated yield 10%; **Condition B**: 55.3 mg, isolated yield 55%; ^{1}H NMR (400 MHz, $CDCl_3$) δ 9.01 (d, *J* = 7.6 Hz, 4H), 8.91 (d, *J* = 8.0 Hz, 4H), 8.80 (d, *J* = 8.4 Hz, 4H), 8.11 (t, *J* = 8.0 Hz, 2H), 7.76 (dd, *J* = 8.0, 7.2 Hz, 4H), 7.67 (dd, *J* = 8.0, 7.2 Hz, 4H); ^{13}C NMR (100 MHz, $CDCl_3$) δ 131.1, 129.6, 128.7, 128.3, 127.9, 126.9, 126.7, 125.9, 125.5, 124.5, 123.7; HRMS (MALDI) calcd for $C_{40}H_{22}^+$ [M]$^+$: 502.17160, found: 502.17159.

1-((9*H*-Fluoren-9-ylidene)methyl)dibenzo[*g,p*]chrysene (2l')

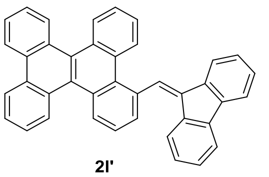

2l'

Orange solid; **Condition A**: 60.6 mg, isolated yield 60%; **Condition B**: 16.1 mg, isolated yield 16%; ^{1}H NMR (400 MHz, CD_2Cl_2) δ 8.78–8.71 (m, 6H), 8.66 (d, *J* = 8.0 Hz, 1H), 8.10 (s, 1H), 7.99 (t, *J* = 6.8 Hz, 2H), 7.88–7.84 (m, 3H), 7.75–7.65 (m, 5H), 7.59 (dd, *J* = 8.0, 7.2 Hz, 1H), 7.48–7.37 (m, 4H), 7.15 (t, *J* = 7.6 Hz, 1H); ^{13}C NMR (100 MHz, CD_2Cl_2) δ 141.5, 140.0, 139.3, 137.5, 135.1, 134.5, 131.5, 131.4, 131.3, 131.1, 131.0, 130.9, 130.6, 129.5, 129.4, 129.3, 129.2, 129.05, 128.98, 128.9, 128.54, 128.48, 128.2, 128.0, 127.51, 127.48, 127.18, 127.15, 127.1, 127.04, 127.01, 126.8, 126.0, 124.8, 124.0, 123.95, 120.8, 120.2, 120.0; HRMS (MALDI) calcd for $C_{40}H_{24}^+$ $[M]^+$: 504.18725, found: 504.18725.

2',5'-Bis((9*H*-fluoren-9-ylidene)methyl)-1,1':4',1''-terphenyl (1m)

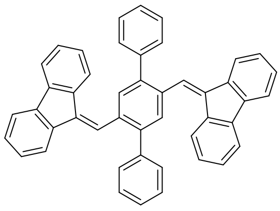

1m

Yellow solid; 1.486 g, isolated yield 85%; ^{1}H NMR (400 MHz, $CDCl_3$) δ 8.03–8.01 (m, 4H), 7.81 (d, *J* = 8.0 Hz, 2H), 7.77 (d, *J* = 7.2 Hz, 2H), 7.64 (d, *J* = 7.2 Hz, 2H), 7.55 (s, 2H), 7.50 (d, *J* = 6.4 Hz, 4H), 7.44–7.37 (m, 4H), 7.34–7.30 (m, 6H), 7.29–7.24 (m, 4H); ^{13}C NMR (100 MHz, $CDCl_3$) δ 141.4, 140.1, 139.7, 139.4, 139.0, 136.9, 136.7, 135.2, 132.9, 129.6, 128.7, 128.3, 128.2, 127.62, 127.59, 127.0, 126.8, 124.4, 120.5, 120.0, 119.6; HRMS (MALDI) calcd for $C_{46}H_{30}^+$ $[M]^+$: 582.23420, found: 582.23419.

Tetrabenzo[*a,c,f,m*]phenanthro[9,10-*k*]tetraphene (2m)

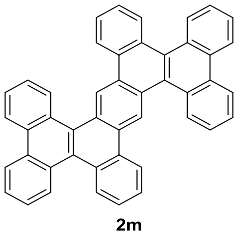

2m

Yellow solid; **Condition A**: 23.2 mg, isolated yield 20%; **Condition B**: 101.9 mg, isolated yield 88%; **Condition C**: 110 mg, isolated yield 95%; ^{1}H NMR (400 MHz, CD_2Cl_2) δ 9.52 (s, 2H), 8.85 (d, *J* = 7.6 Hz, 2H), 8.72 (dd, *J* = 7.6, 8.4 Hz, 4H), 8.54–8.49 (m, 6H), 7.77–7.65 (m, 6H), 7.60–7.52 (m, 6H); ^{13}C NMR (100 MHz, CD_2Cl_2) δ 131.5, 131.4, 131.2, 130.0, 129.9, 129.8, 129.5 (×2), 129.2, 129.1, 128.3, 128.2, 128.1, 127.5, 127.3, 127.24, 127.19, 127.1, 127.0, 124.5, 124.2, 124.1, 124.0; HRMS (MALDI) calcd for $C_{46}H_{26}^+$ $[M]^+$: 578.20290, found: 578.20291.

2',5'-Bis((2,7-di-*tert*-butyl-9*H*-fluoren-9-ylidene)methyl)-1,1':4',1''-terphenyl (1n)

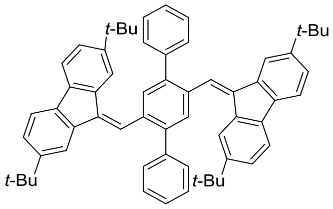

1n

Yellow solid; 407 mg, isolated yield 84%; ^{1}H NMR (400 MHz, $CDCl_3$) δ 8.07 (s, 2H), 7.96 (d, *J* = 1.2 Hz, 2H), 7.63–7.61 (m, 6H), 7.53–7.50 (m, 6H), 7.40 (dd, *J* = 8.4, 8.0 Hz, 2H), 7.35–7.29 (m, 8H), 1.35 (s, 18H), 1.03 (s, 18H); ^{13}C NMR (100 MHz, $CDCl_3$) δ 149.8, 149.5, 140.0, 139.7, 139.4, 138.8, 137.5, 137.1, 136.6, 135.2, 133.2, 129.7, 128.2, 127.5, 126.5, 125.7, 125.5, 121.4, 119.0, 118.9, 117.1, 34.9, 34.6, 31.5, 31.2; HRMS (MALDI) calcd for $C_{62}H_{62}^+$ [M]$^+$: 806.48460, found: 806.48463.

3,11,16,24-Tetra-*tert*-butyltetrabenzo[*a,c,f,m*]phenanthro[9,10-*k*]tetraphene (2n)

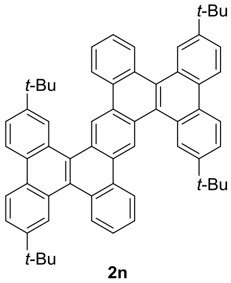

2n

Yellow solid; **Condition B**: 65.8 mg, isolated yield 41%; **Condition C**: 138 mg, isolated yield 86%; ^{1}H NMR (400 MHz, $CDCl_3$) δ 10.03 (s, 2H), 9.12 (s, 2H), 8.90–8.88 (m, 2H), 8.75–8.74 (m, 4H), 8.72 (d, *J* = 8.8 Hz, 2H), 8.68 (d, *J* = 8.8 Hz, 2H), 7.86 (dd, *J* = 8.8, 8.8 Hz, 2H), 7.77 (dd, *J* = 8.8, 8.8 Hz, 2H), 7.70–7.64 (m, 4H), 1.57 (s, 18H), 1.46 (s, 18); ^{13}C NMR (100 MHz, $CDCl_3$) δ 149.3, 148.9, 131.2, 130.0, 129.4, 129.1, 128.9 (×2), 128.7, 128.6, 128.4, 128.3, 128.0, 126.6, 126.5 (×2), 125.2 (×2), 124.7, 124.6, 123.9, 123.8, 123.3, 123.2, 35.2, 35.1, 31.7, 31.4; HRMS (MALDI) calcd for $C_{62}H_{58}^+$ [M]$^+$: 802.45330, found: 802.45325.

4',6'-Bis((9*H*-fluoren-9-ylidene)methyl)-1,1':3',1''-terphenyl (1o)

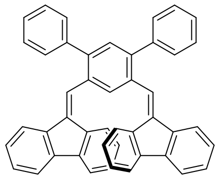

1o

Yellow solid; 955 mg, isolated yield 82%; ^{1}H NMR (400 MHz, $CDCl_3$) δ 8.08 (s, 1H), 7.74 (s, 1H), 7.70-7.67 (m, 6H), 7.60-7.56 (m, 6H), 7.49 (s, 2H), 7.40-7.22 (m, 12H), 6.96-6.92 (m, 2H); ^{13}C NMR (100 MHz, $CDCl_3$) δ 141.6, 141.1, 140.1, 139.3, 139.0, 136.9, 136.7, 134.1, 132.6, 131.6, 129.6, 128.4, 128.3, 128.0, 127.6, 126.9, 126.8(5), 126.8(0), 124.4, 120.4, 119.7, 119.5; HRMS (MALDI) calcd for $C_{46}H_{30}^+$ [M]$^+$: 582.23420, found: 582.23418.

Tetrabenzo[*a,c,f,k*]phenanthro[9,10-*m*]tetraphene (2o)

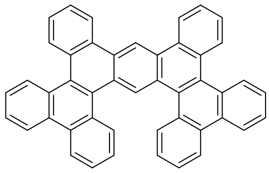

2o

Yellow solid; **Condition A**: 37 mg, isolated yield 32%; **Condition B**: 99.5 mg, isolated yield 86%; ^{1}H NMR (400 MHz, $C_2D_2Cl_4$) δ 9.94 (s, 1H), 9.67 (s, 1H), 9.08 (d, *J* = 8.0 Hz, 2H), 8.95 (d, *J* = 8.4 Hz, 2H),

8.75-8.70 (m, 8H), 7.86 (dd, *J* = 7.6, 7.6 Hz, 4H), 7.76 (m, 4H), 7.72-7.64 (m, 4H); ^{13}C NMR (176 MHz, $C_2D_2Cl_4$) δ 131.1, 130.7, 130.6, 129.5, 129.0, 128.9(7), 128.9(1), 128.7, 128.6, 128.5, 128.1, 127.9, 127.6, 127.1, 126.9(7), 126.9(3), 126.7, 126.6, 125.7, 124.2, 123.6, 123.5, 123.4, 120.2; HRMS (MALDI) calcd for $C_{46}H_{26}^+$ [M]$^+$: 578.20290, found: 578.20290.

9,9'-((1,5-Diphenylnaphthalene-2,6-diyl)bis(methaneylylidene))bis(2,7-di-*tert*-butyl-9*H*-fluorene) (1p)

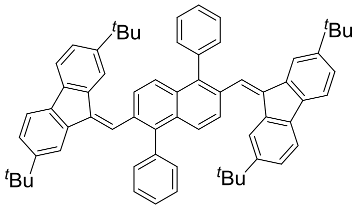

1p

Yellow solid; 617 mg, isolated yield 72%; ^{1}H NMR (400 MHz, $CDCl_3$) δ 7.80 (d, *J* = 8.8 Hz, 2H), 7.65 (d, *J* = 8.8 Hz, 2H), 7.59 (d, *J* = 8.4 Hz, 2H), 7.58 (d, *J* = 7.6 Hz, 2H), 7.51-7.42 (m, 14H), 7.37-7.32 (m, 6H), 1.33 (s, 18H), 1.15 (s, 18H); ^{13}C NMR (176 MHz, $CDCl_3$) δ 149.6, 149.2, 139.3(6), 139.3(3), 138.6(2), 138.5(9), 137.3, 137.0, 136.7, 133.4, 132.6, 130.6, 128.7, 128.2, 127.5, 126.4, 125.7, 125.5, 125.3, 121.9, 118.9(2), 118.8(7), 116.9; HRMS (MALDI) calcd for $C_{66}H_{64}^+$ [M]$^+$: 856.50025, found: 856.50028.

3,12,17,26-Tetra-*tert*-butyltetrabenzo[*a,c,f,o*]phenanthro[9,10-*m*]picene (2p)

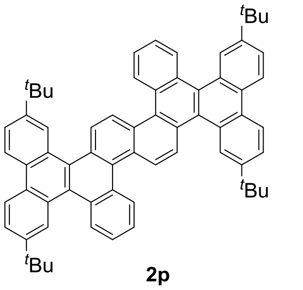

2p

Yellow solid; **Condition B**: 133 mg, isolated yield 72%; ^{1}H NMR (400 MHz, $CDCl_3$) δ 9.19 (d, *J* = 9.2 Hz, 2H), 9.15 (d, *J* = 7.6 Hz, 2H), 8.97 (d, *J* = 2.0 Hz, 2H), 8.82 (d, *J* = 8.4 Hz, 2H), 8.78 (d, *J* = 2.0 Hz, 2H), 8.75 (d, *J* = 9.2 Hz, 2H), 8.66 (d, *J* = 8.8 Hz, 2H), 8.63 (d, *J* = 10.0 Hz, 2H), 7.82-7.78 (m, 4H), 7.76-7.70 (m, 4H), 1.61 (s, 18H), 1.47 (s, 18H); ^{13}C NMR (100 MHz, $CDCl_3$) δ 149.1, 148.8, 130.6, 130.3, 129.9, 129.8, 128.8, 128.7, 128.6, 128.5, 128.3, 128.1, 127.9(8), 127.9(6), 127.9(3), 127.6, 126.6, 125.8, 125.6, 125.1, 124.7, 124.5, 123.2, 123.1, 35.2, 35.0, 31.6, 31.4, one aromatic peak is not shown due to superimposition; HRMS (MALDI) calcd for $C_{66}H_{60}^+$ [M]$^+$: 852.46895, found: 852.46902.

2',6'-Bis((2,7-di-*tert*-butyl-9*H*-fluoren-9-ylidene)methyl)-1,1':5',1''-ternaphthalene (1q)

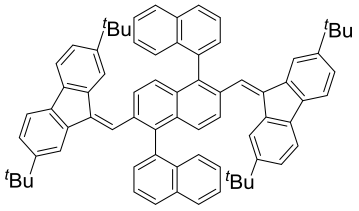

1q

Yellow solid; a 4.2:1 mixture of *cis*- and *trans*-isomers (the major and minor isomers were not determined); 1.15 g, isolated yield 80%; major isomer: ^{1}H NMR (400 MHz, $CDCl_3$) δ 7.96 (d, *J* = 8.0 Hz, 2H), 7.94 (d, *J* = 7.6 Hz, 2H), 7.71 (d, *J* = 8.8 Hz, 2H), 7.63-7.55 (m, 4H), 7.52-7.42 (m, 12H), 7.32-7.25 (m, 10H), 1.25 (s, 18H) [minor: 1.24 (s, 18H)], 1.12 (s, 18H) [minor: 1.11 (s, 18H)]; ^{13}C NMR (100 MHz, $CDCl_3$) δ 149.4, 149.0, 139.1, 138.4, 137.5, 137.4, 136.8, 136.7, 136.5, 134.8, 133.6, 133.2, 132.6, 129.1, 128.3, 128.2, 128.0, 126.3, 126.2, 126.1, 125.8, 125.5, 125.3, 125.2, 121.9, 118.8, 118.7, 116.9, 34.8, 34.7, 31.4, 31.3, one aromatic peak is not shown due to superimposition; HRMS (MALDI) calcd for $C_{74}H_{68}^{+}$ [M]$^{+}$: 956.53155, found: 956.53158.

Compound 2q

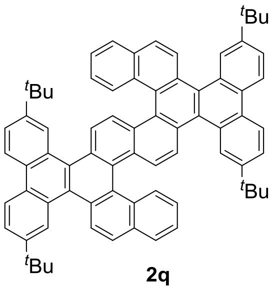

2q

Yellow solid; **Condition B**: 74.3 mg, isolated yield 40%; ^{1}H NMR (700 MHz, $CDCl_3$) δ 9.02 (d, *J* = 8.4 Hz, 2H), 8.91 (d, *J* = 2.1 Hz, 2H), 8.89 (d, *J* = 9.1 Hz, 2H), 8.82 (d, *J* = 2.1 Hz, 2H), 8.61 (d, *J* = 8.4 Hz, 4H), 8.47 (d, *J* = 9.1 Hz, 2H), 8.41 (d, *J* = 9.1 Hz, 2H), 8.14 (d, *J* = 9.1 Hz, 2H), 8.07 (dd, *J* = 8.4, 0.7 Hz, 2H), 7.77-7.74 (m, 4H), 7.67-7.64 (m, 2H), 7.55-7.53 (m, 2H), 1.54 (s, 18H), 1.50 (s, 18H); ^{13}C NMR (176 MHz, $CDCl_3$) δ 149.0, 148.9, 132.1, 131.1, 130.7, 130.0, 129.6, 129.4, 129.3, 129.1, 128.9, 128.8, 128.5, 128.4, 128.3, 128.2, 128.1, 126.3, 126.2(9), 126.2(7), 125.4(7), 125.4(5), 125.3(8), 124.9, 124.8, 124.5, 124.0, 123.2, 123.1, 35.1(2), 35.0(7), 31.4(6), 31.3(8); HRMS (MALDI) calcd for $C_{74}H_{64}^{+}$ [M]$^{+}$: 952.50025, found: 952.50020.

2-(2,2-Diphenylvinyl)-biphenyl (1r)

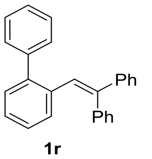

1r

Colourless solid; 648.3 mg, isolated yield 65%; ^{1}H NMR (400 MHz, $CDCl_3$) δ 7.45–7.43 (m, 2H), 7.37 (t, *J* = 7.2 Hz, 2H), 7.32–7.15 (m, 13H), 7.05–6.99 (m, 2H), 6.80 (s, 1H); ^{13}C NMR (100 MHz, $CDCl_3$) δ 143.4, 142.5, 141.8, 141.2, 140.2, 135.8, 130.7, 130.5, 129.8, 129.5, 128.2, 128.1, 128.04, 127.99, 127.9, 127.3, 127.1, 126.9, 126.6; HRMS (APCI) calcd for $C_{26}H_{20}^{+}$ [M]$^{+}$: 332.15595, found: 332.15602.

9,10-Diphenylphenanthrene (2r)

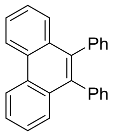

2r

Colourless solid; **Condition A**: 61.5 mg, isolated yield 93%; **Condition B**: 53.5 mg, isolated yield 81%; ^{1}H NMR (400 MHz, $CDCl_3$) δ 8.83 (d, *J* = 8.4 Hz, 2H), 7.68 (dd, *J* = 8.0, 7.2 Hz, 2H), 7.59 (d, *J* = 8.4 Hz, 2H), 7.51 (dd, *J* = 6.8, 6.8 Hz, 2H), 7.28–7.17 (m, 10H); ^{13}C NMR (100 MHz, $CDCl_3$) δ 139.5, 137.2, 131.9, 131.0, 130.0, 127.8, 127.6, 126.6, 126.5, 126.4, 122.5; HRMS (APCI) calcd for $C_{26}H_{18}^{+}$ $[M]^{+}$: 330.14030, found: 330.14034.

10'*H*-spiro[fluorene-9,9'-phenanthrene] (3a)

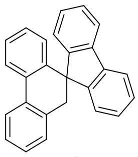

3a

Colourless solid; ^{1}H NMR (400 MHz, $CDCl_3$) δ 7.97 (dd, *J* = 8.0, 3.2 Hz, 2H), 7.78 (d, *J* = 8.0 Hz, 2H), 7.46 (dd, *J* = 8.0, 7.2 Hz, 1H), 7.38–7.28 (m, 4H), 7.17–7.12 (m, 3H), 7.06–7.01 (m, 3H), 6.63 (d, *J* = 8.0 Hz, 1H), 3.32 (s, 2H); ^{13}C NMR (100 MHz, $CDCl_3$) δ 150.9, 140.5, 139.8, 135.2, 134.9, 134.3, 129.1, 128.0, 127.7 (×2), 127.63, 127.57, 127.5, 126.5, 124.4, 124.2, 123.6, 120.0, 54.5, 41.4; HRMS (APCI) calcd for $C_{26}H_{18}^{+}$ $[M]^{+}$: 330.14030, found: 330.14036.

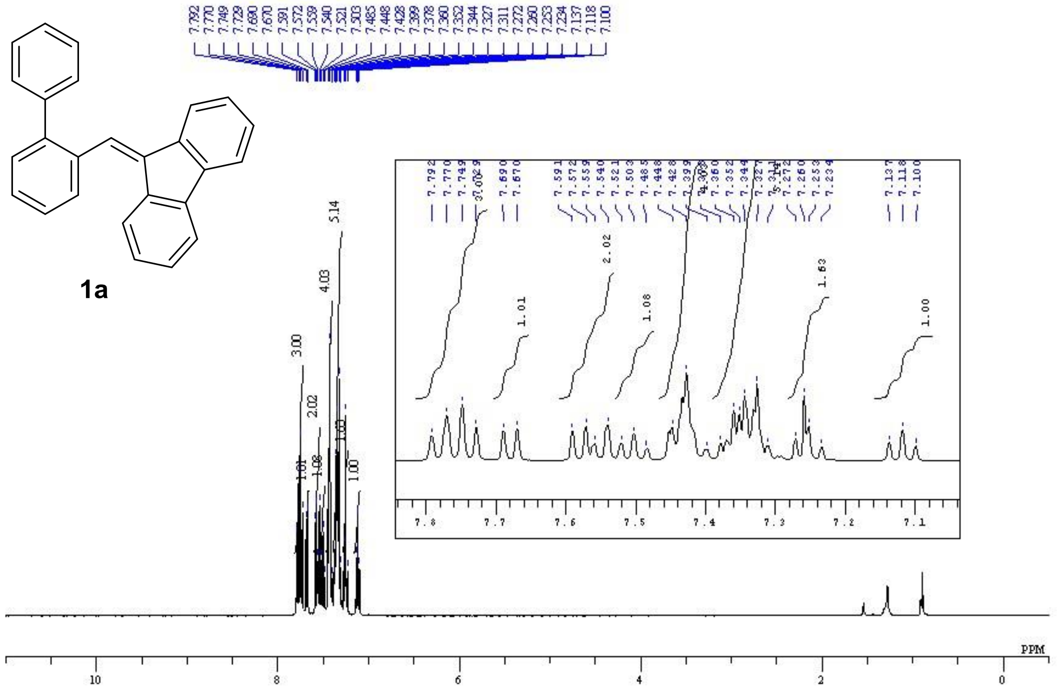

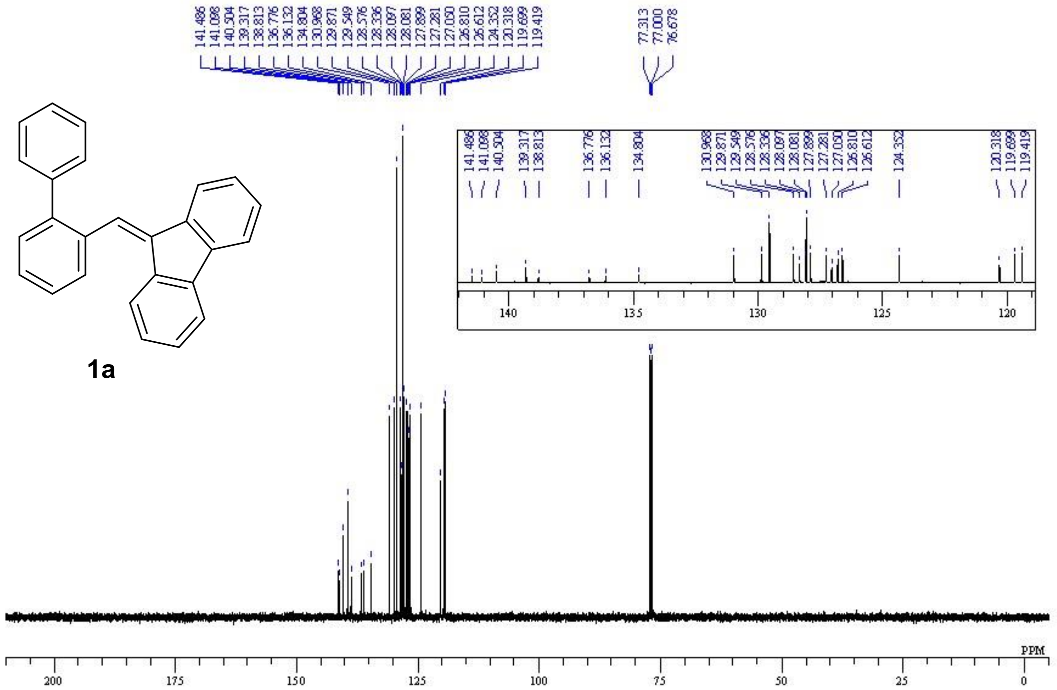

Supplementary Figure 4. ^{1}H and ^{13}C NMR spectra of compound **1a**

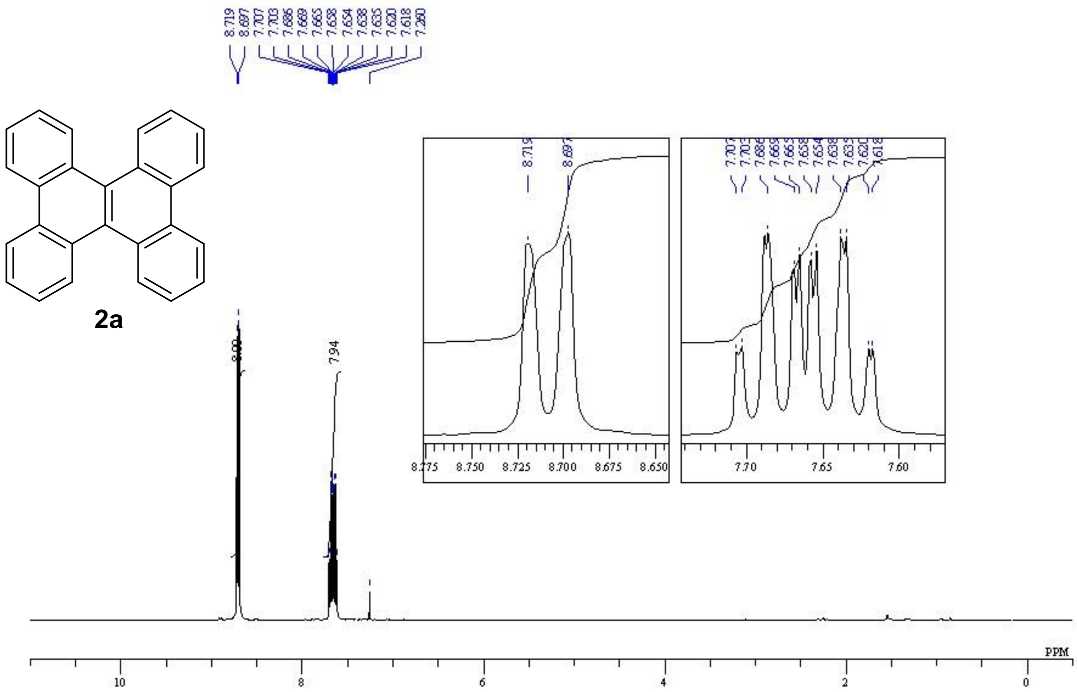

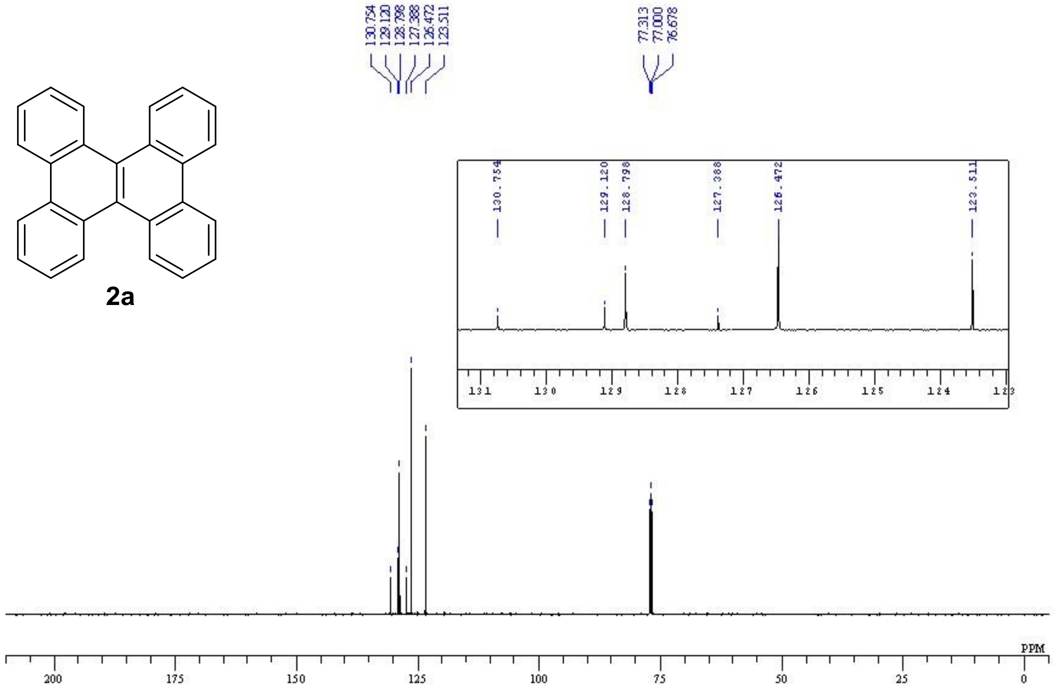

Supplementary Figure 5. ^{1}H and ^{13}C NMR spectra of compound **2a**

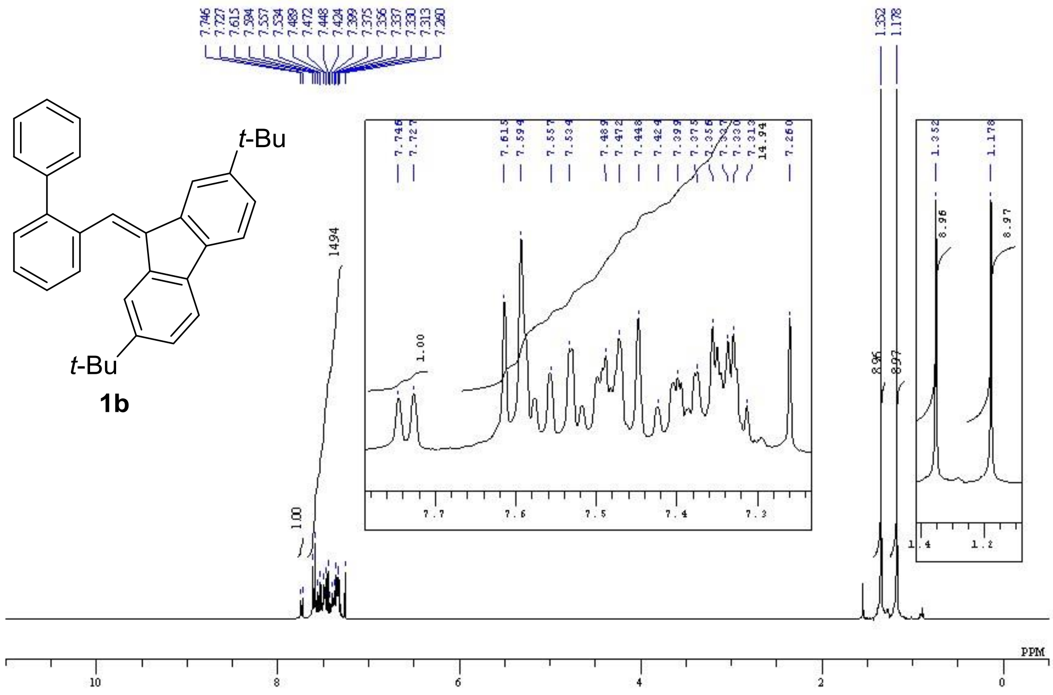

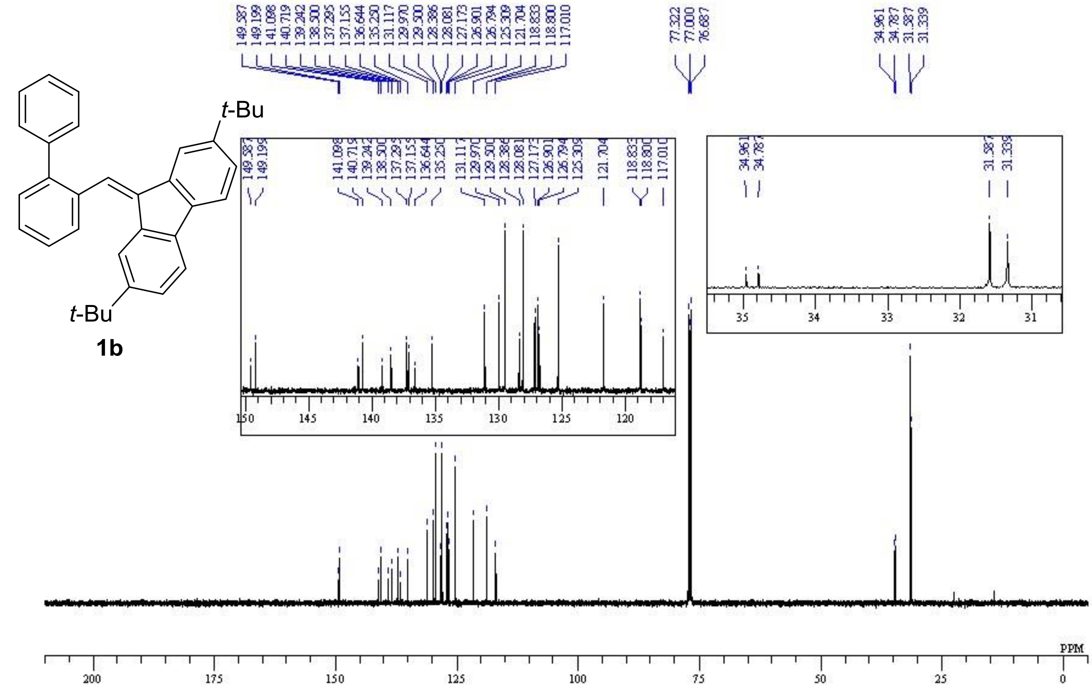

Supplementary Figure 6. ^{1}H and ^{13}C NMR spectra of compound **1b**

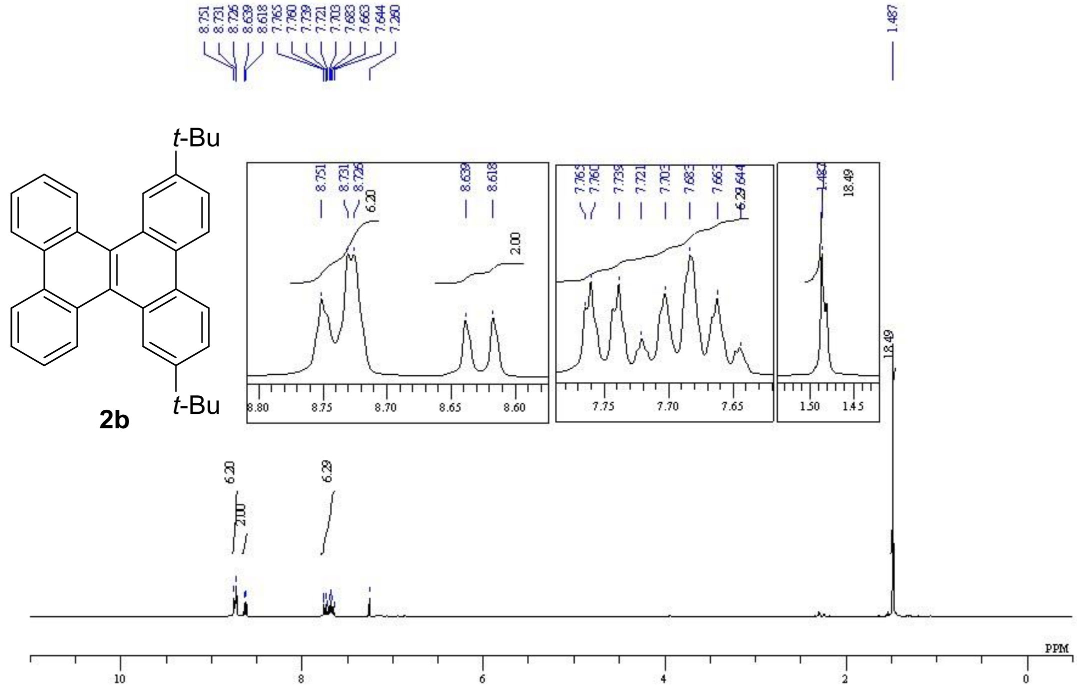

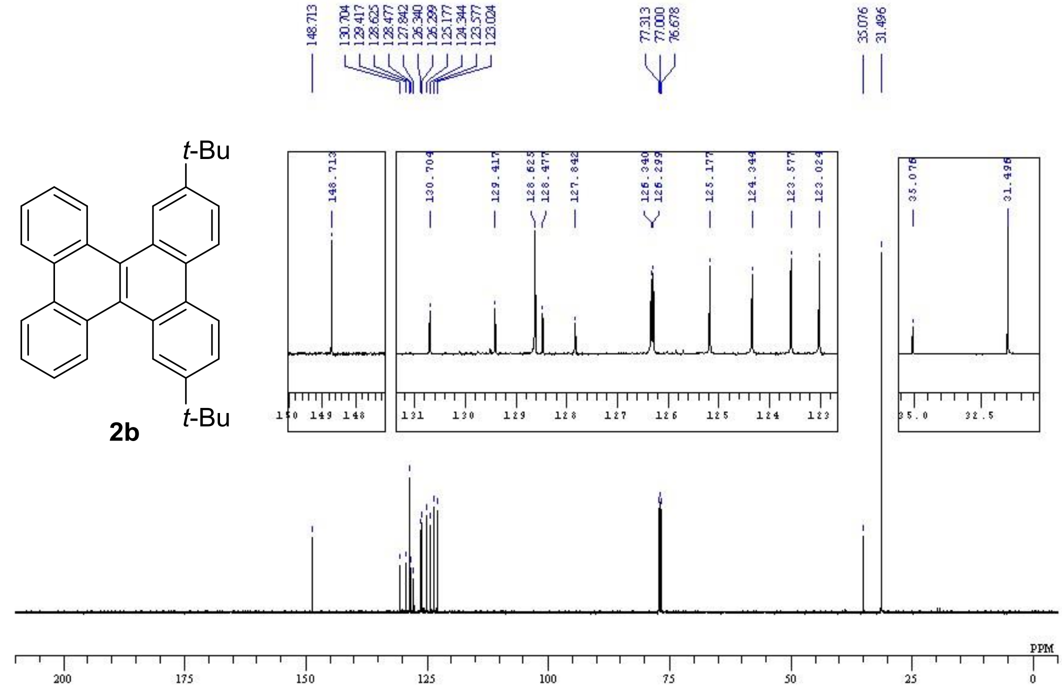

Supplementary Figure 7. ^{1}H and ^{13}C NMR spectra of compound **2b**

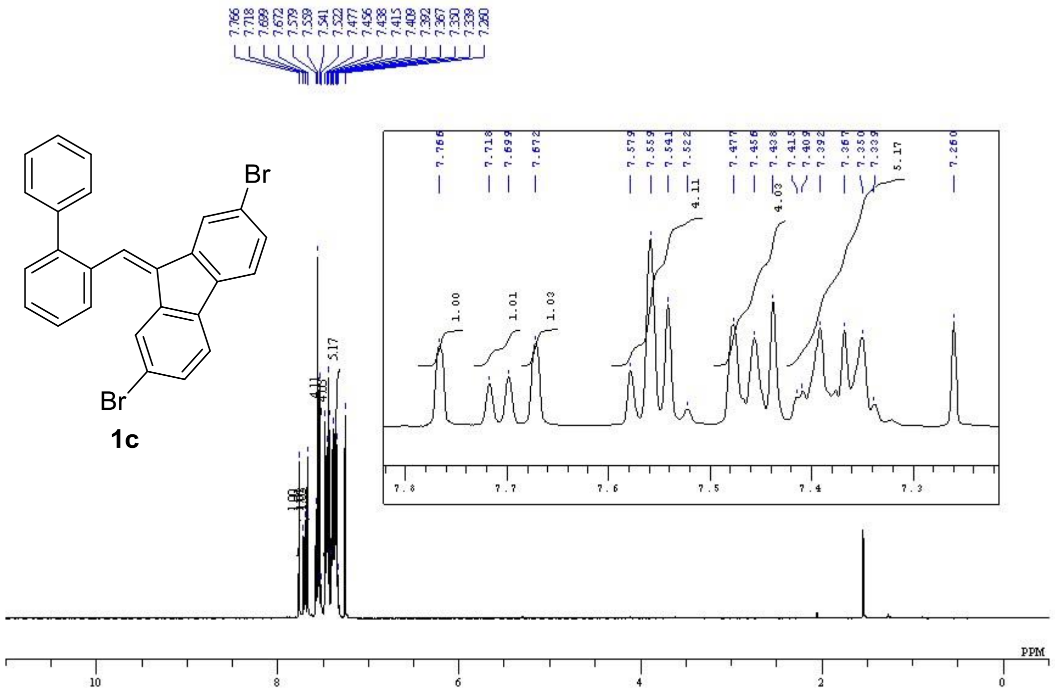

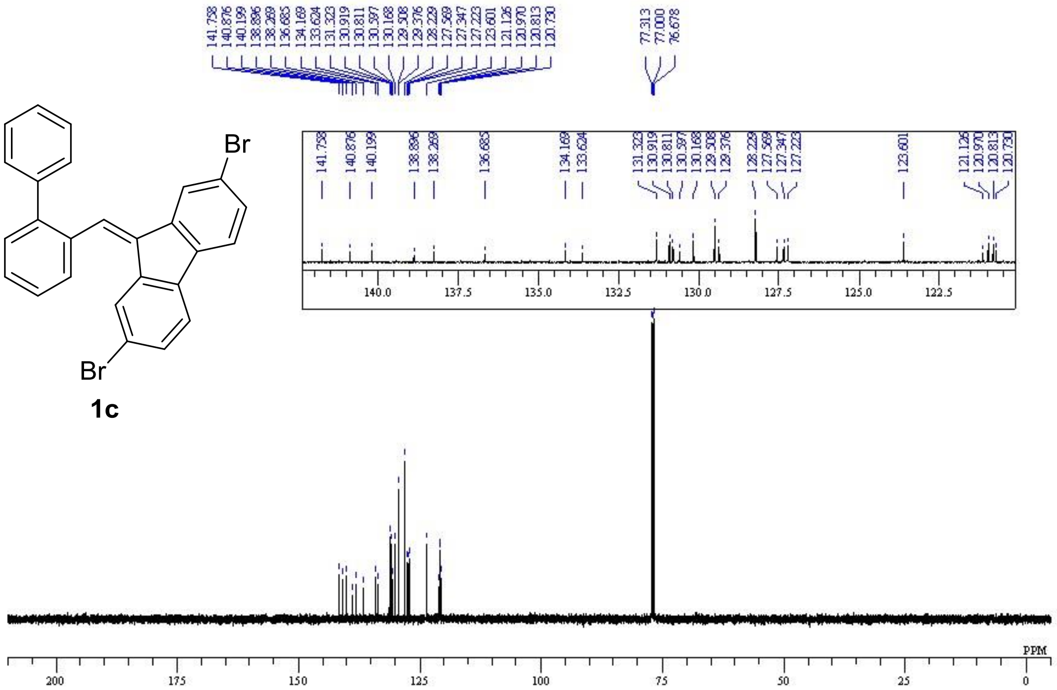

Supplementary Figure 8. ^{1}H and ^{13}C NMR spectra of compound **1c**

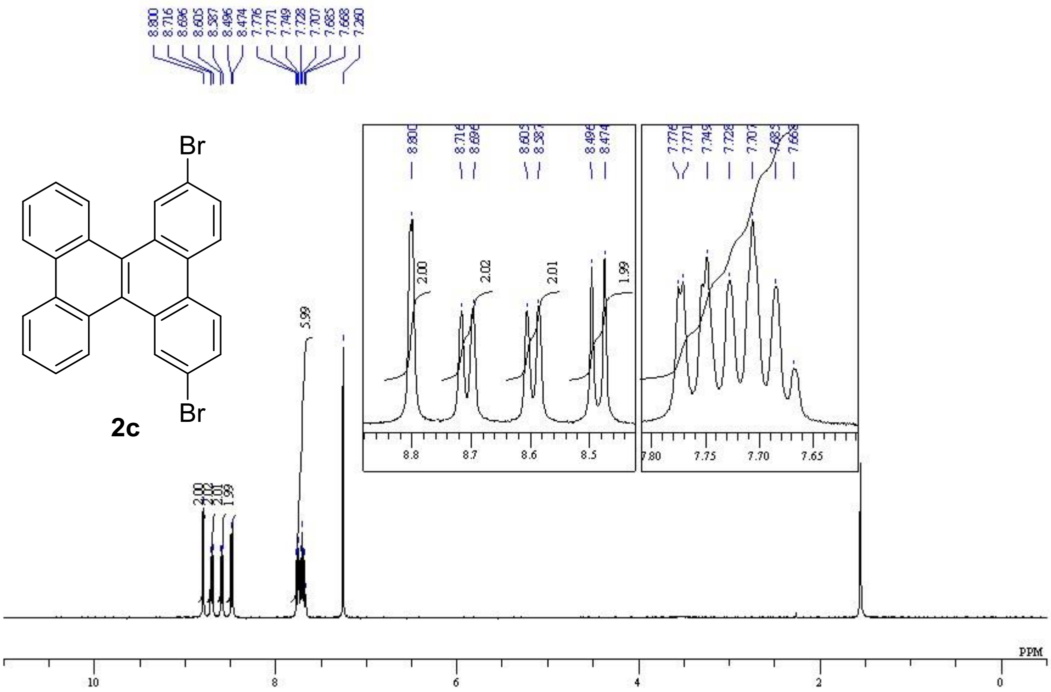

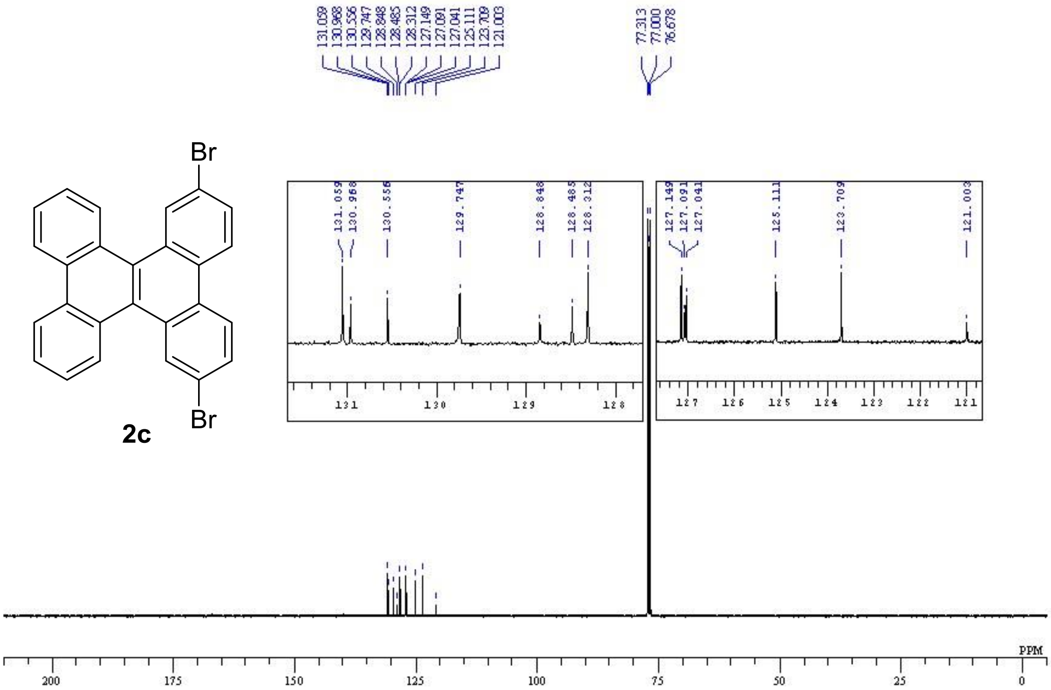

Supplementary Figure 9. ^{1}H and ^{13}C NMR spectra of compound **2c**

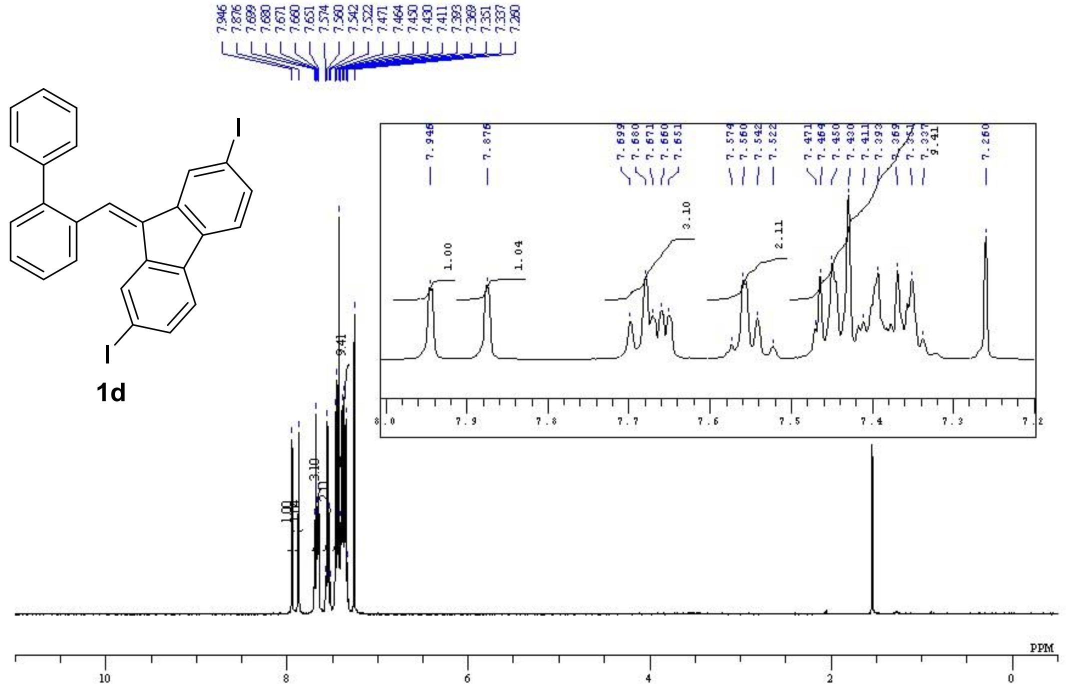

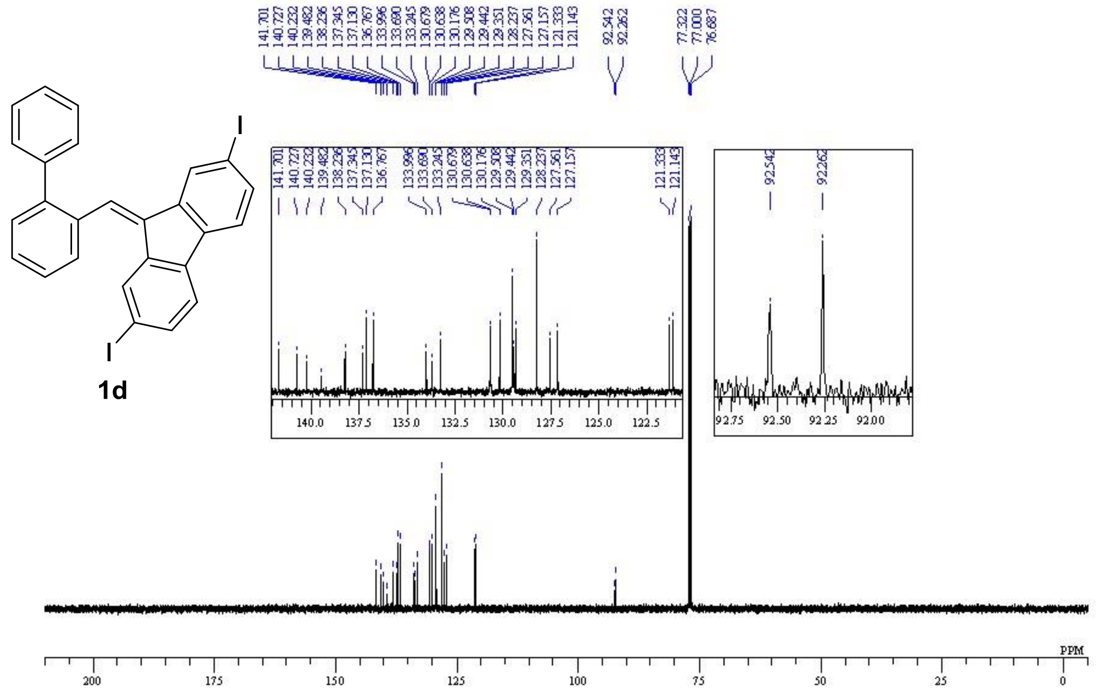

Supplementary Figure 10. ^{1}H and ^{13}C NMR spectra of compound **1d**

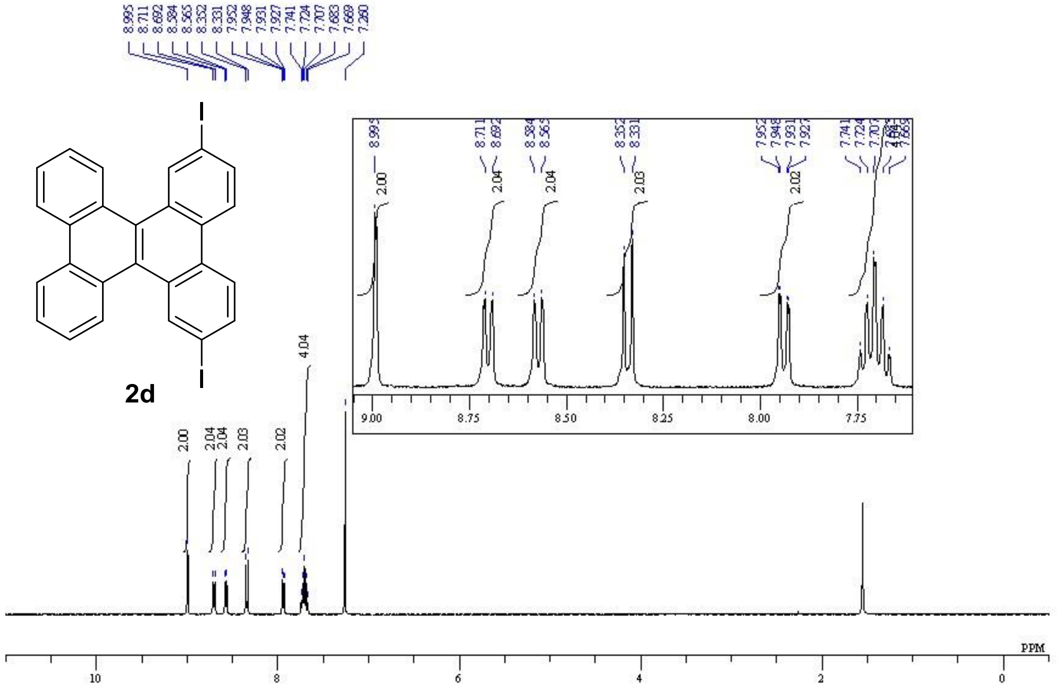

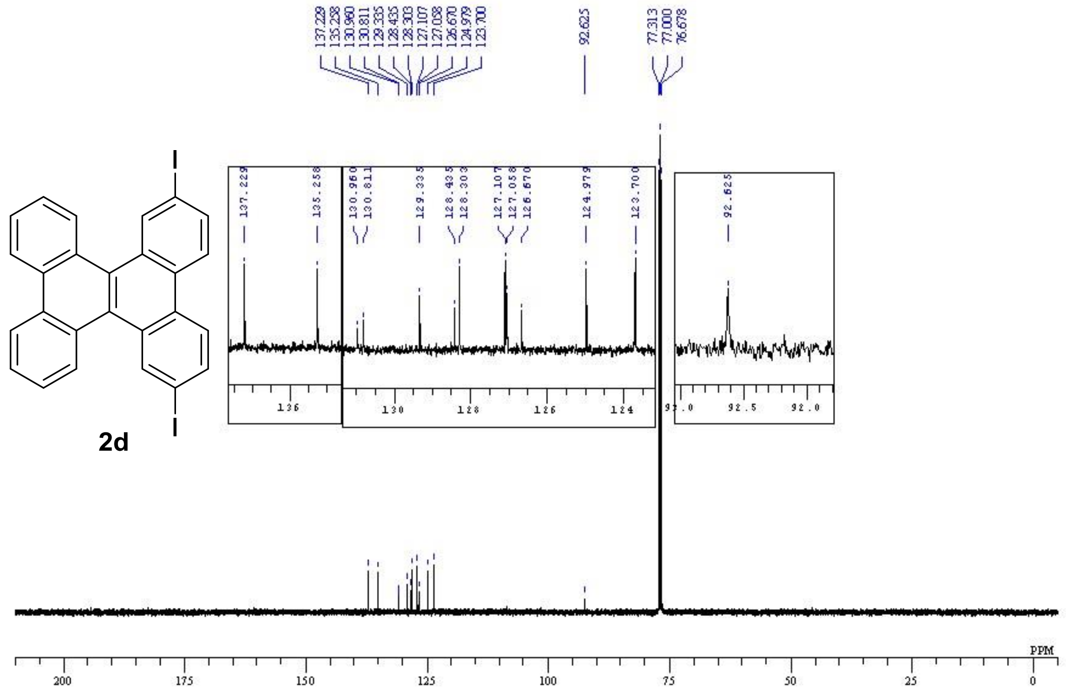

Supplementary Figure 11. ^{1}H and ^{13}C NMR spectra of compound **2d**

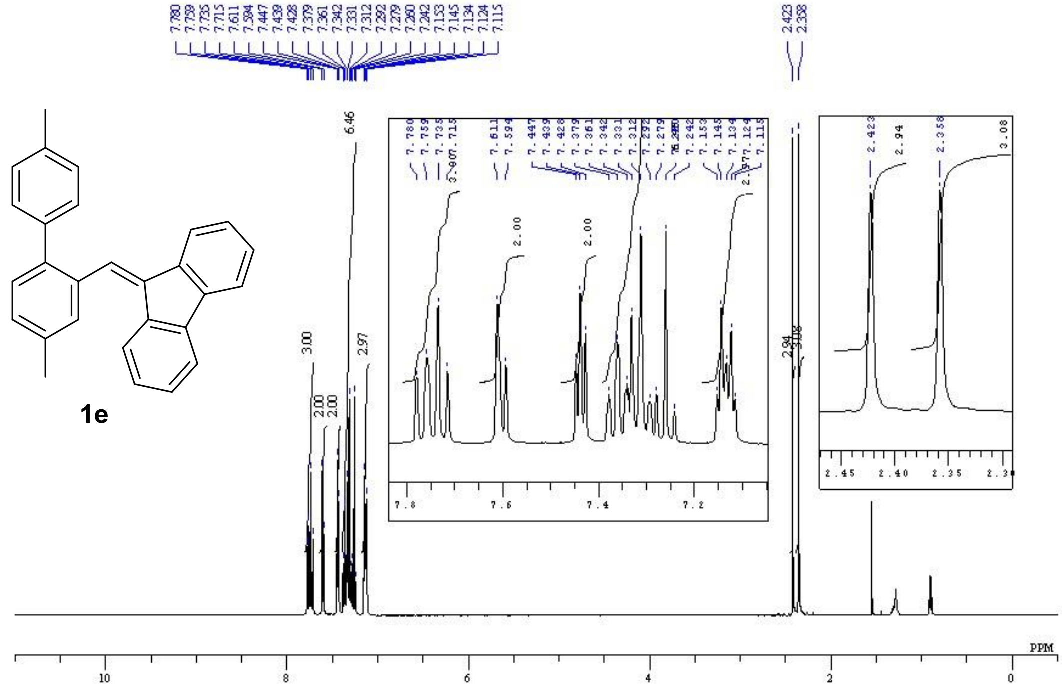

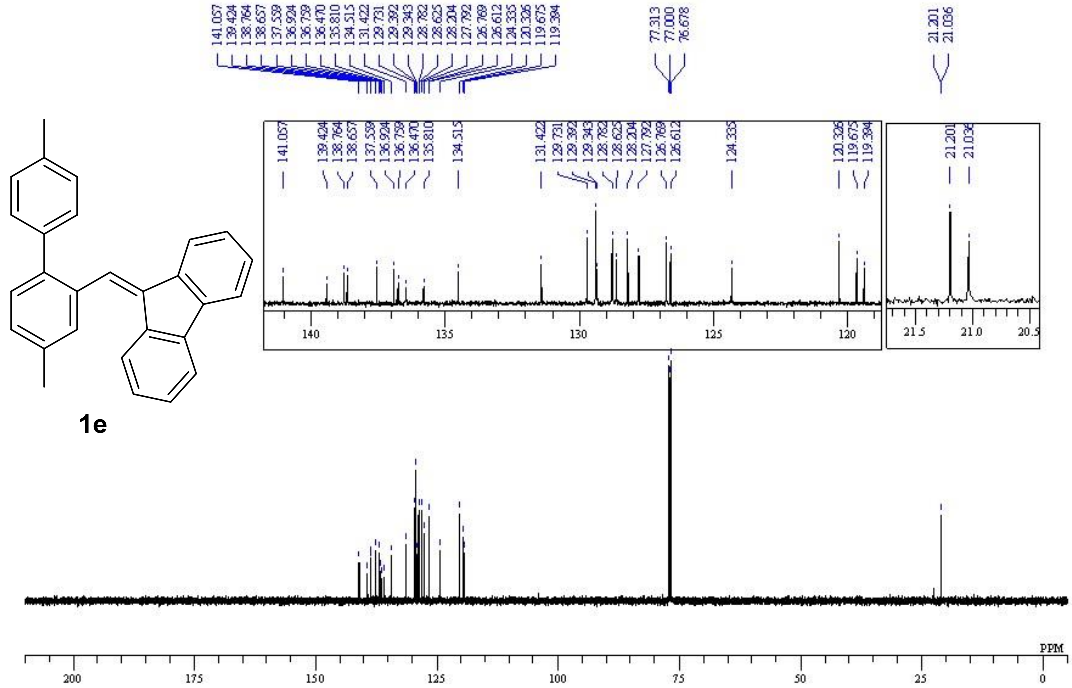

Supplementary Figure 12. ^{1}H and ^{13}C NMR spectra of compound **1e**

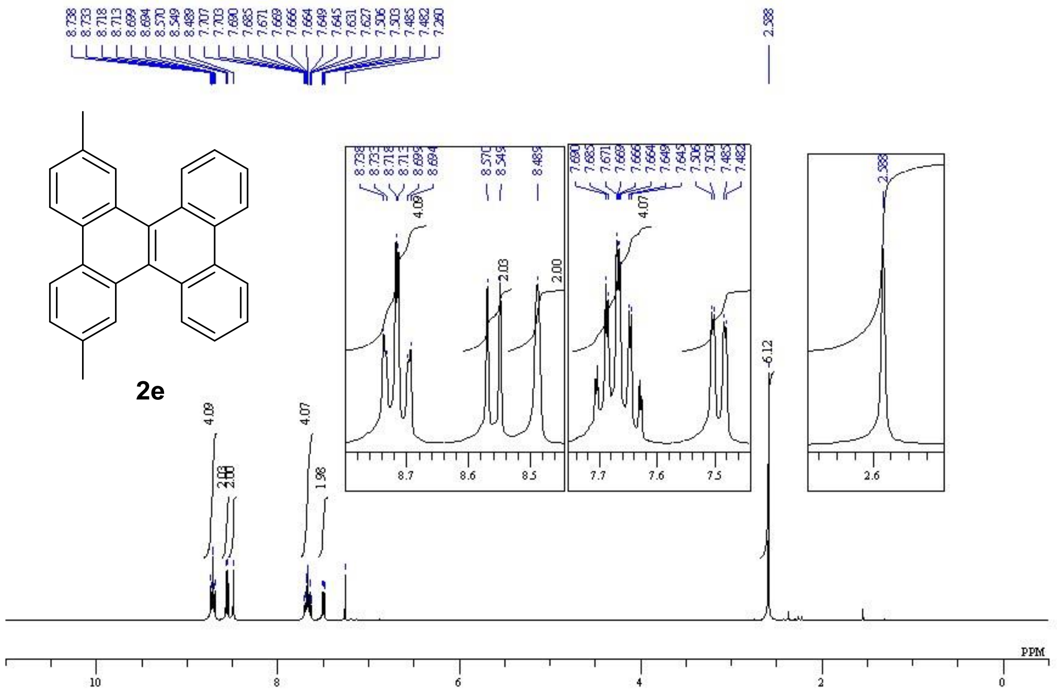

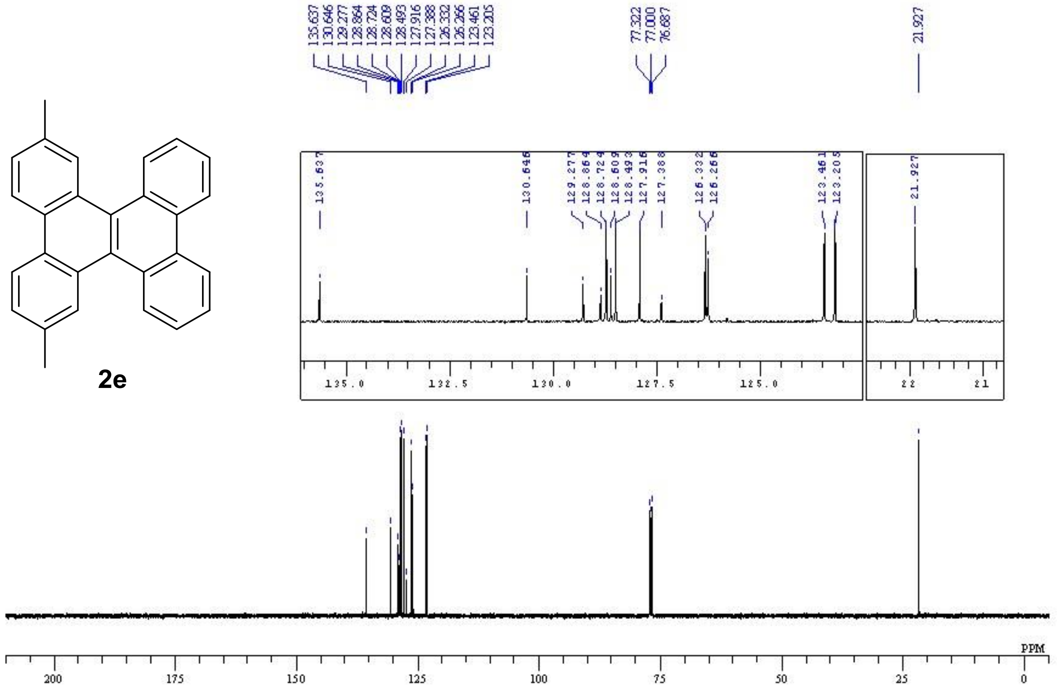

Supplementary Figure 13. ^{1}H and ^{13}C NMR spectra of compound **2e**

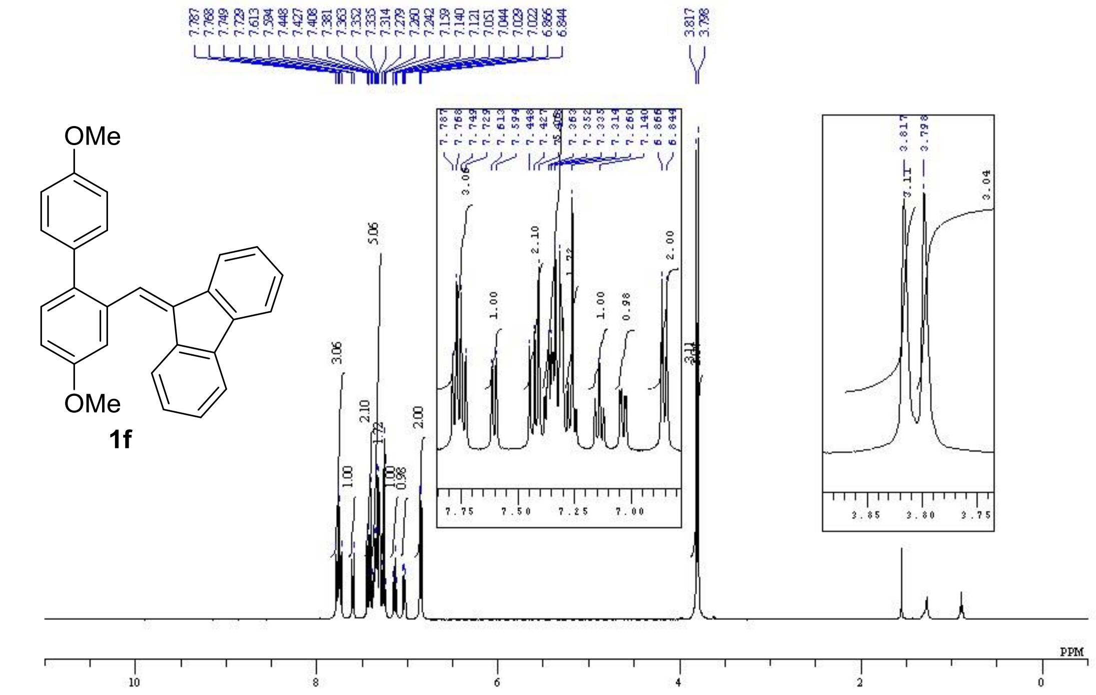

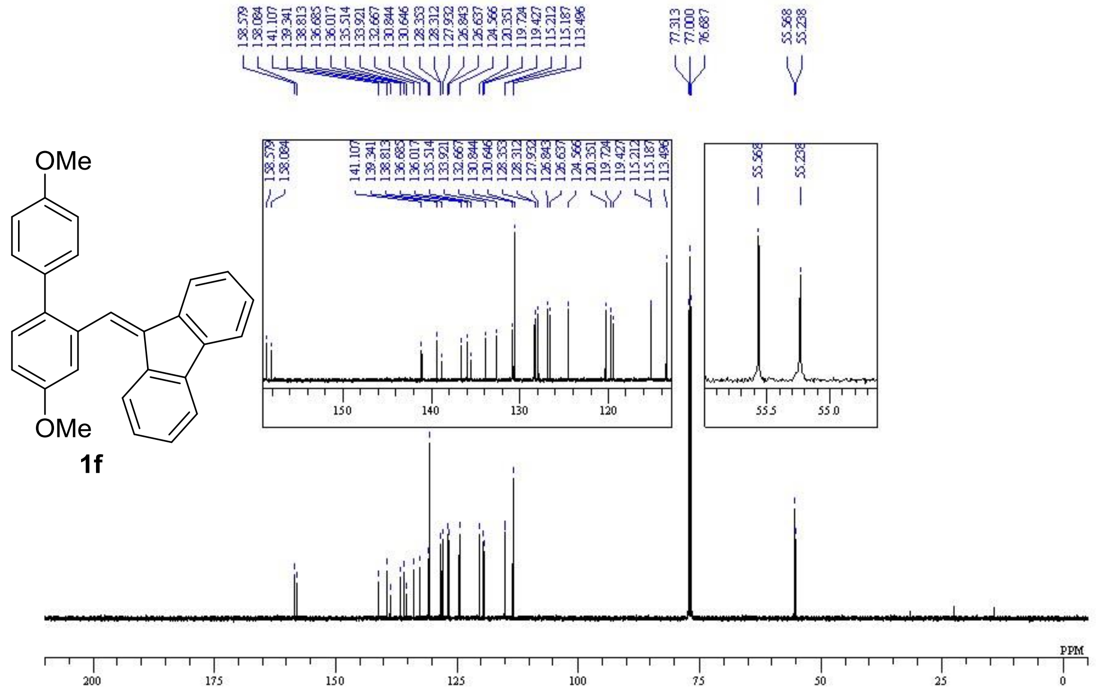

Supplementary Figure 14. ^{1}H and ^{13}C NMR spectra of compound **1f**

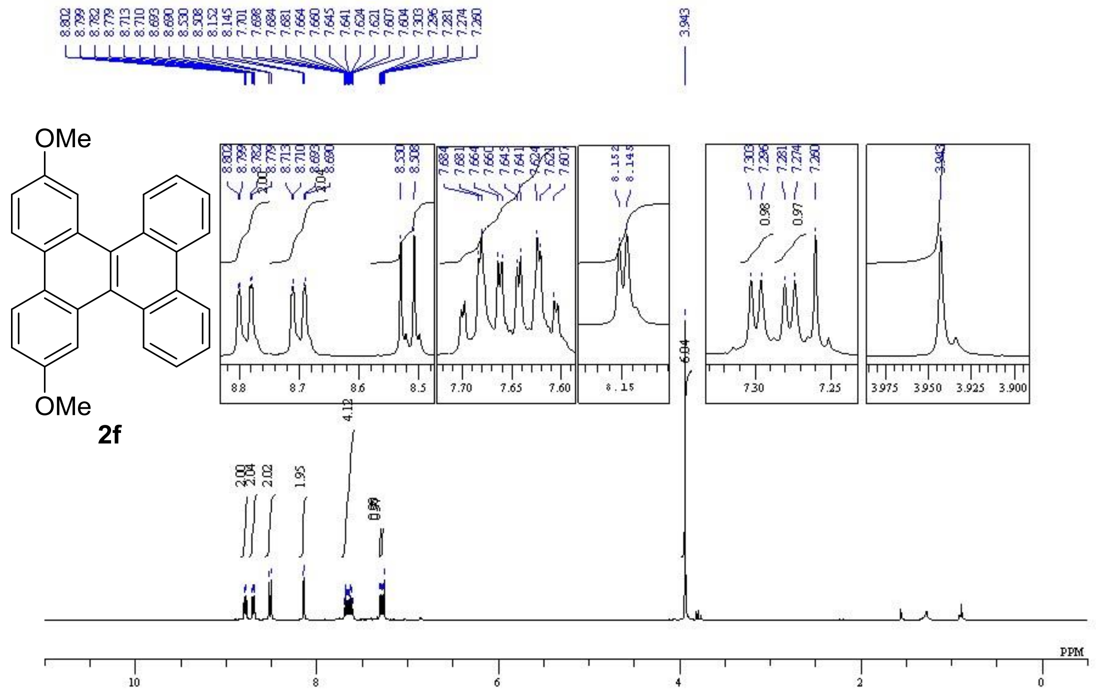

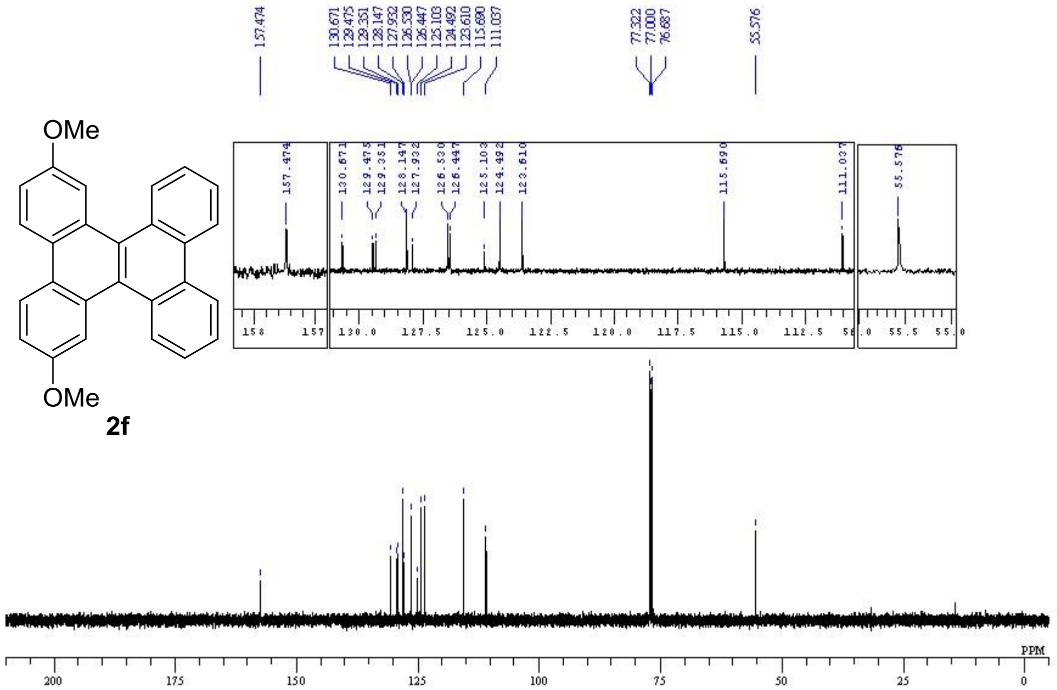

Supplementary Figure 15. ^{1}H and ^{13}C NMR spectra of compound **2f**

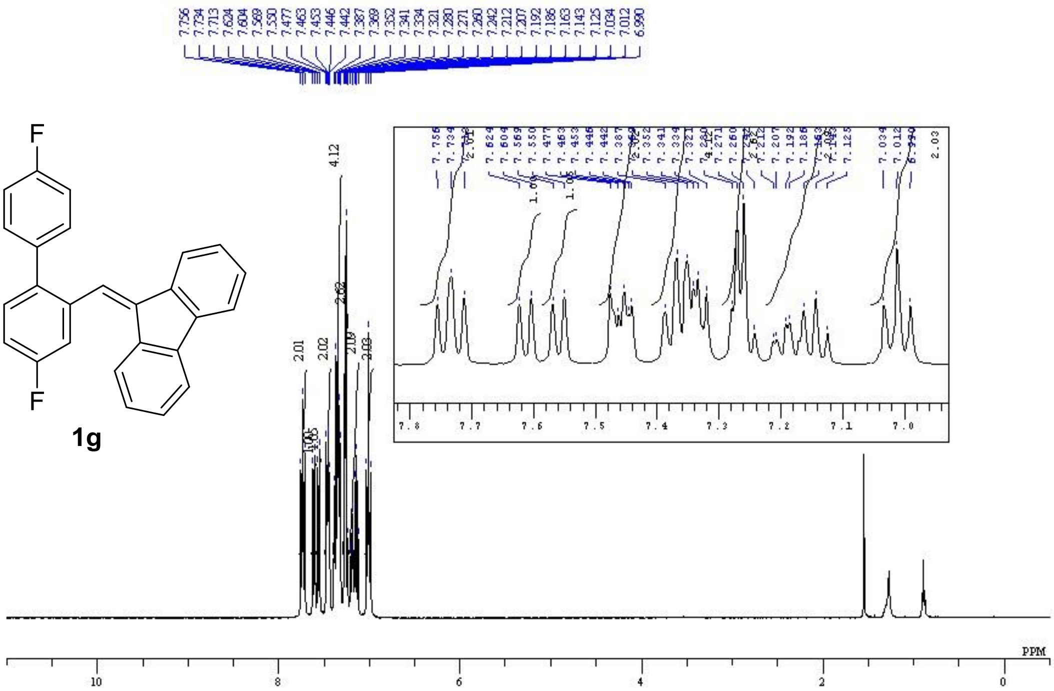

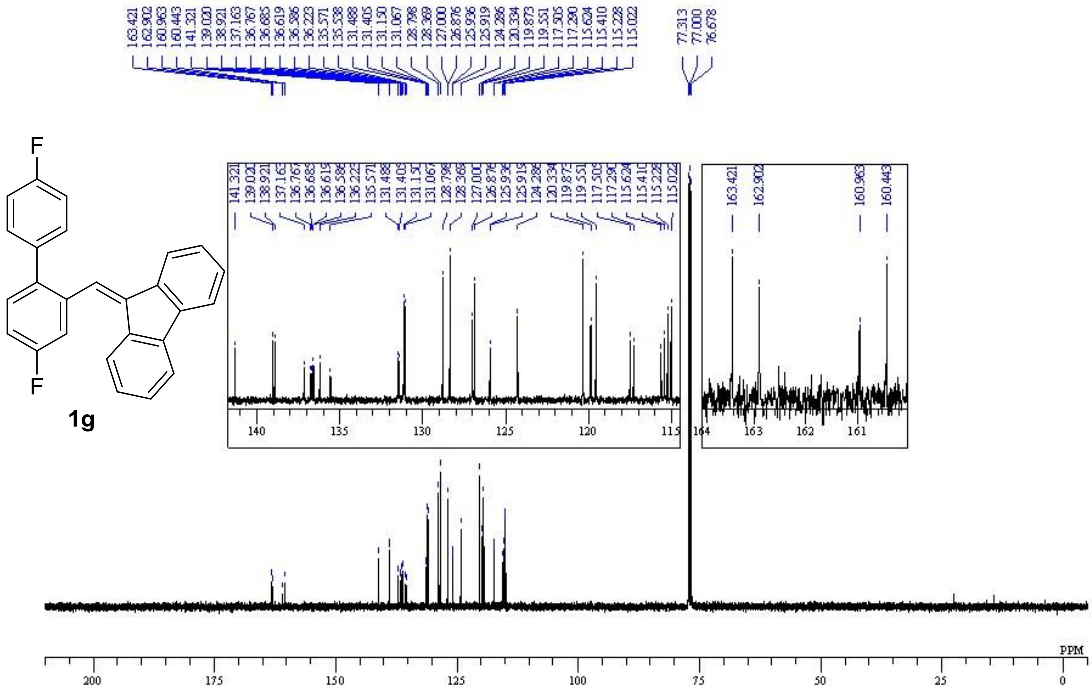

Supplementary Figure 16. ^{1}H and ^{13}C NMR spectra of compound **1g**

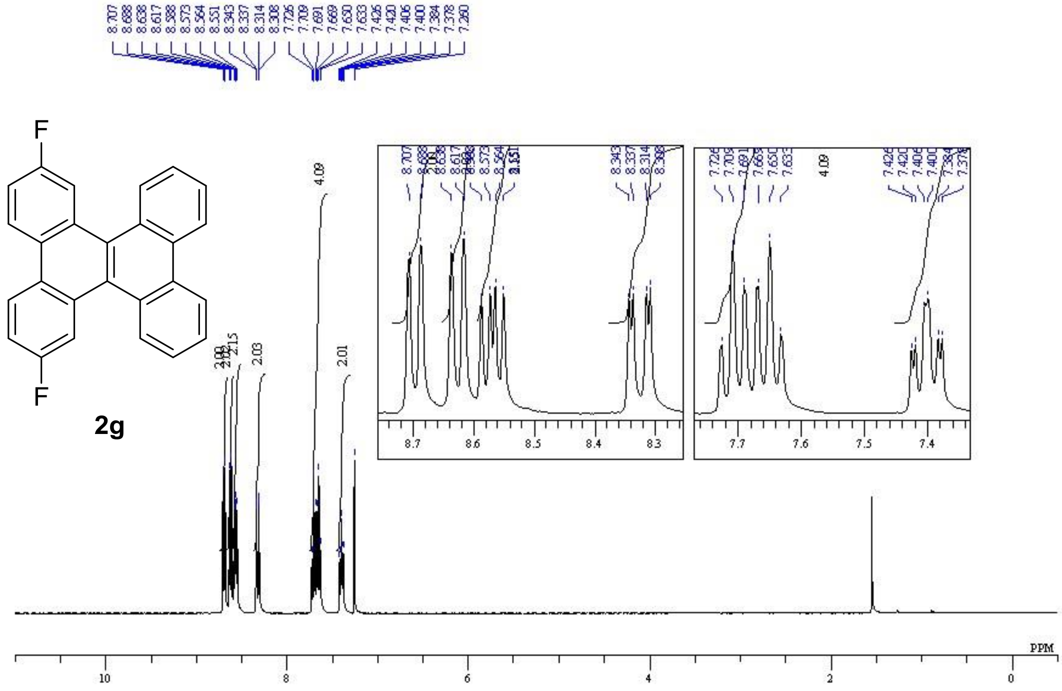

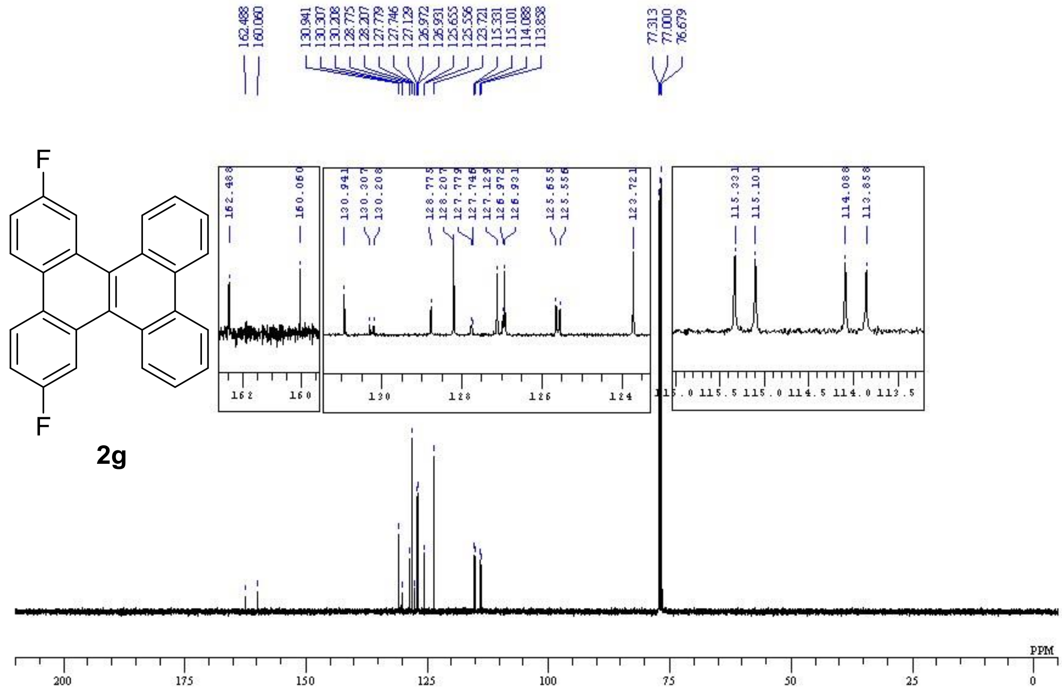

Supplementary Figure 17. ^{1}H and ^{13}C NMR spectra of compound **2g**

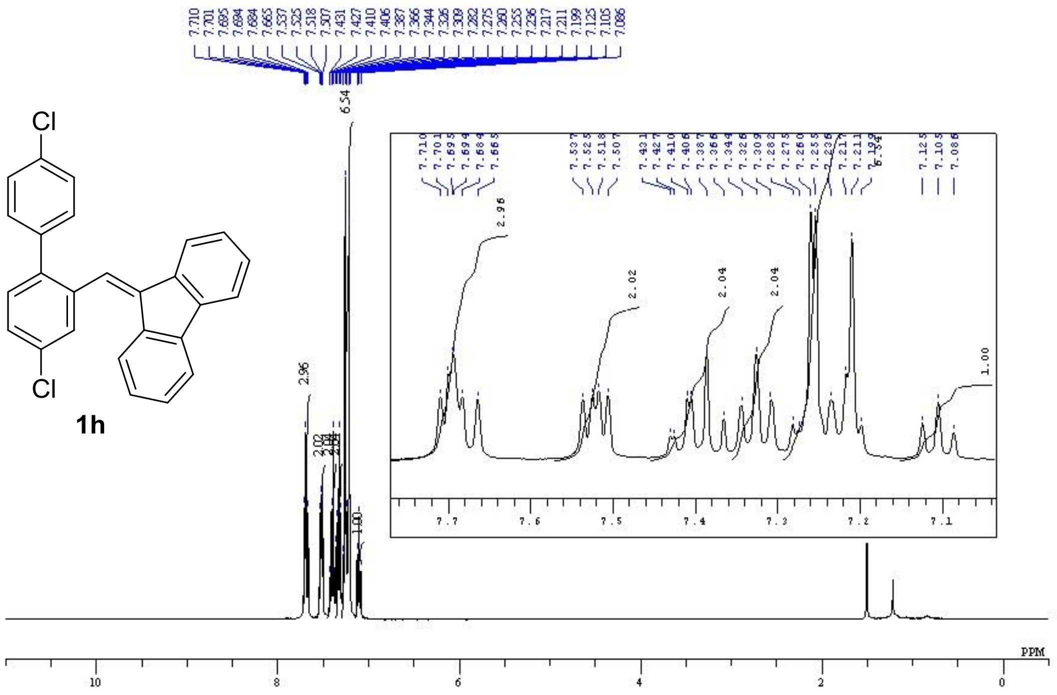

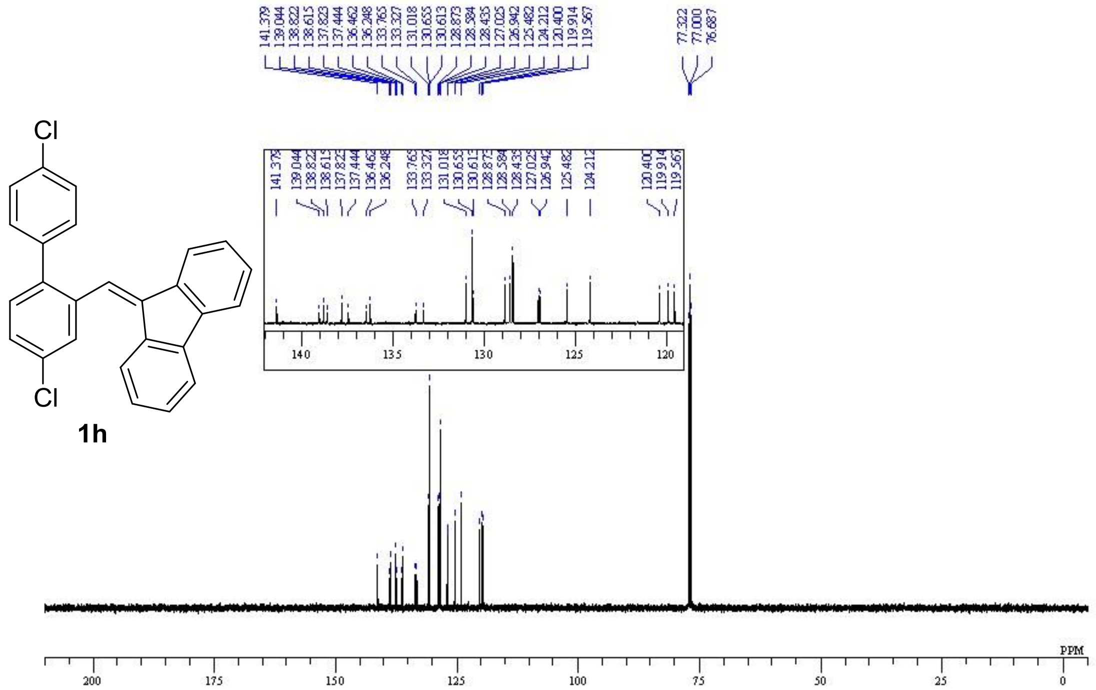

Supplementary Figure 18. ^{1}H and ^{13}C NMR spectra of compound **1h**

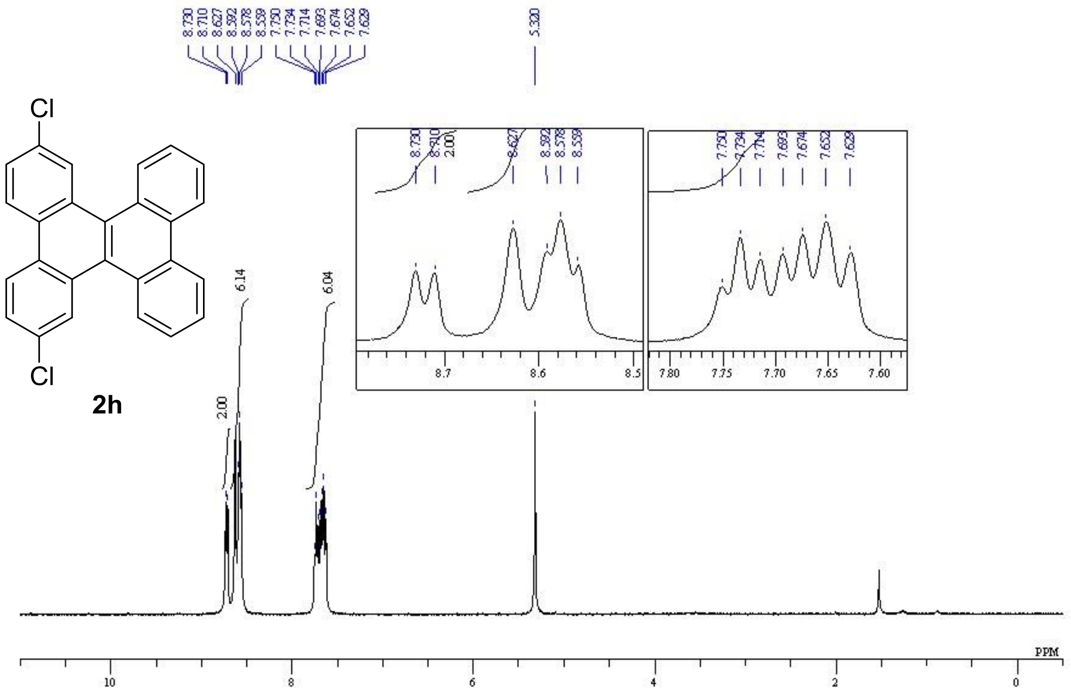

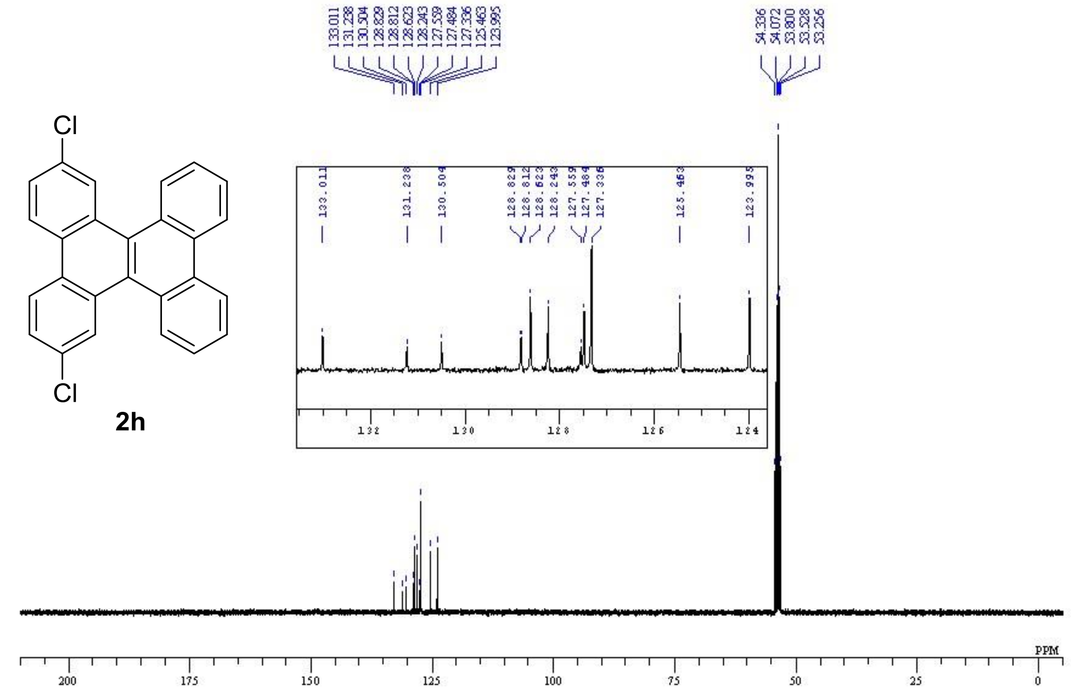

Supplementary Figure 19. ^{1}H and ^{13}C NMR spectra of compound **2h**

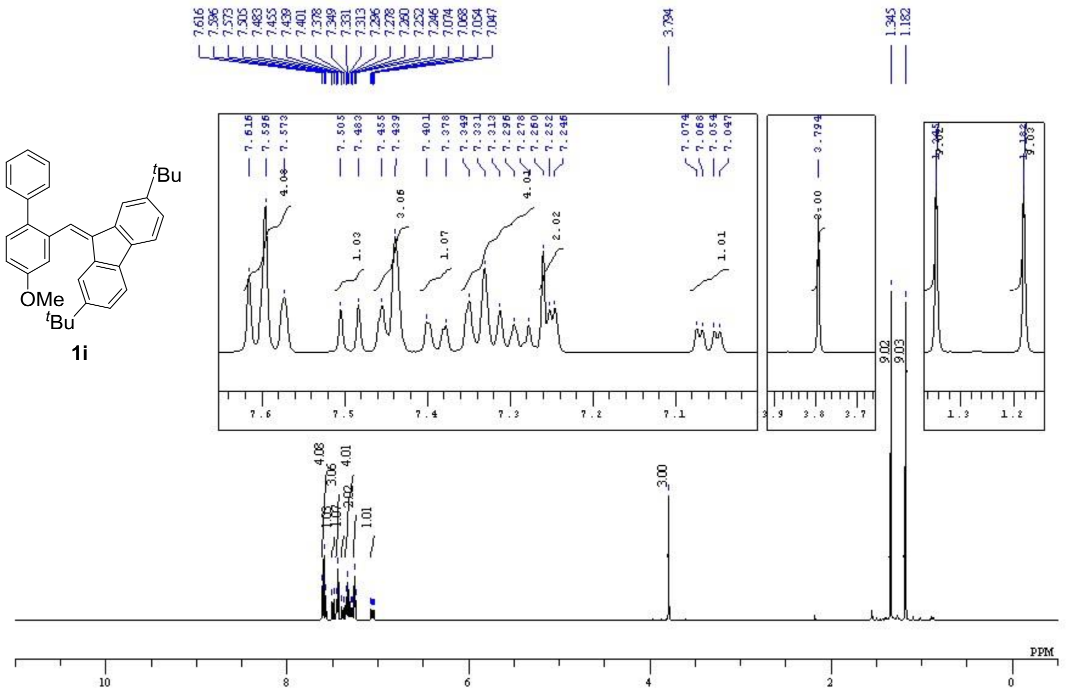

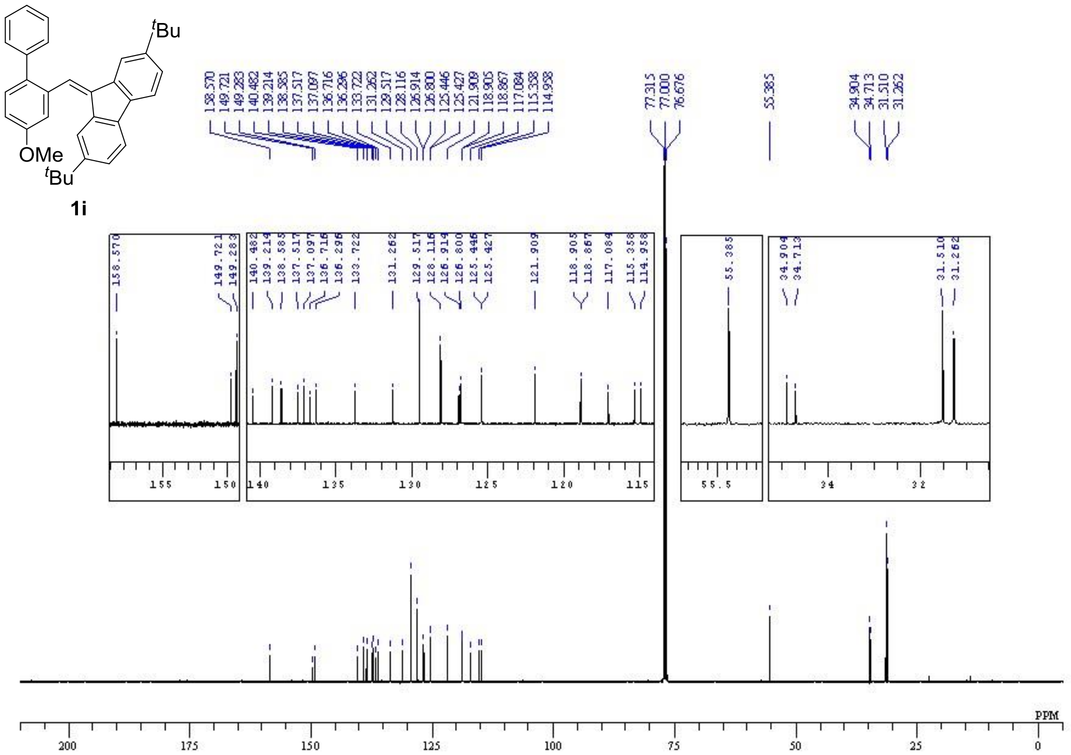

Supplementary Figure 20. ^{1}H and ^{13}C NMR spectra of compound **1i**

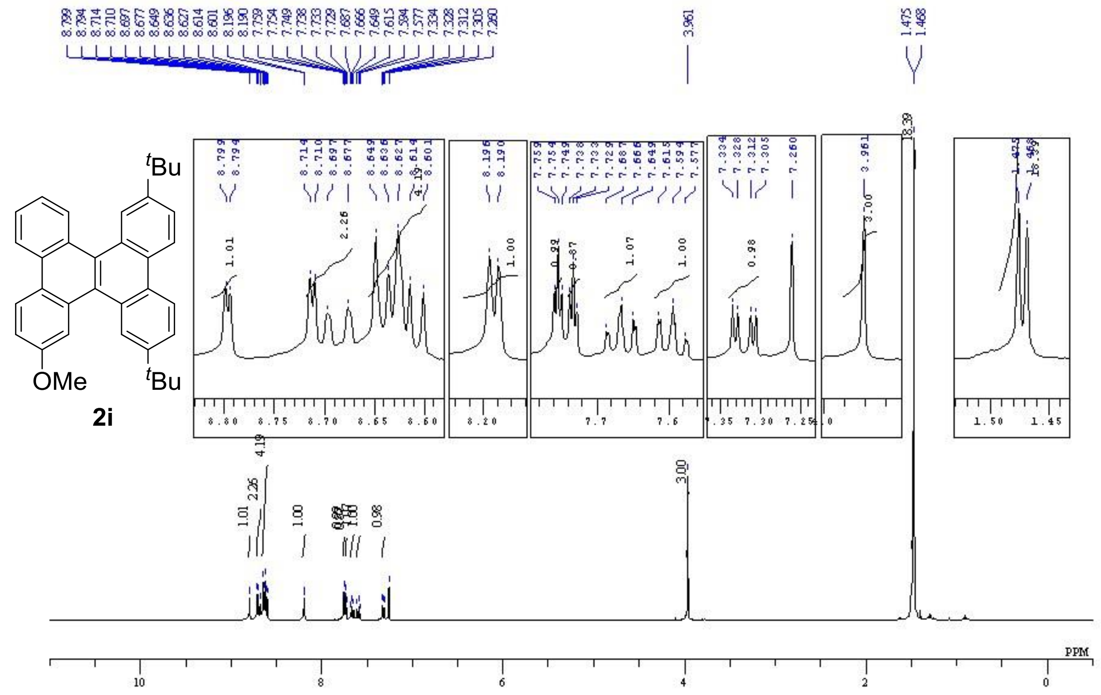

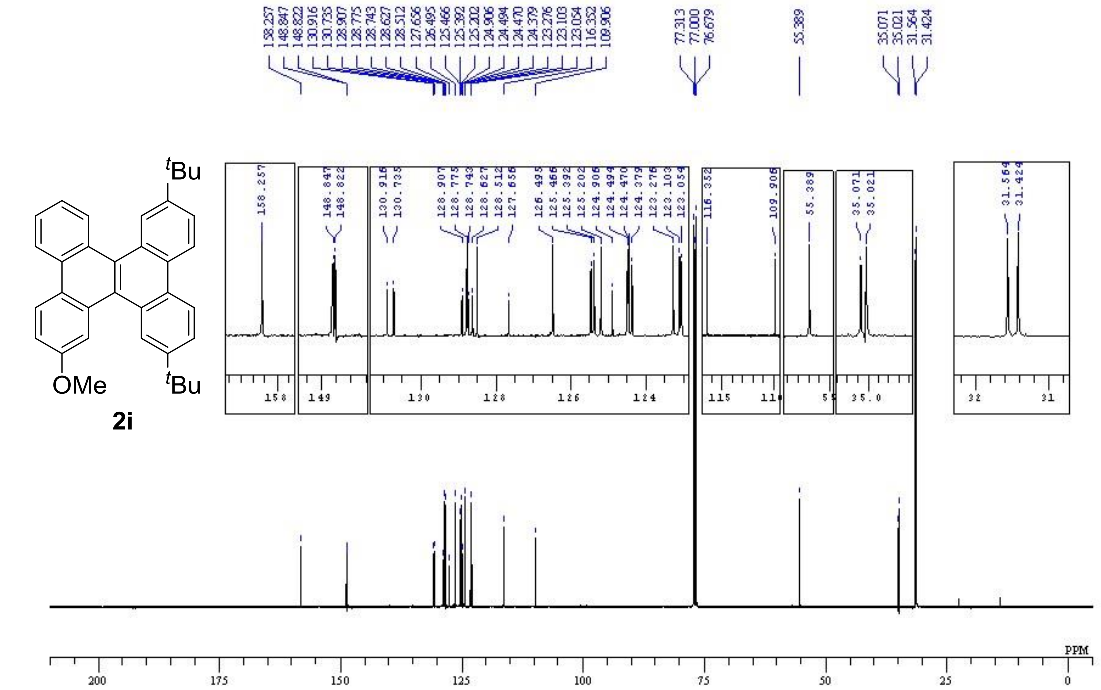

Supplementary Figure 21. ^{1}H and ^{13}C NMR spectra of compound **2i**

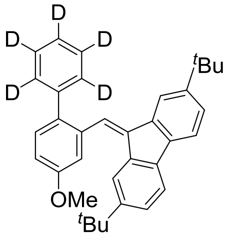

1i-d_5

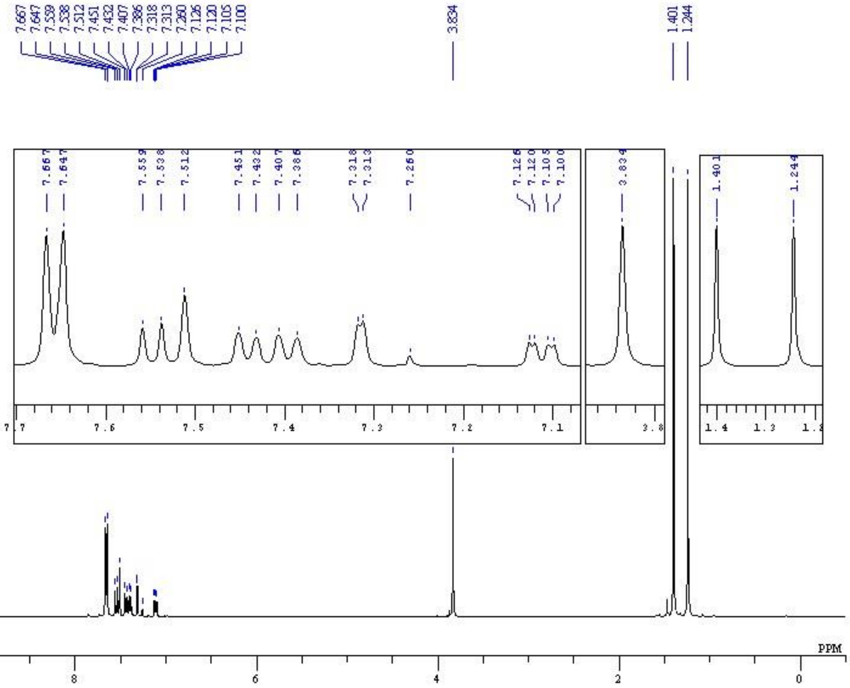

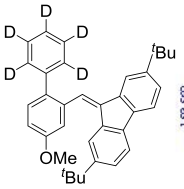

1i-d_5

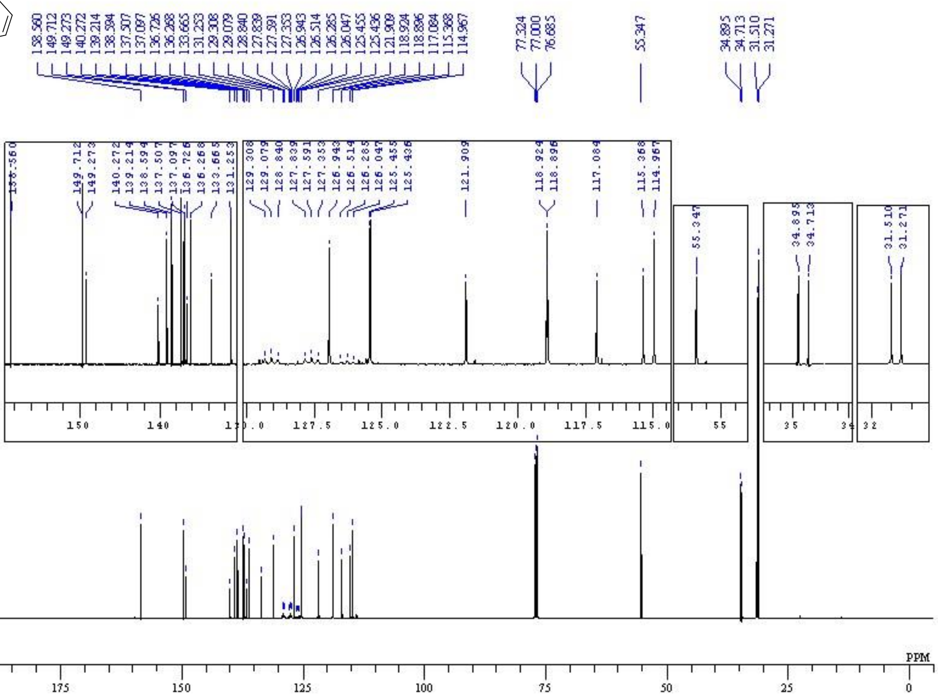

Supplementary Figure 22. ^{1}H and ^{13}C NMR spectra of compound **1i-d_5**

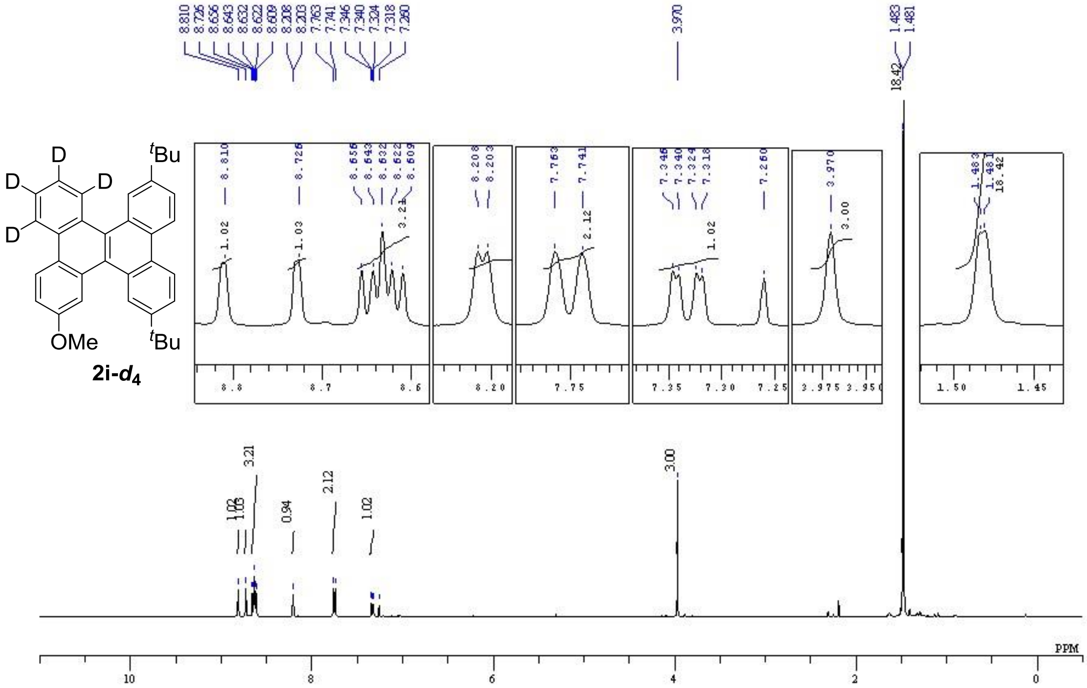

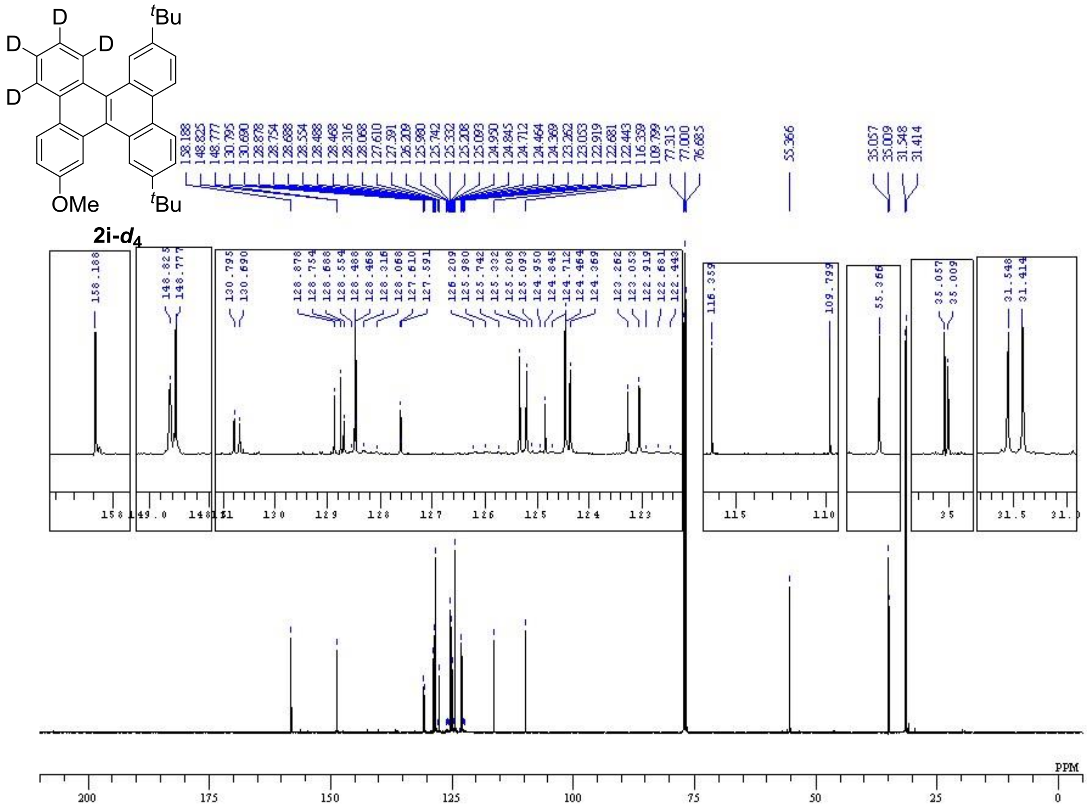

Supplementary Figure 23. ^{1}H and ^{13}C NMR spectra of compound **2i-*d*$_4$**

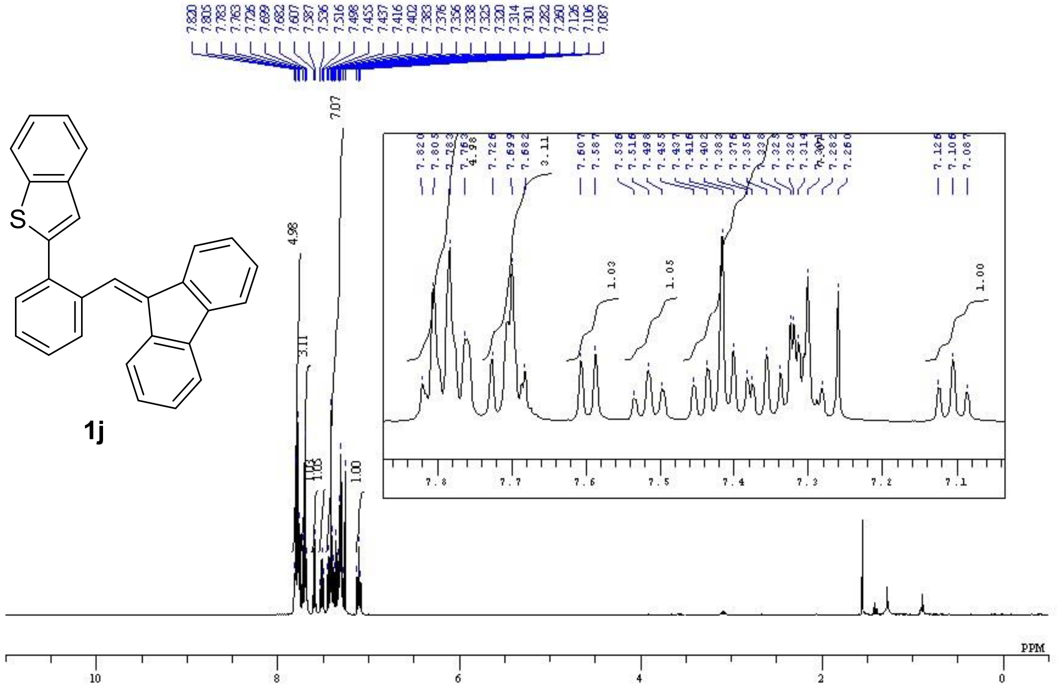

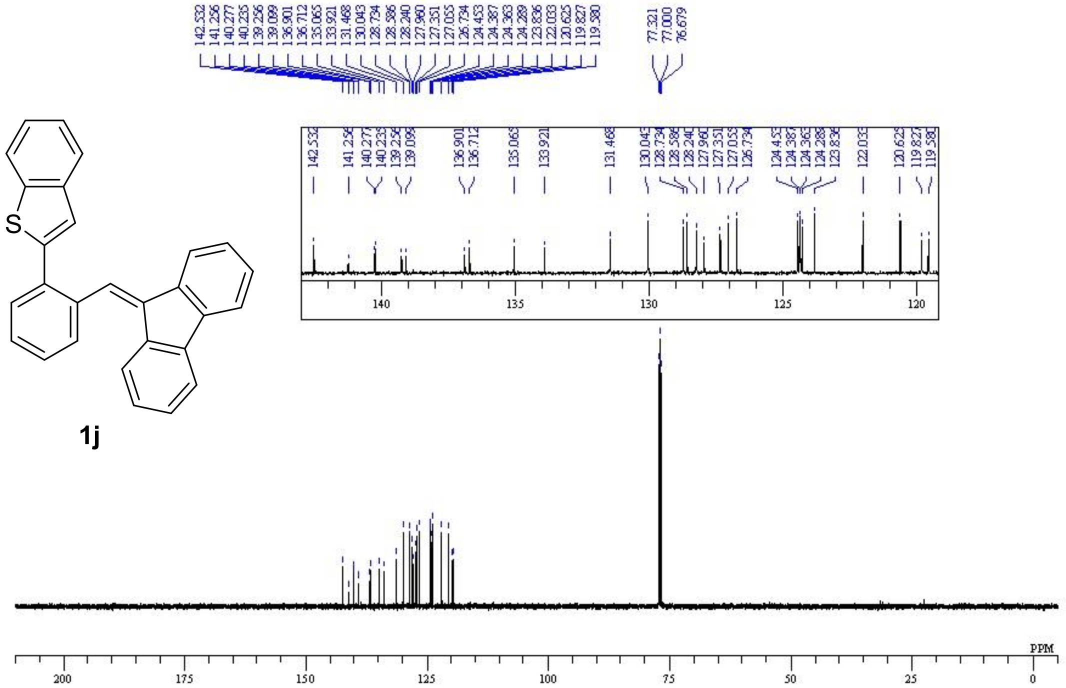

Supplementary Figure 24. ^{1}H and ^{13}C NMR spectra of compound **1j**

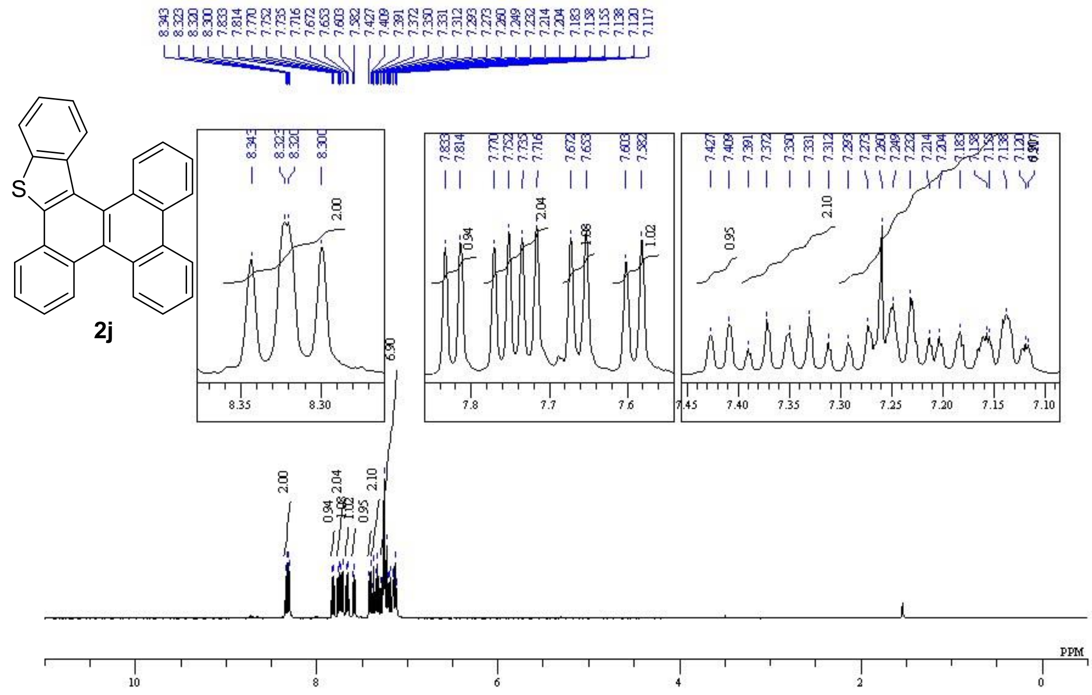

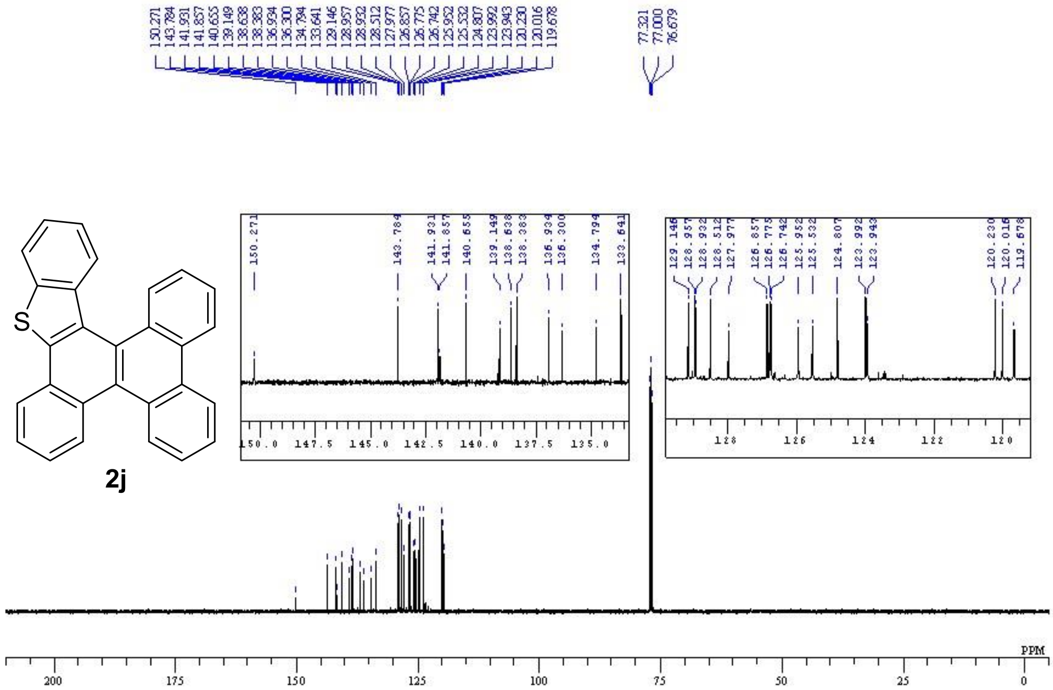

Supplementary Figure 25. ^{1}H and ^{13}C NMR spectra of compound **2j**

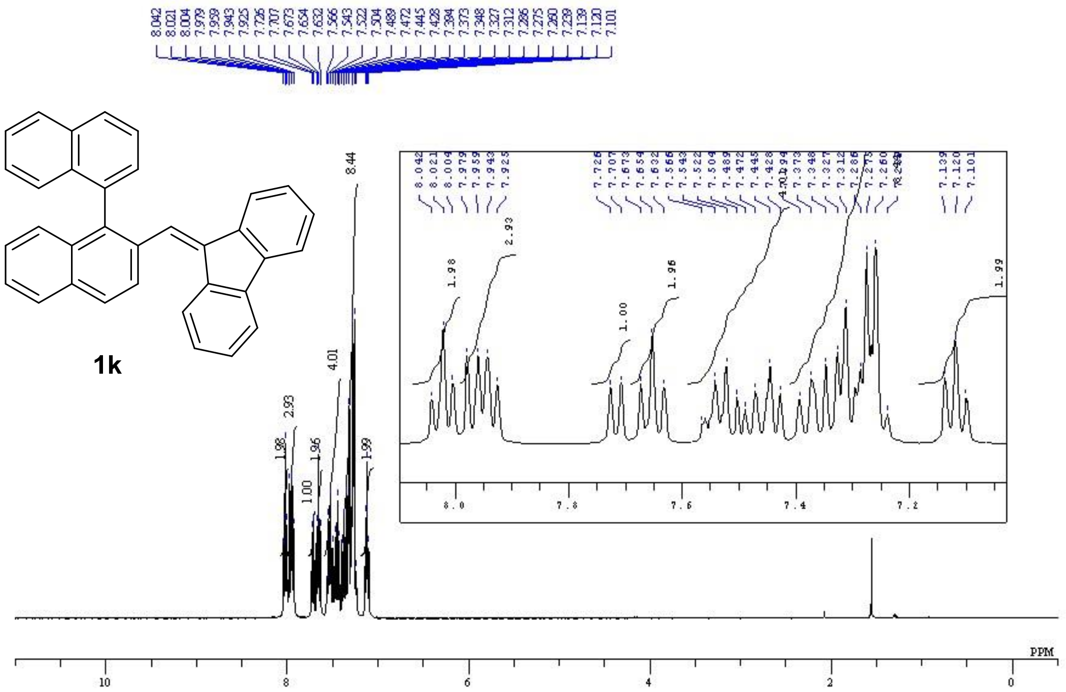

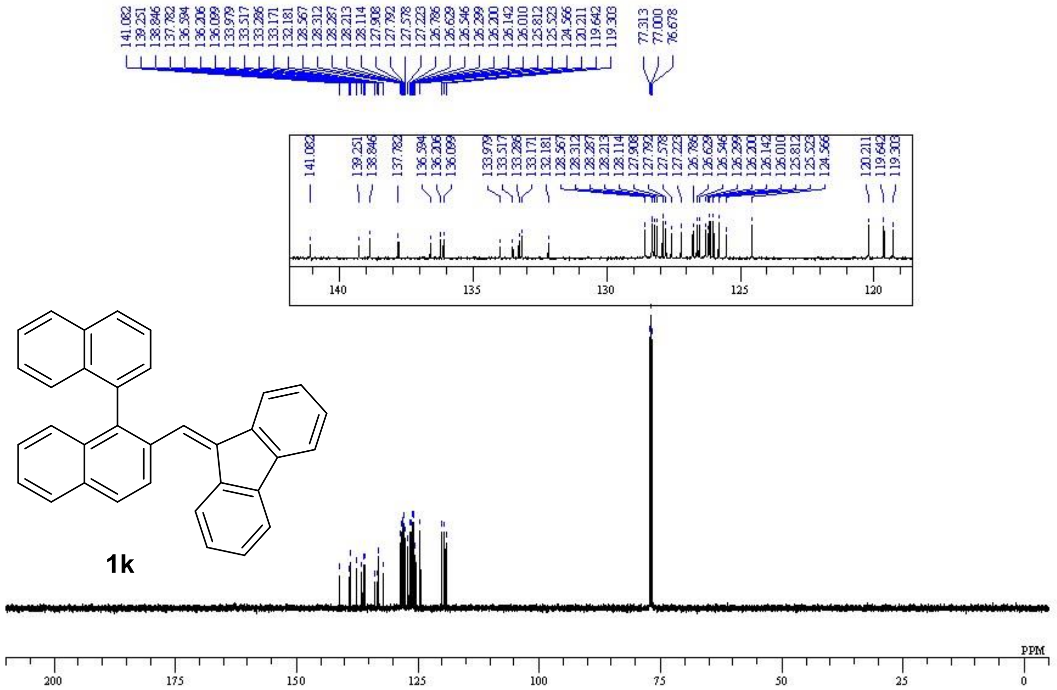

Supplementary Figure 26. ^{1}H and ^{13}C NMR spectra of compound **1k**

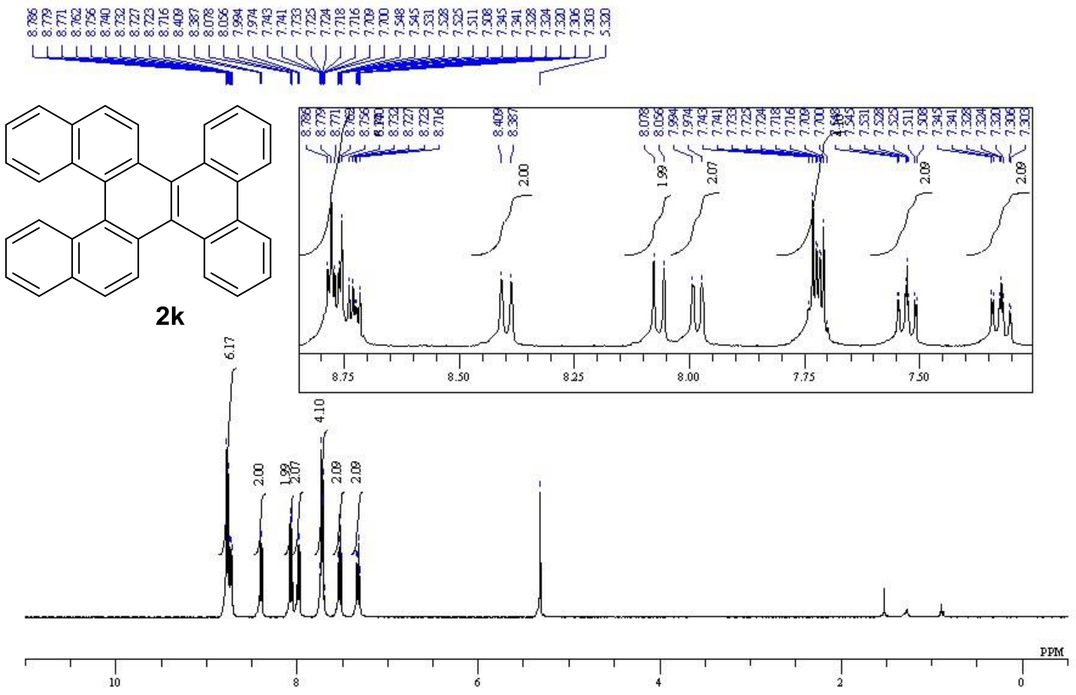

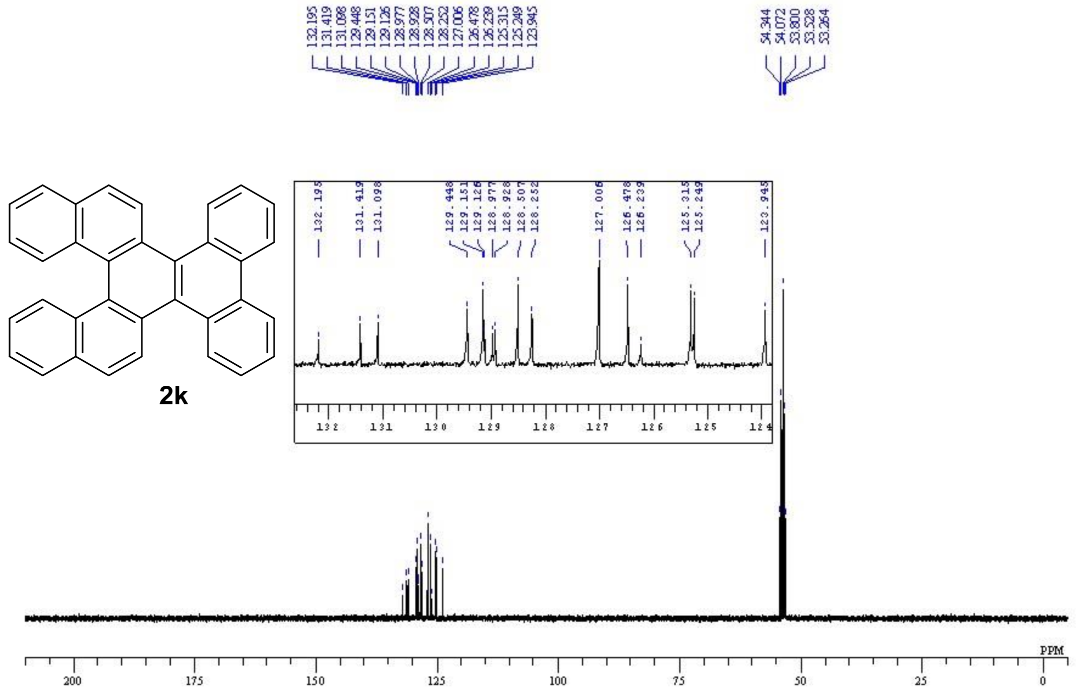

Supplementary Figure 27. ^{1}H and ^{13}C NMR spectra of compound **2k**

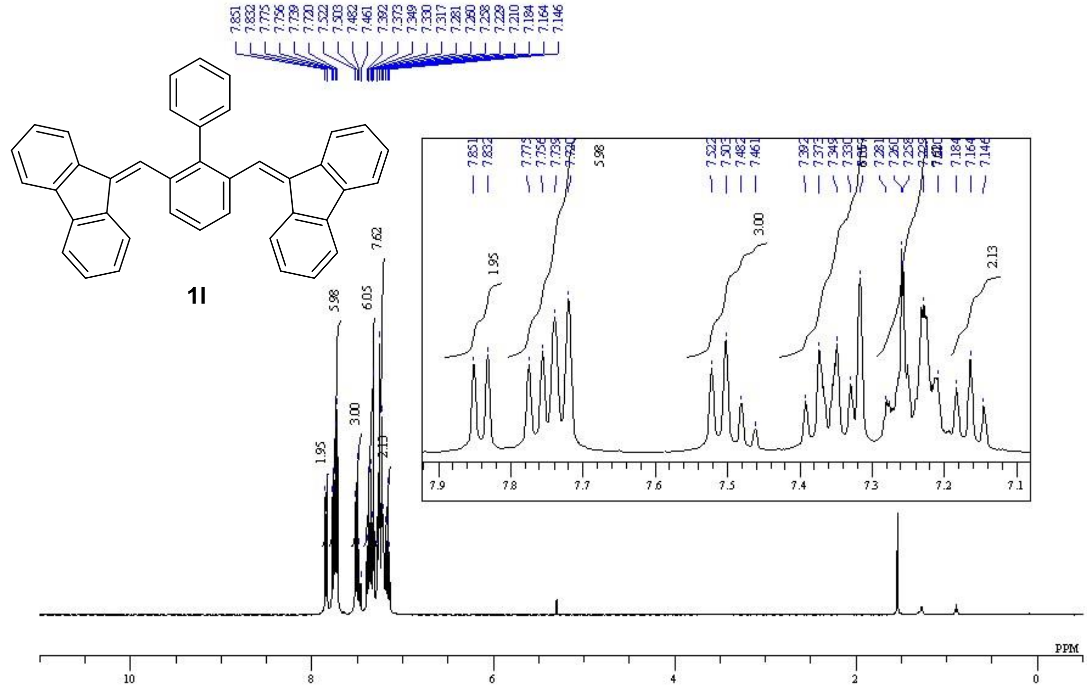

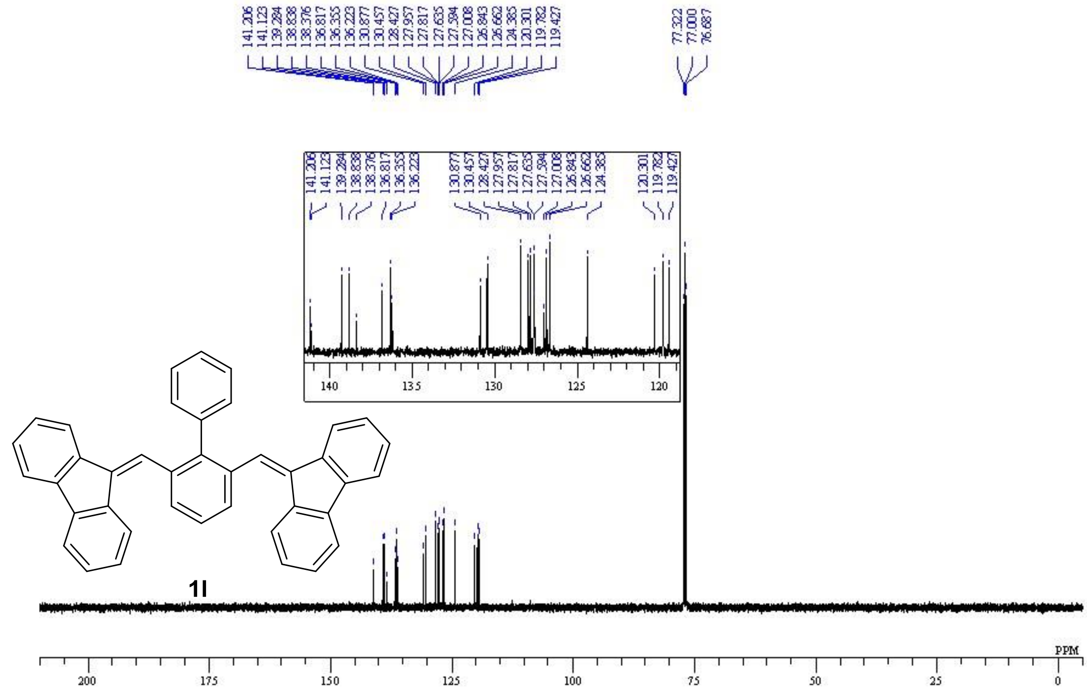

Supplementary Figure 28. ^{1}H and ^{13}C NMR spectra of compound **1l**

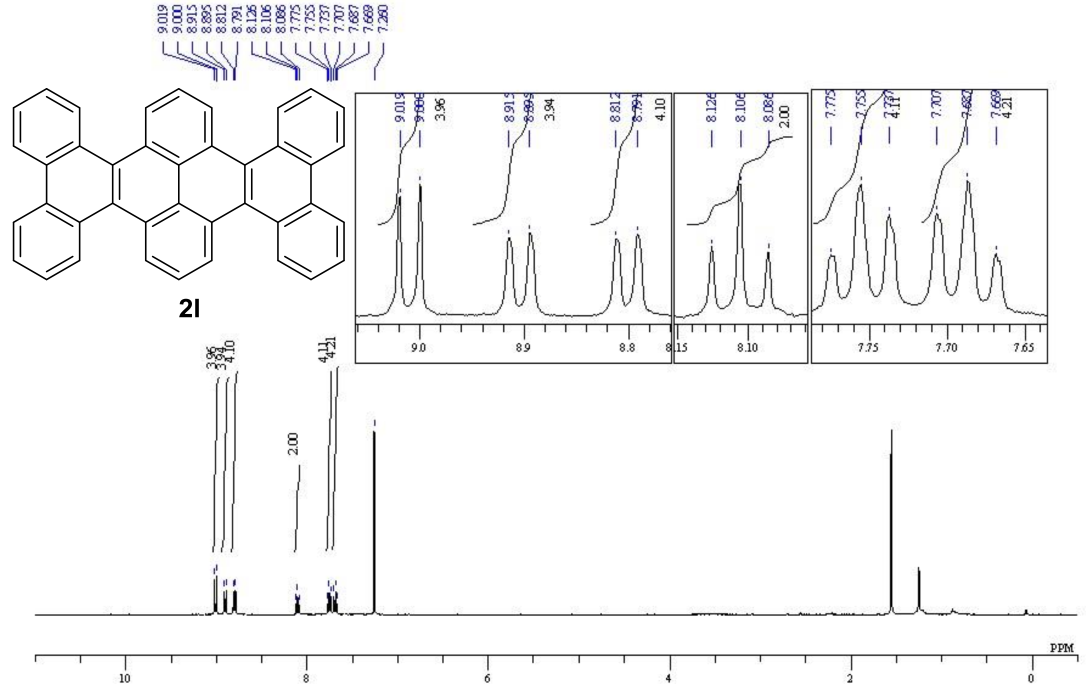

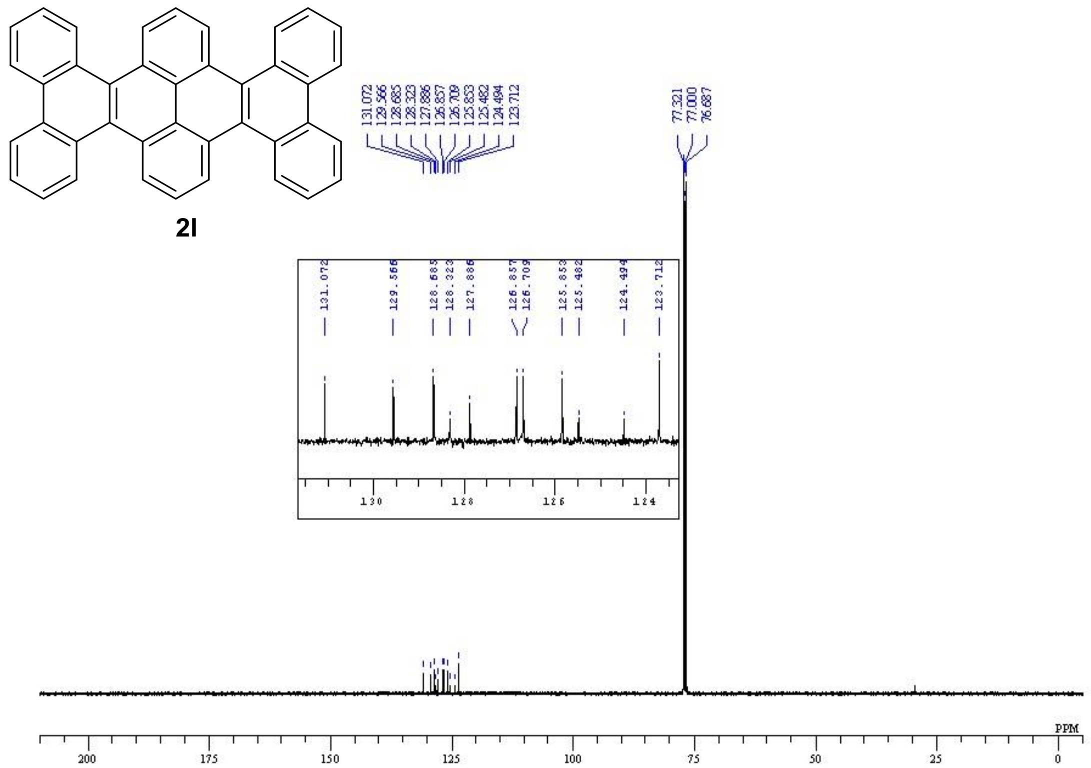

Supplementary Figure 29. ^{1}H and ^{13}C NMR spectra of compound **2l**

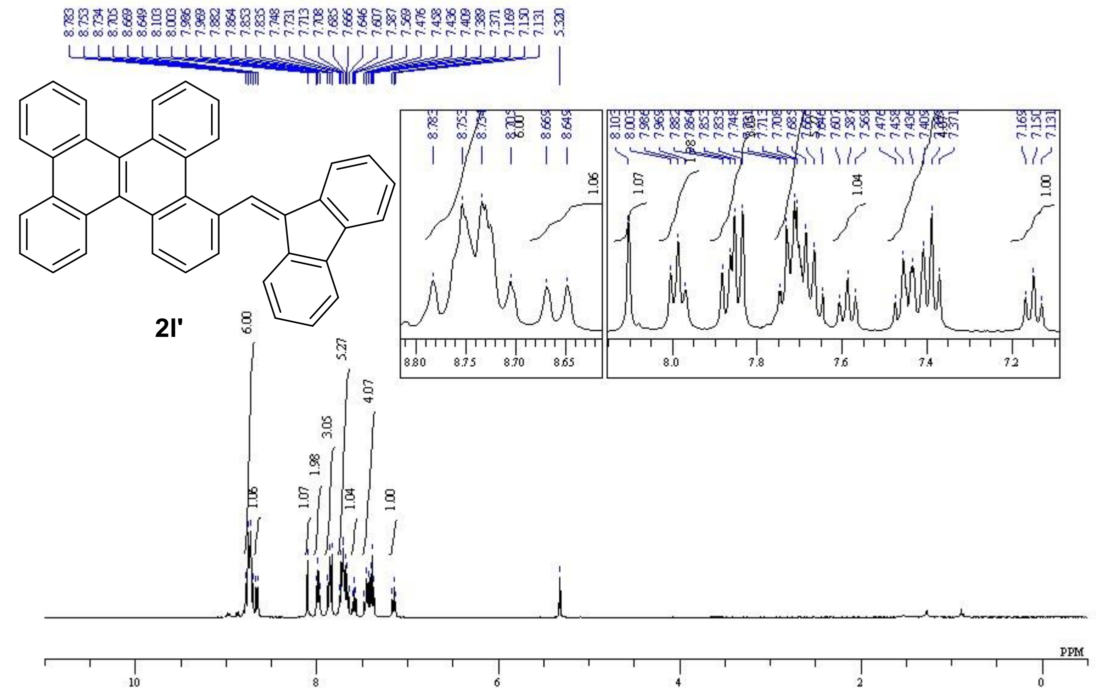

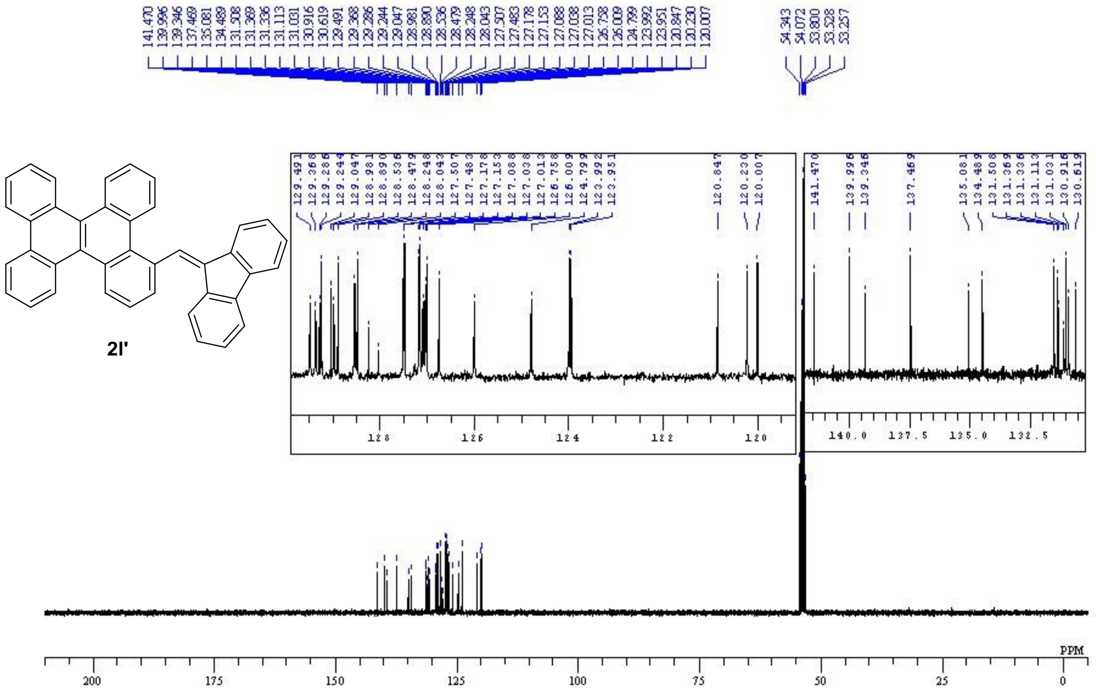

Supplementary Figure 30. ^1H and ^{13}C NMR spectra of compound **2l’**

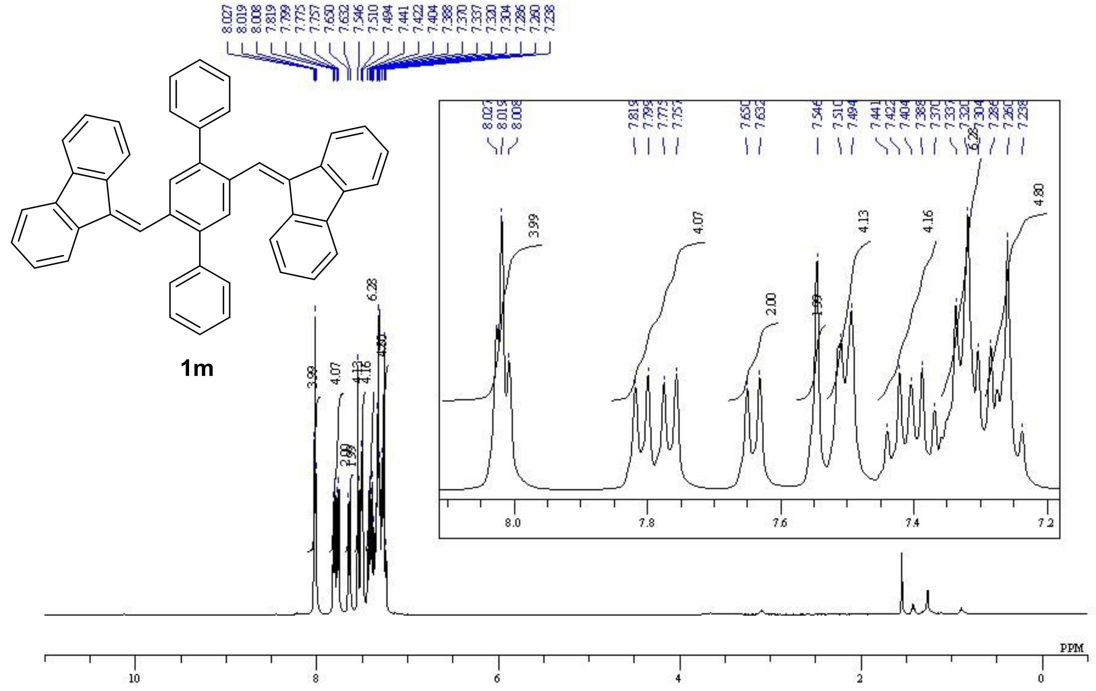

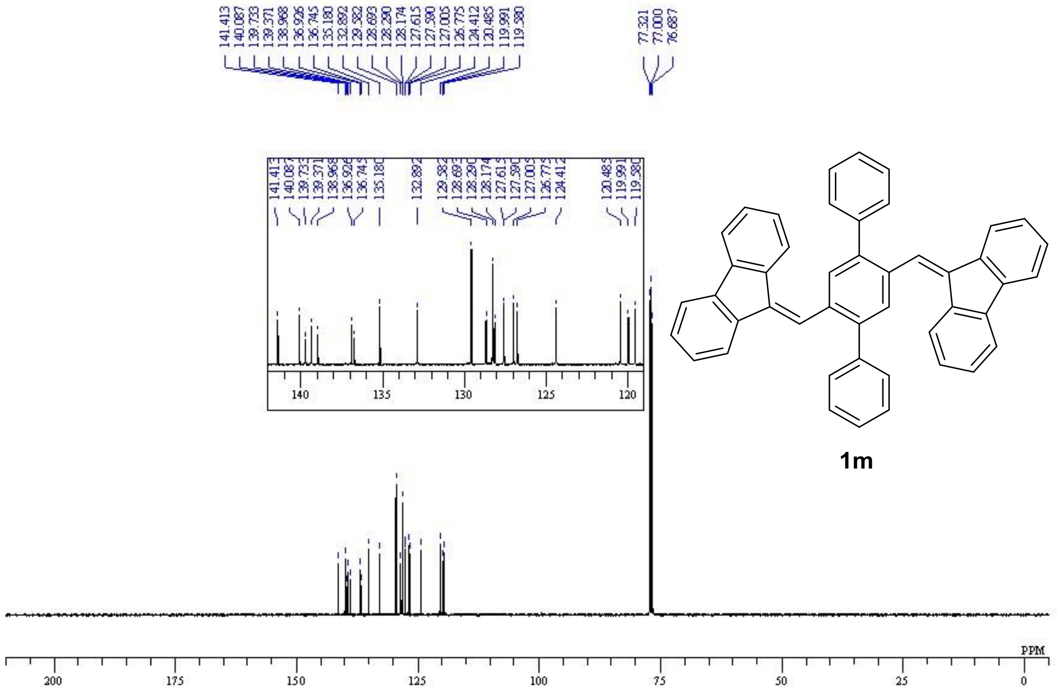

Supplementary Figure 31. ^{1}H and ^{13}C NMR spectra of compound **1m**

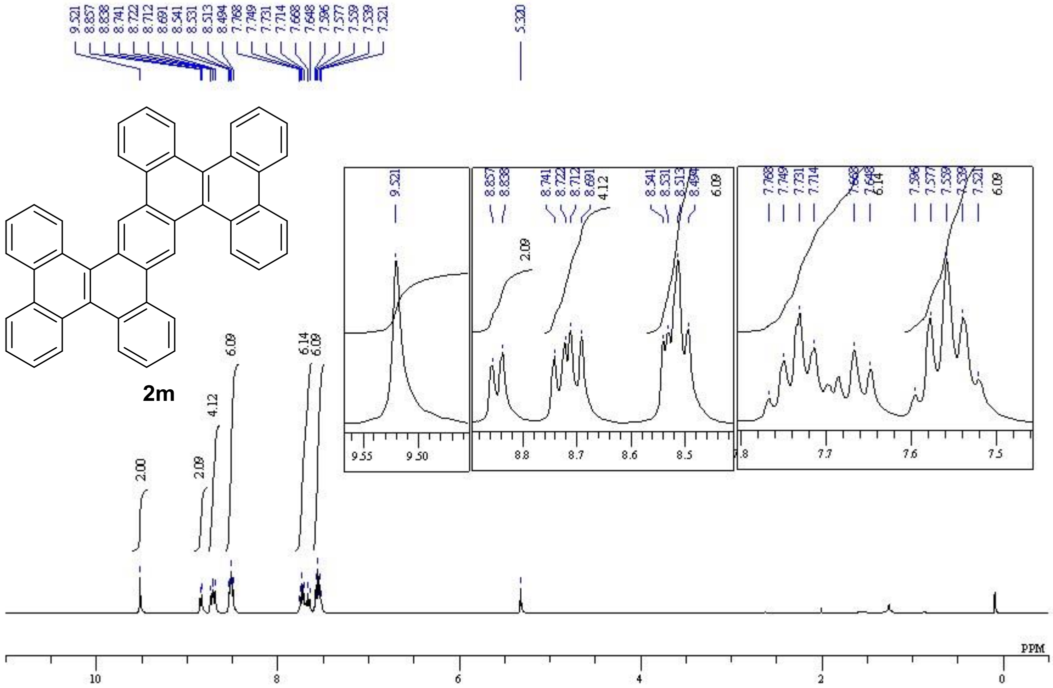

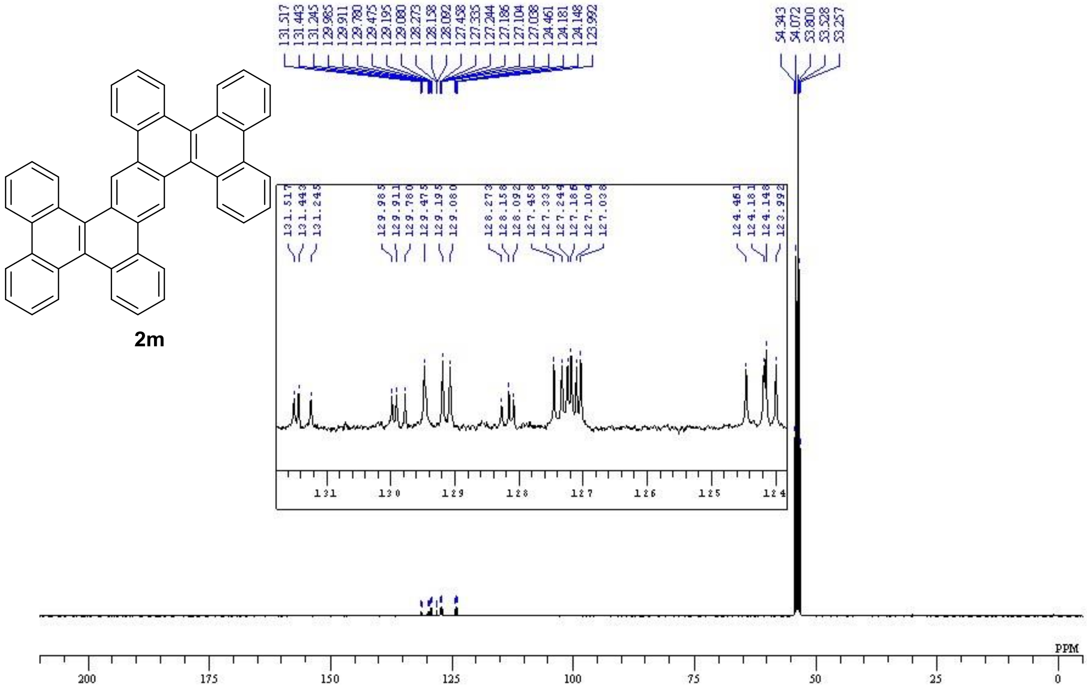

Supplementary Figure 32. ^{1}H and ^{13}C NMR spectra of compound **2m**

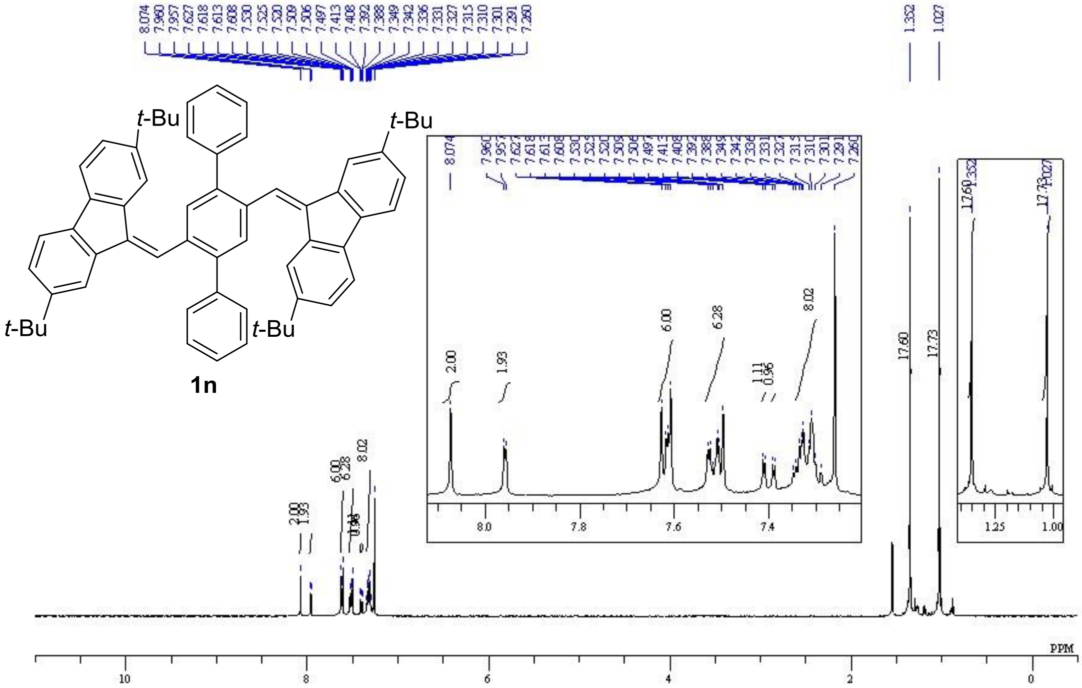

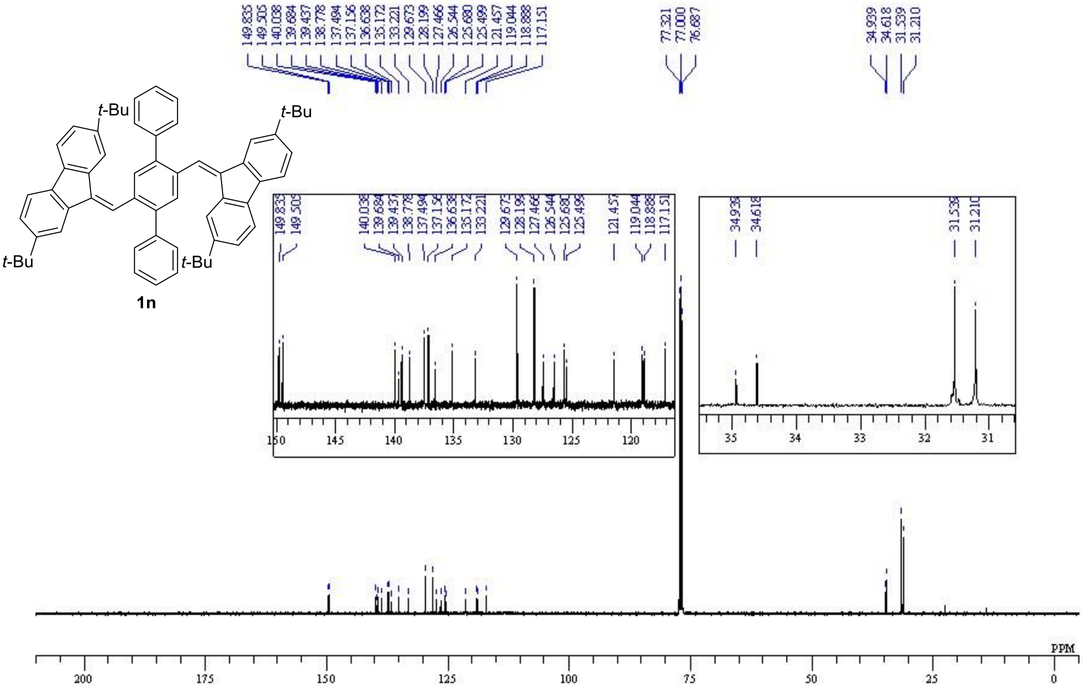

Supplementary Figure 33. ^{1}H and ^{13}C NMR spectra of compound **1n**

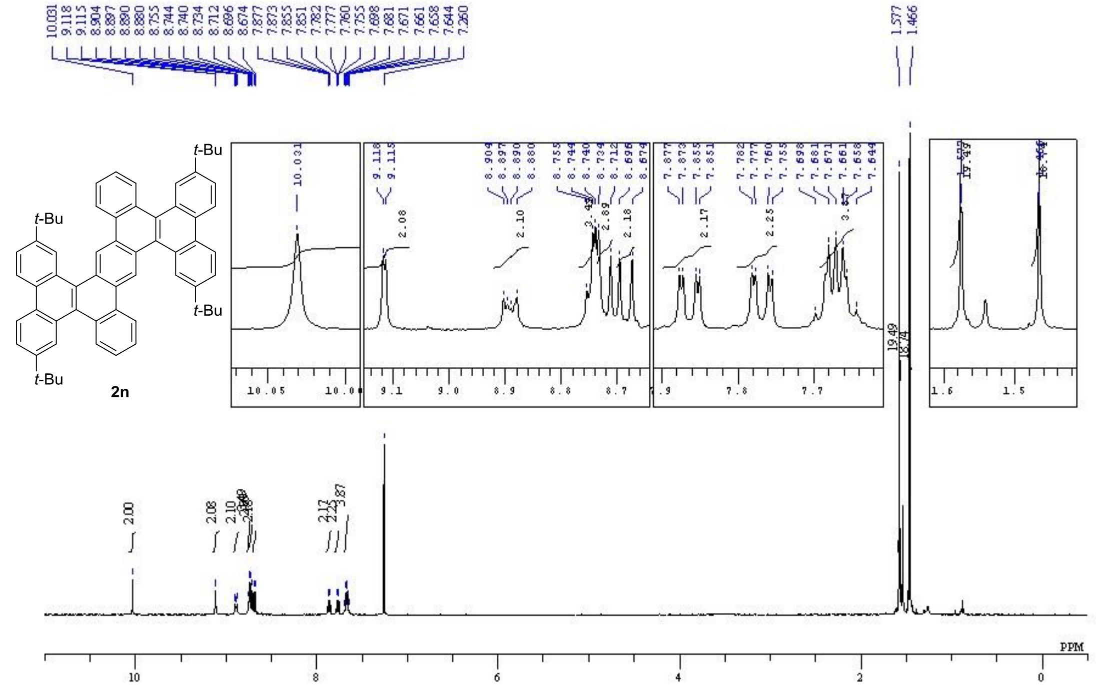

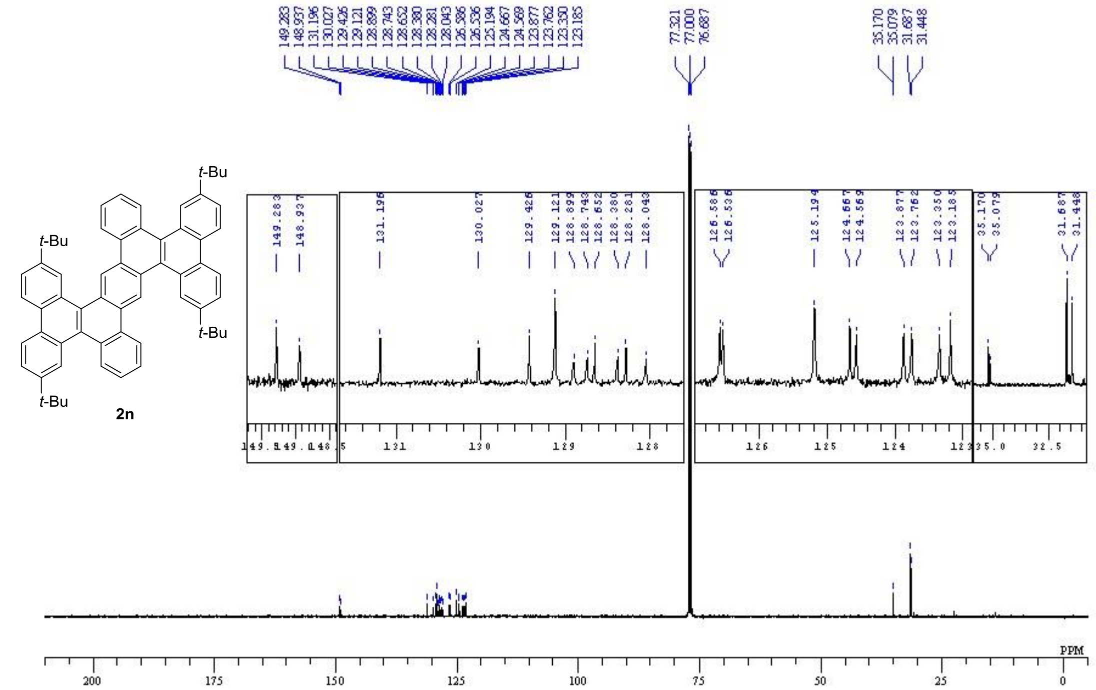

Supplementary Figure 34. ^{1}H and ^{13}C NMR spectra of compound **2n**

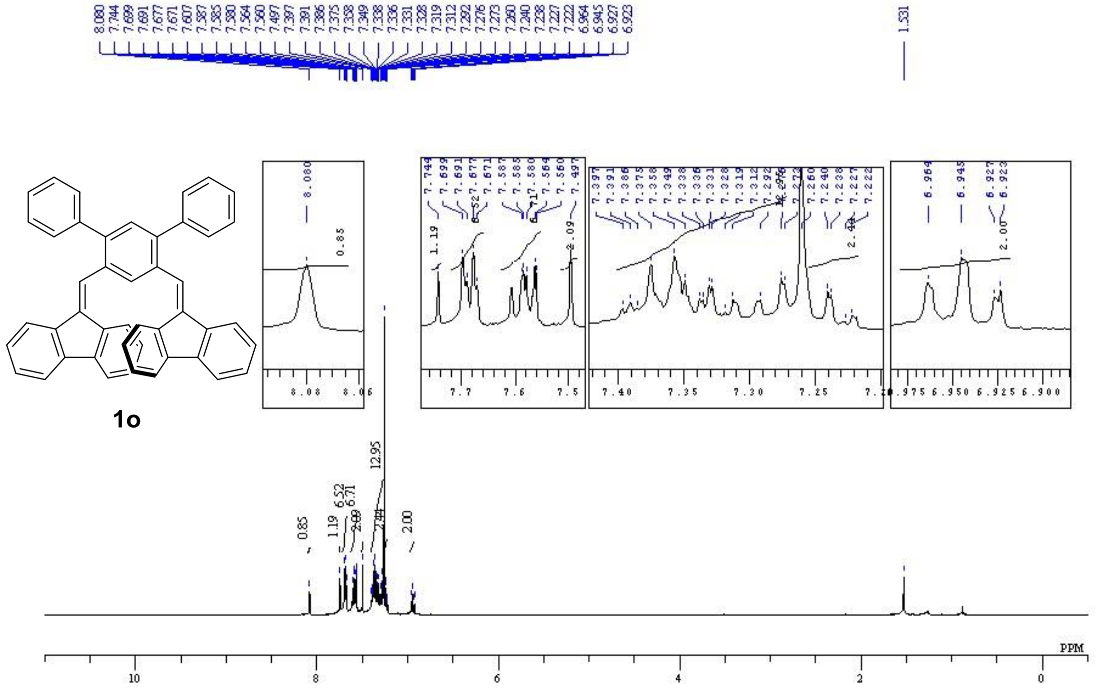

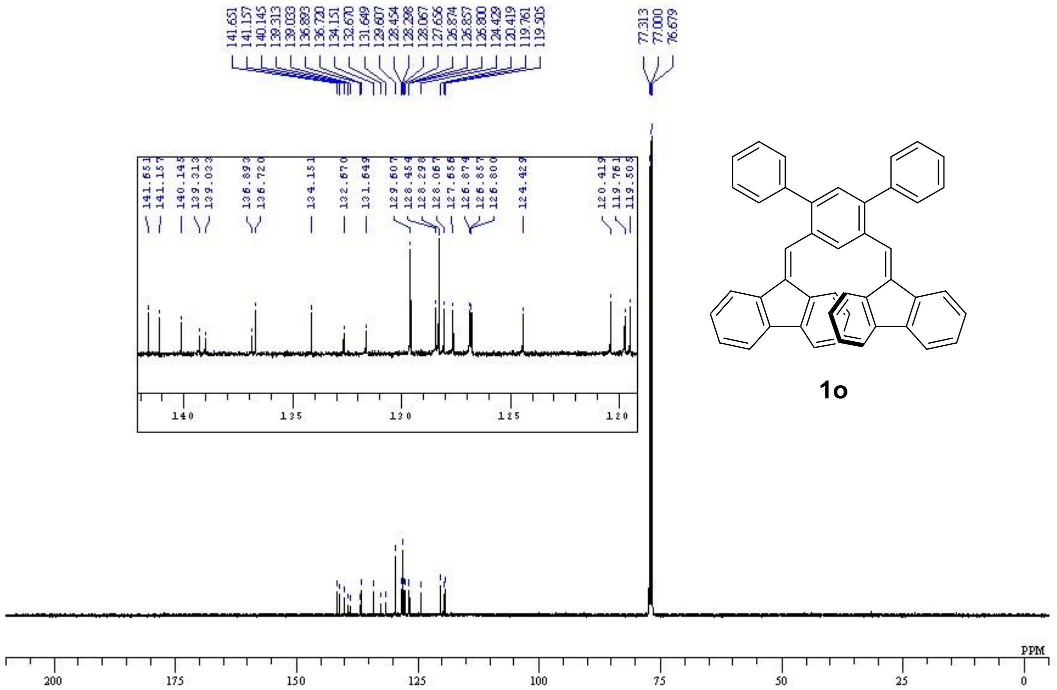

Supplementary Figure 35. ^{1}H and ^{13}C NMR spectra of compound **1o**

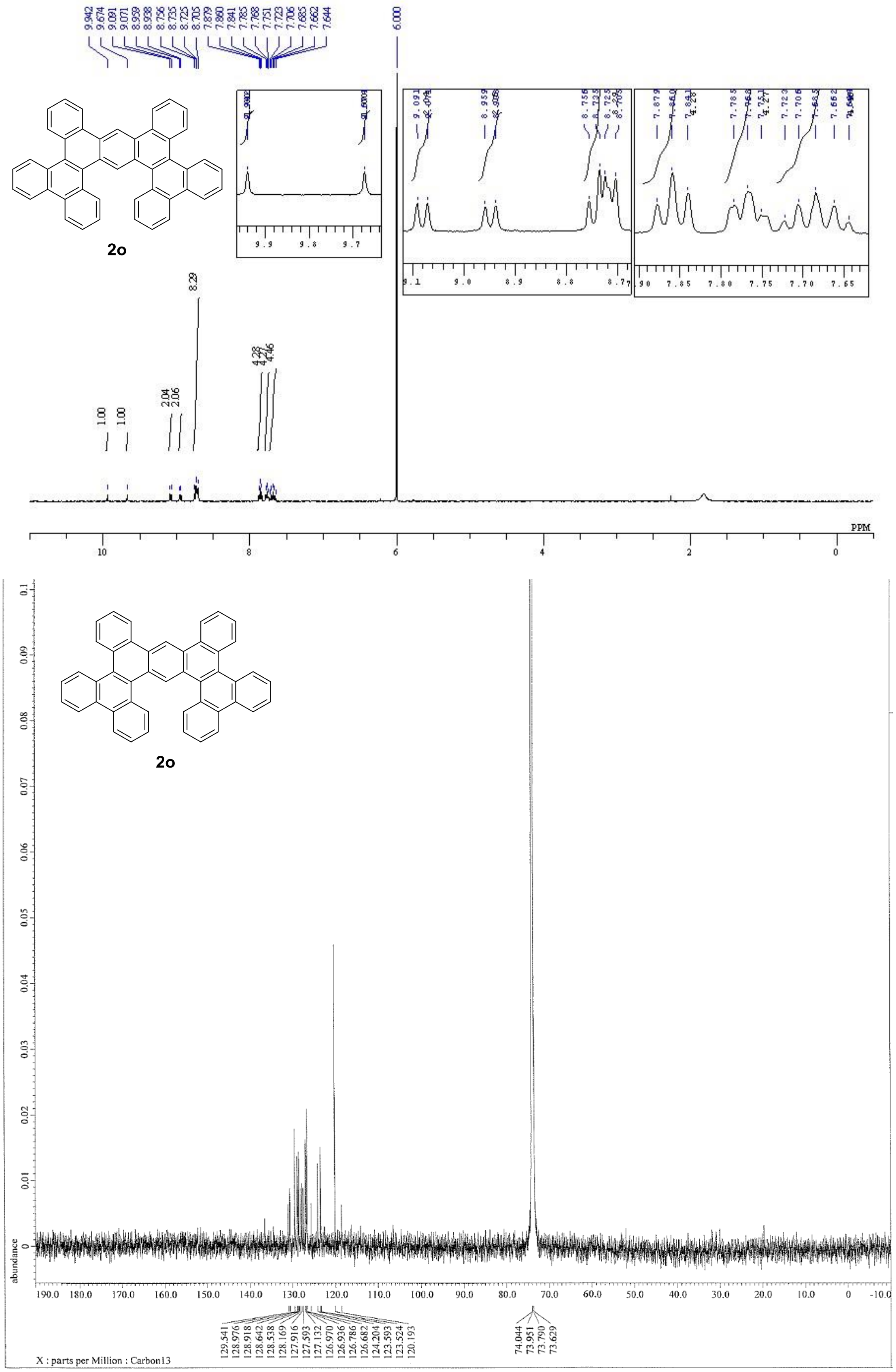

Supplementary Figure 36. ^{1}H and ^{13}C NMR spectra of compound **2o**

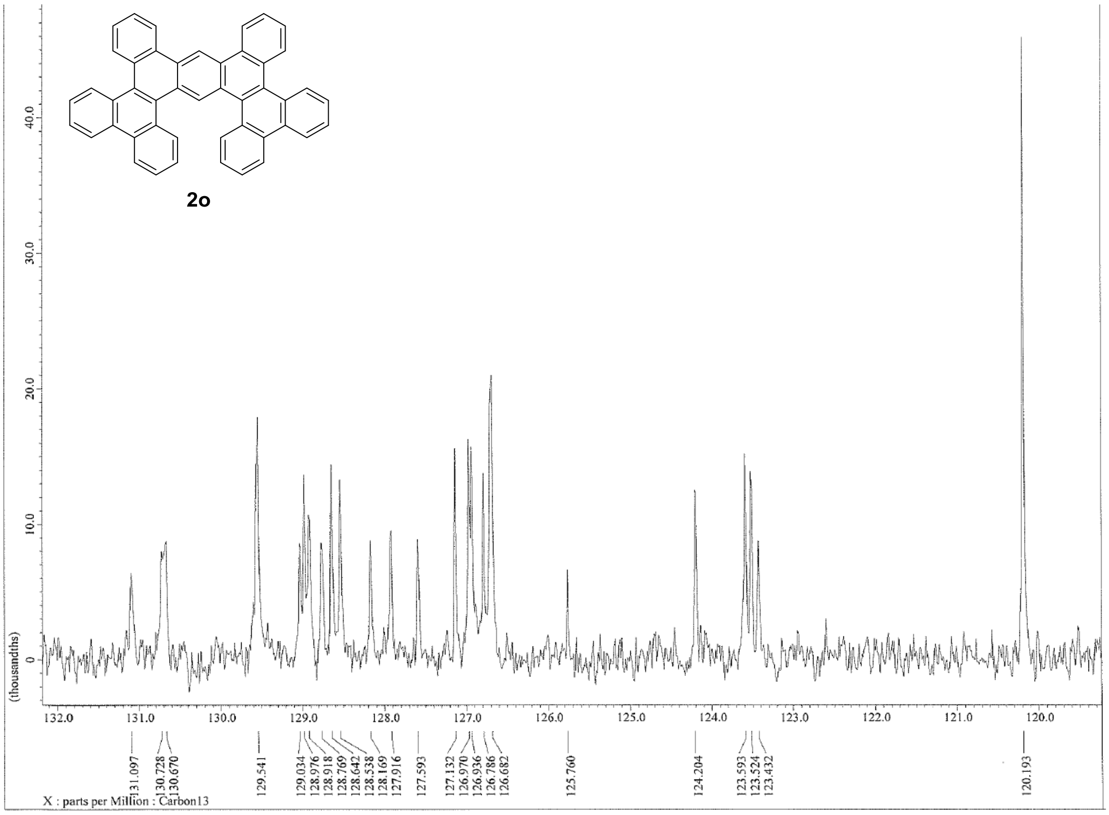

Supplementary Figure 37. Magnified ^{13}C NMR spectrum of compound **2o**

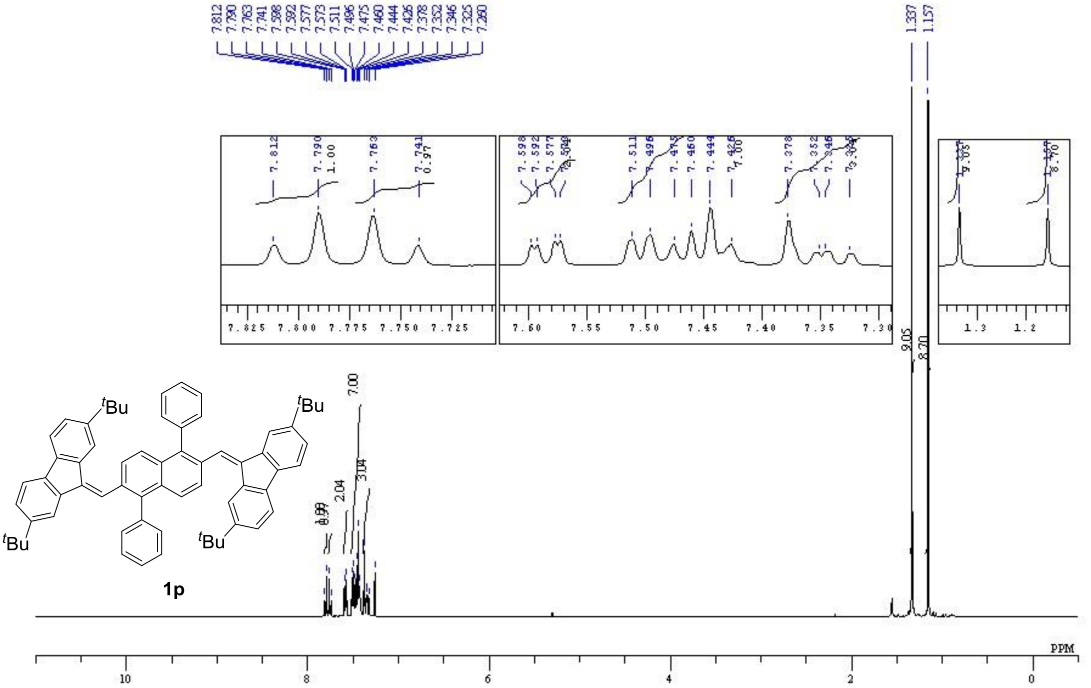

Supplementary Figure 38. ^{1}H NMR spectrum of compound **1p**

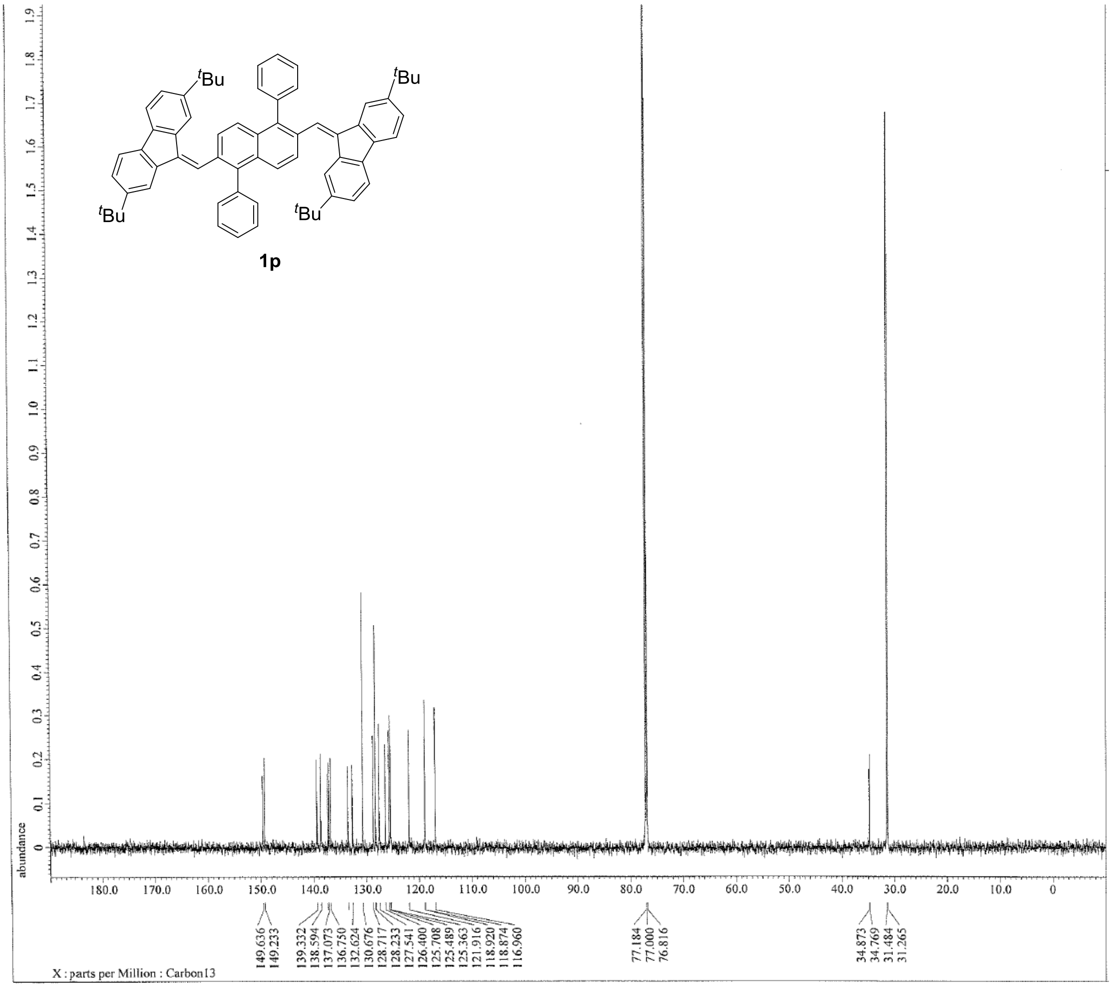

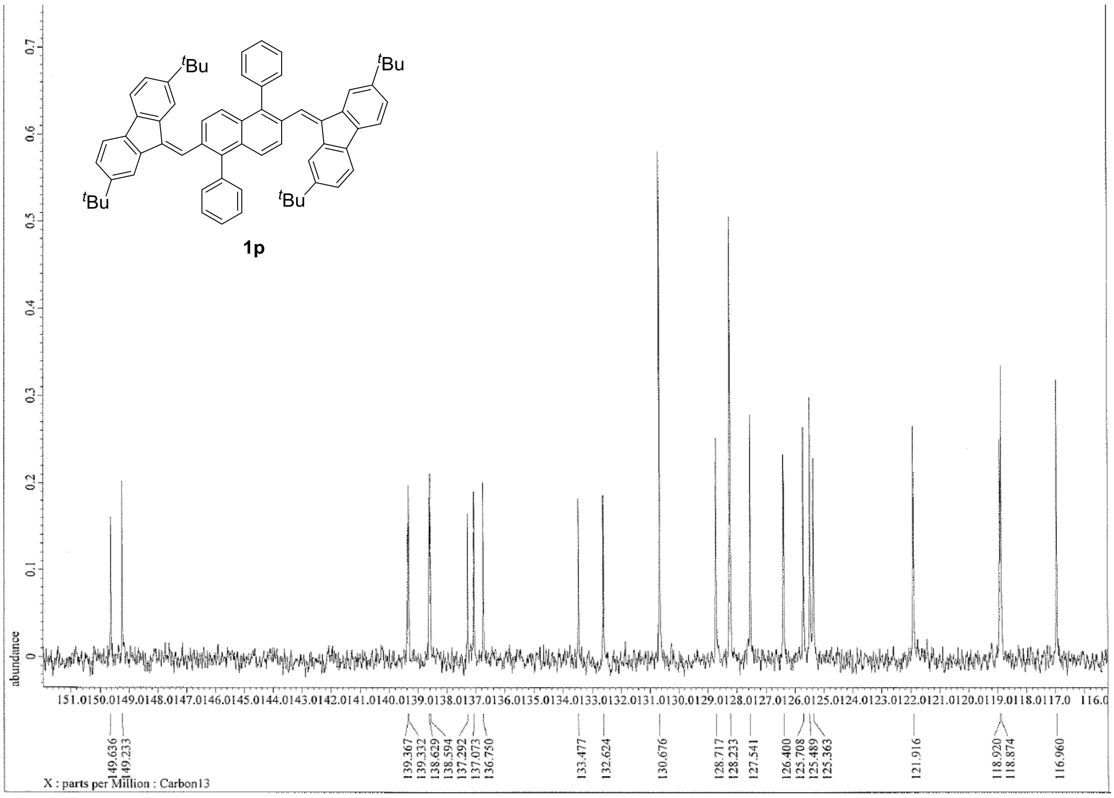

Supplementary Figure 39. ^{13}C NMR spectra of compound **1p**

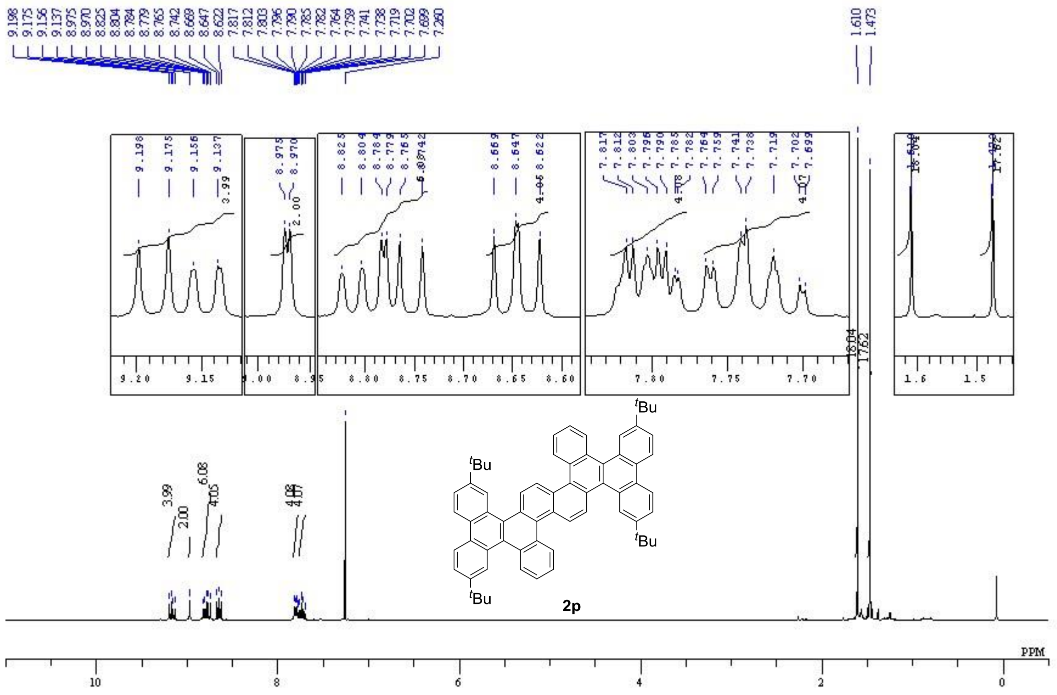

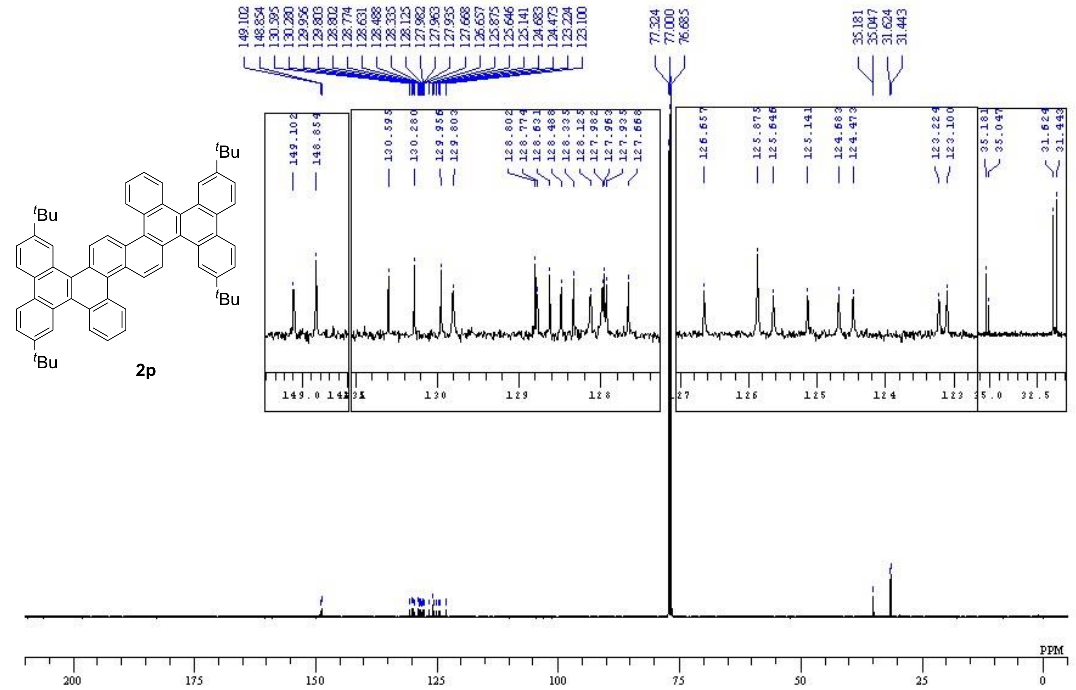

Supplementary Figure 40. ^{1}H and ^{13}C NMR spectra of compound **2p**

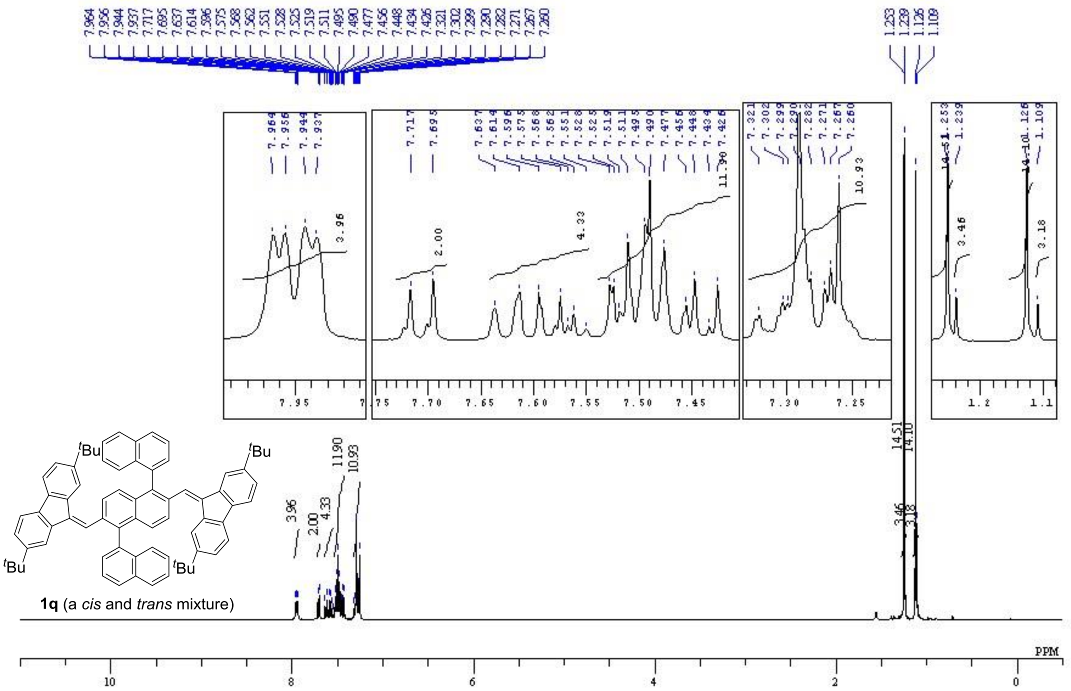

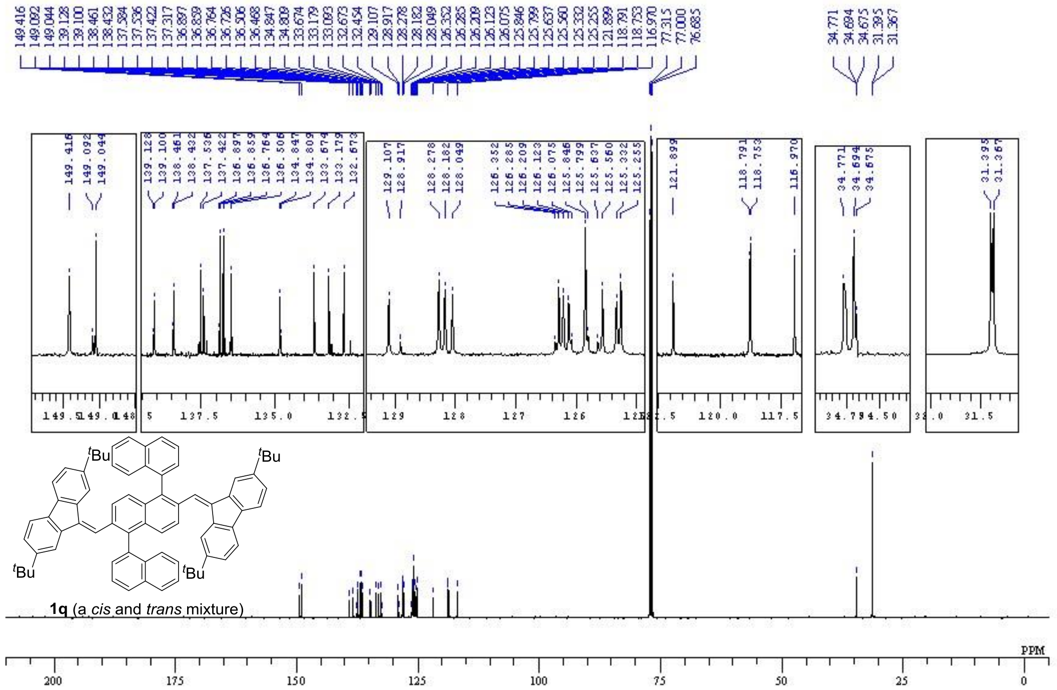

Supplementary Figure 41. ^{1}H and ^{13}C NMR spectra of compound **1q**

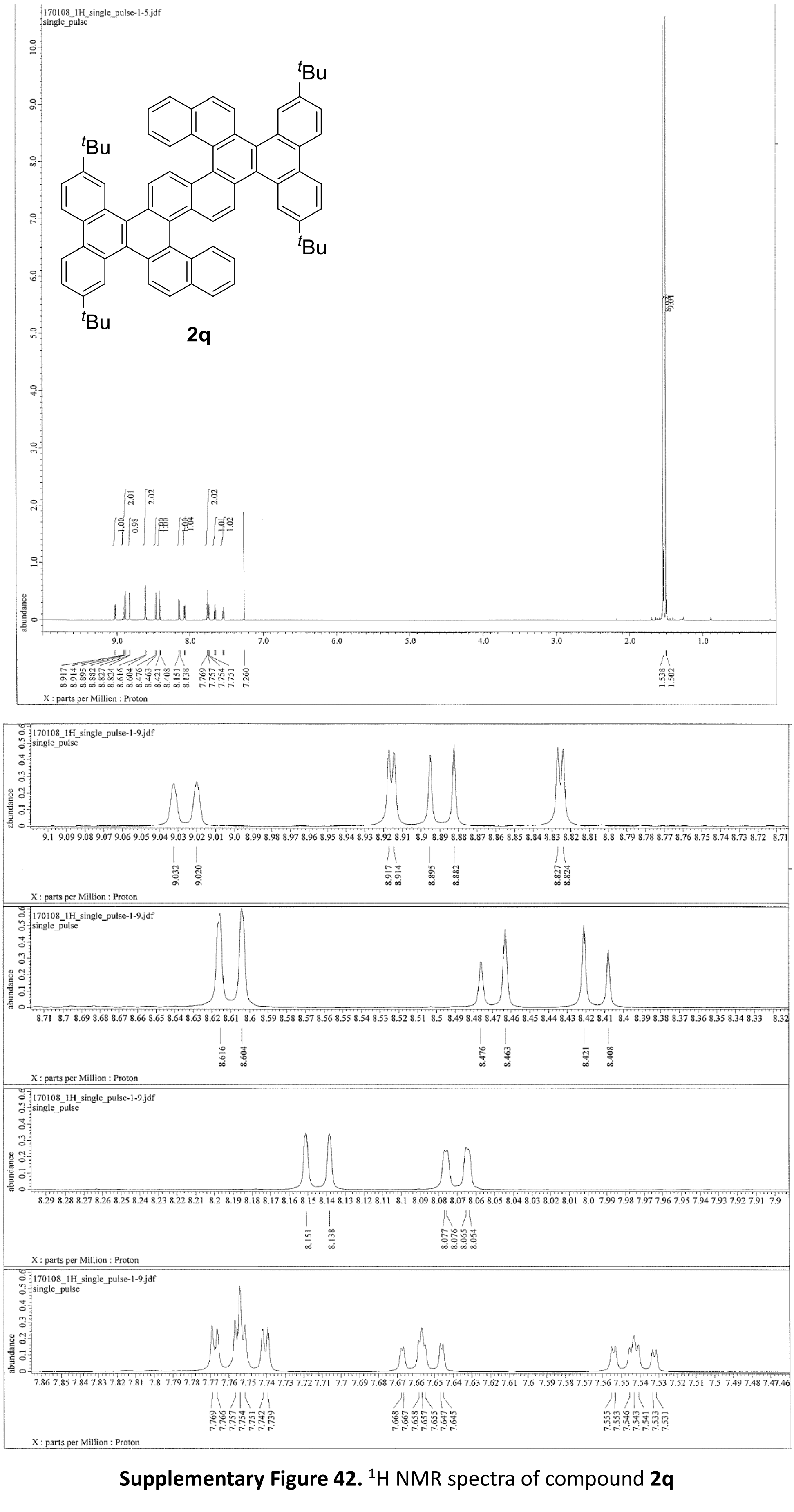

Supplementary Figure 42. ^{1}H NMR spectra of compound **2q**

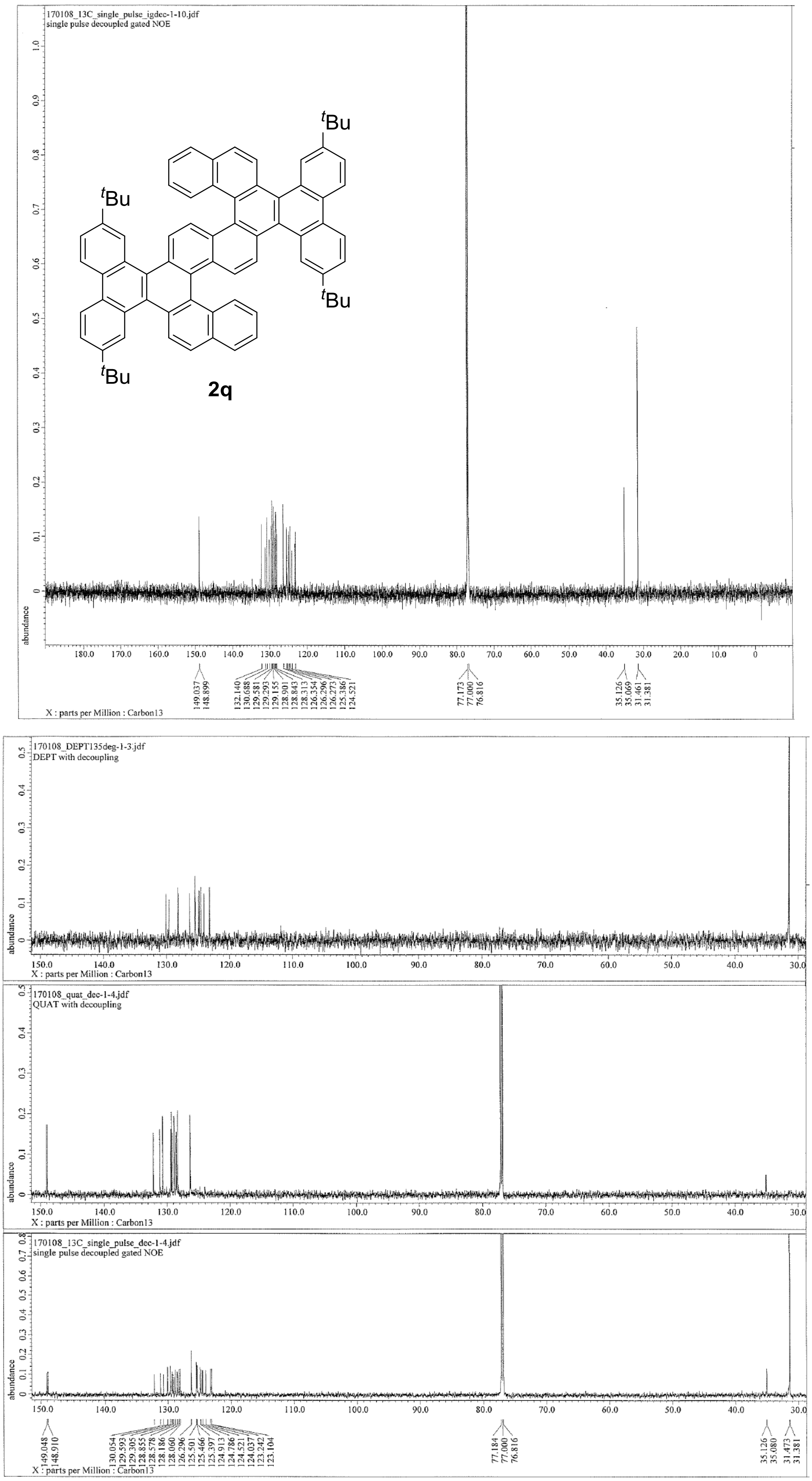

Supplementary Figure 43. ^{13}C NMR, DEPT, and QUAT spectra of compound **2q**

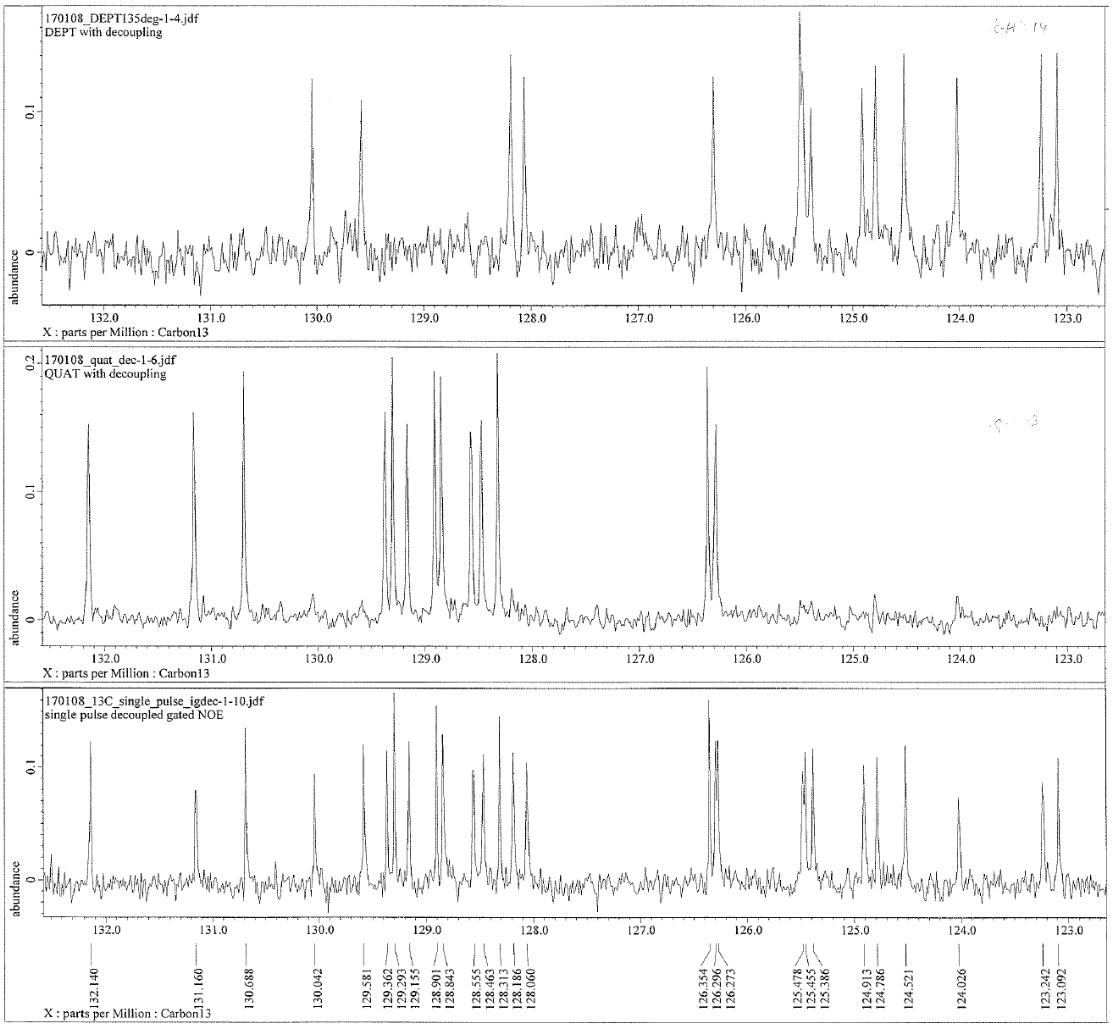

Supplementary Figure 44. Magnified ^{13}C NMR, DEPT, and QUAT spectra of compound **2q**

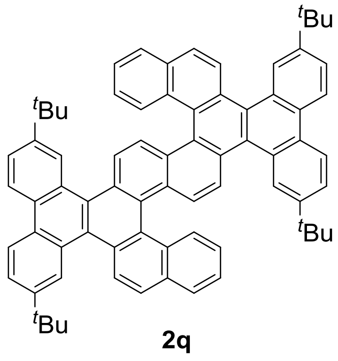

2q

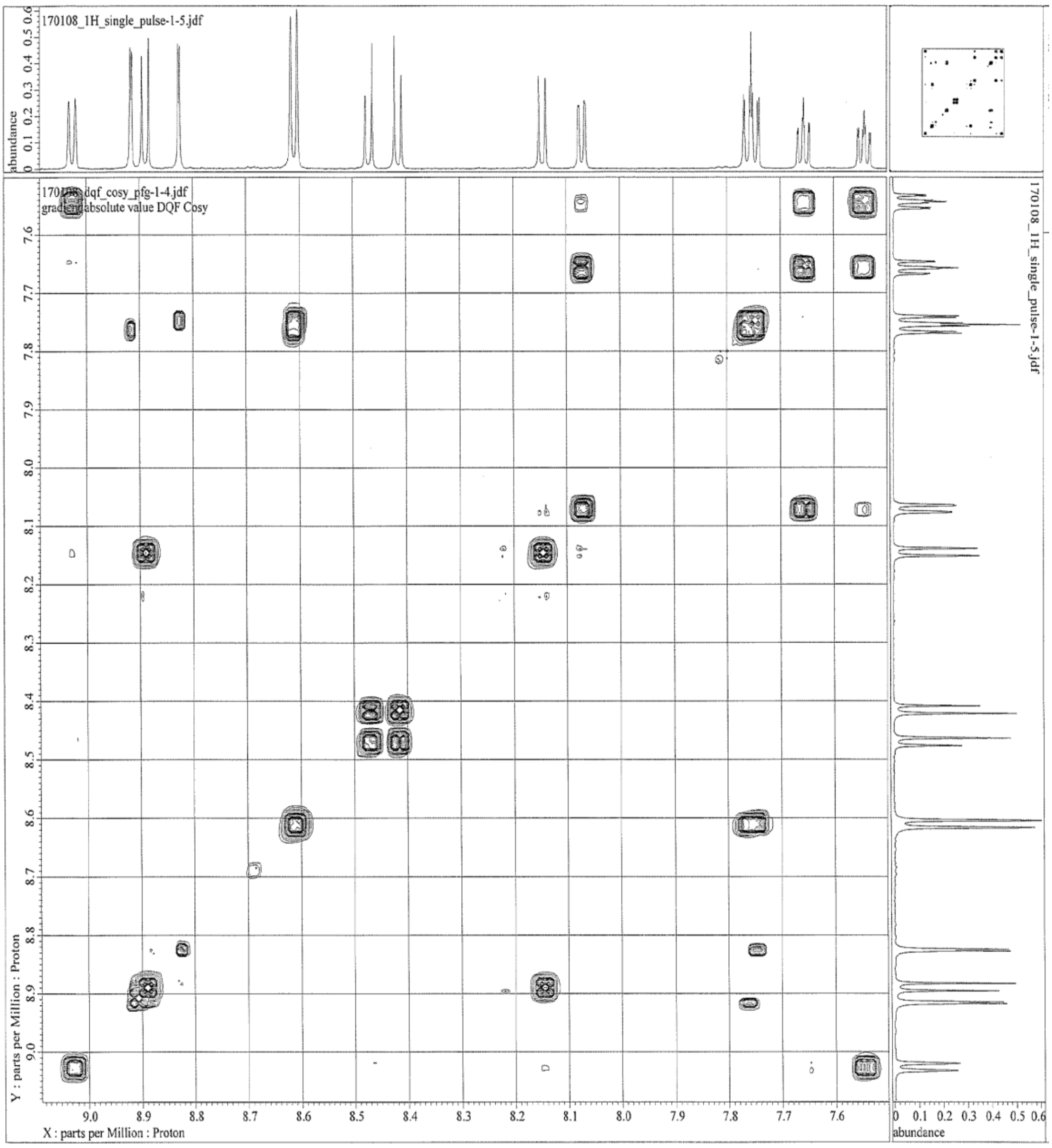

Supplementary Figure 45. DQF COSY spectrum of compound **2q**

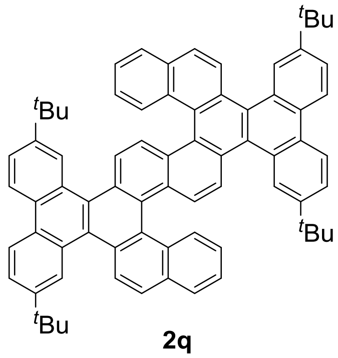

2q

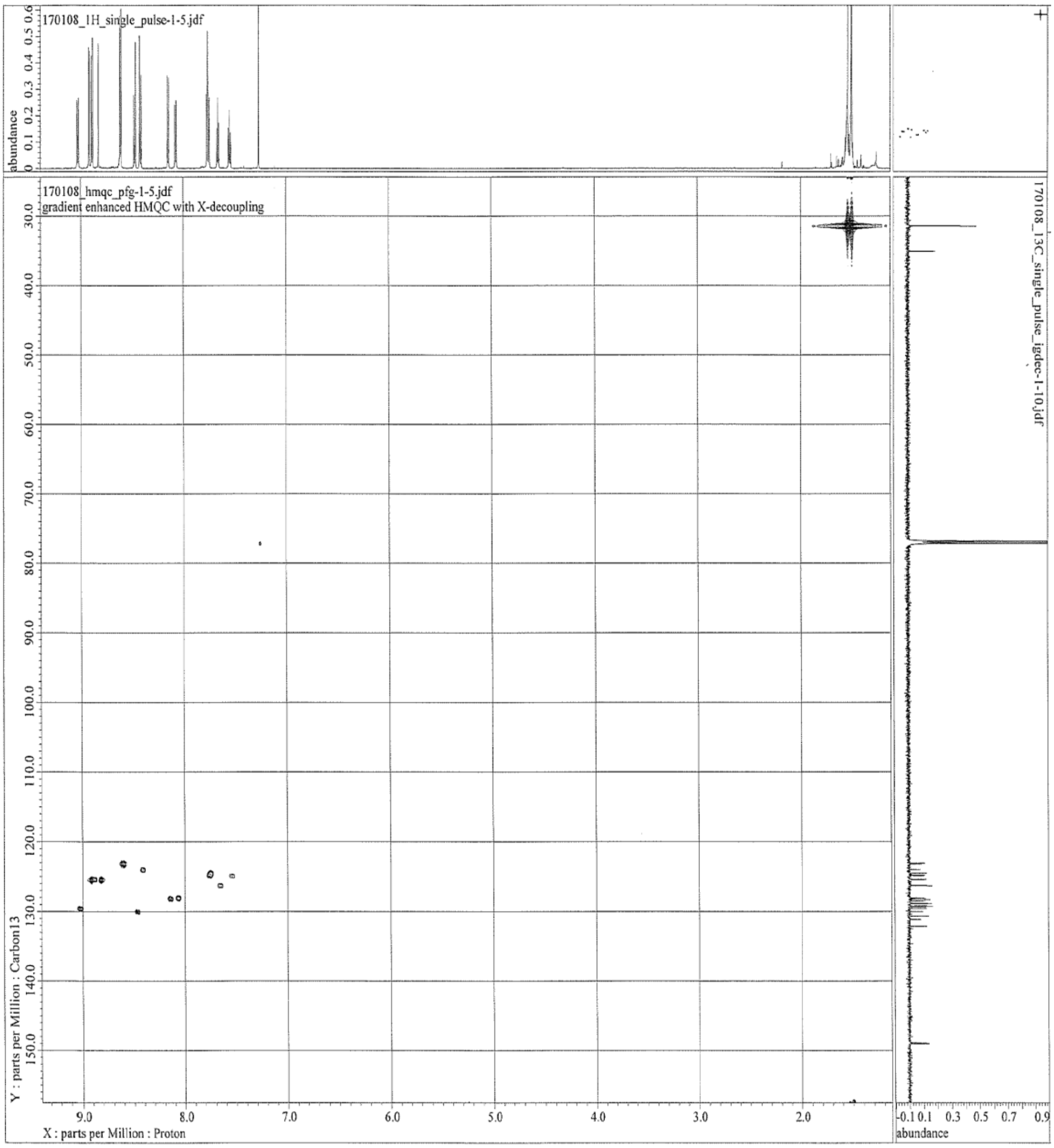

Supplementary Figure 46. HMQC spectrum of compound **2q**

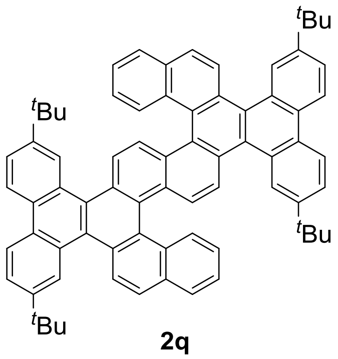

2q

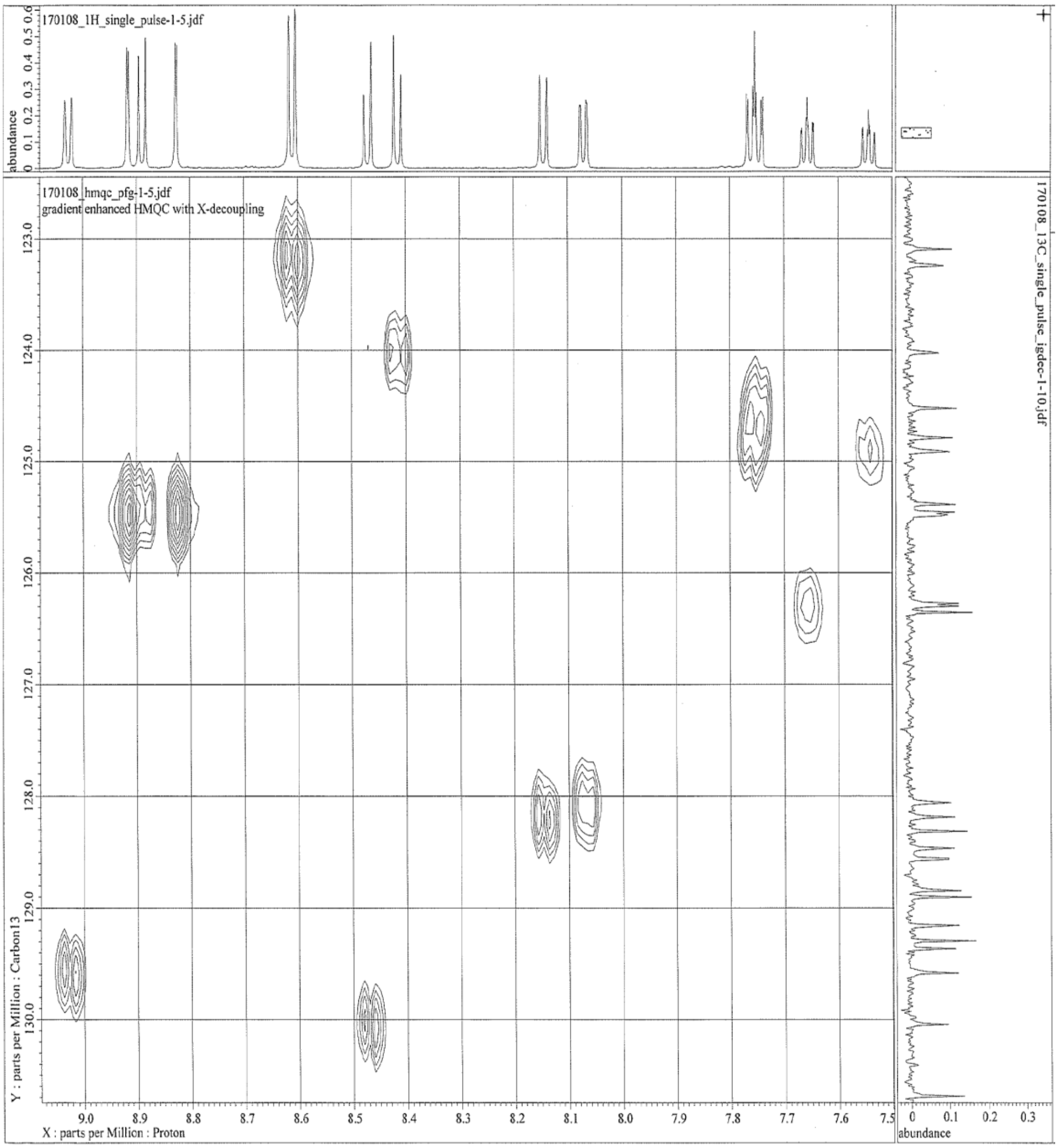

Supplementary Figure 47. Magnified HMQC spectrum of compound **2q**

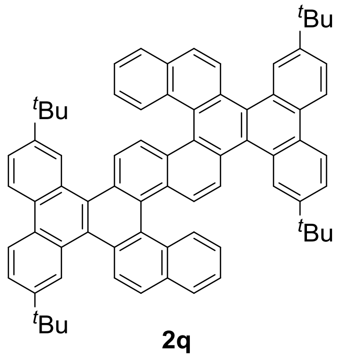

2q

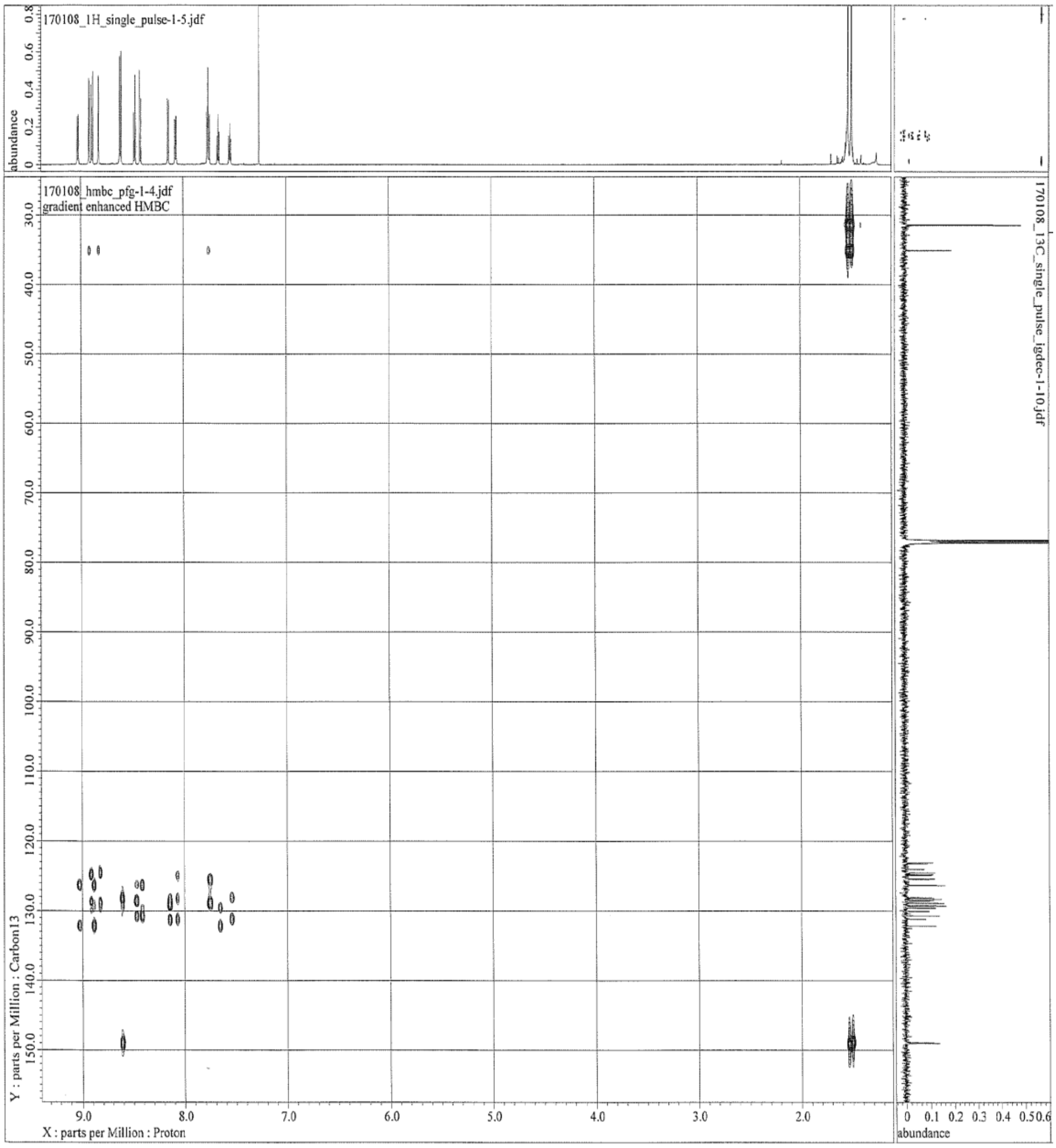

Supplementary Figure 48. HMBC spectrum of compound **2q**

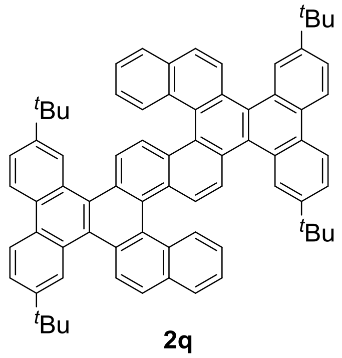

2q

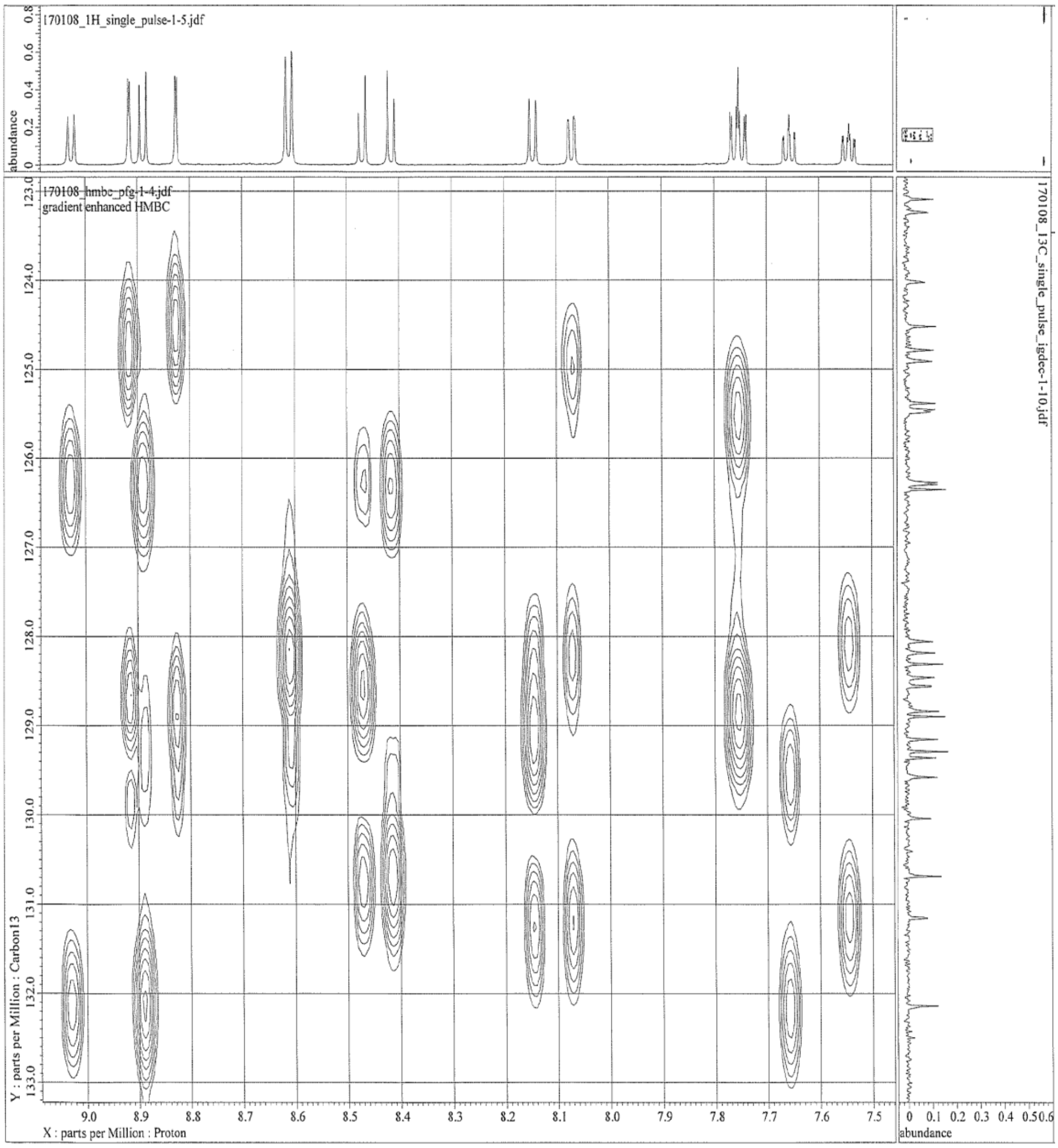

Supplementary Figure 49. Magnified HMBC spectrum of compound **2q**

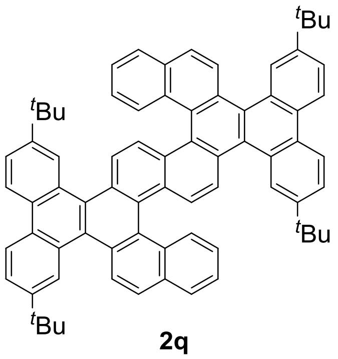

2q

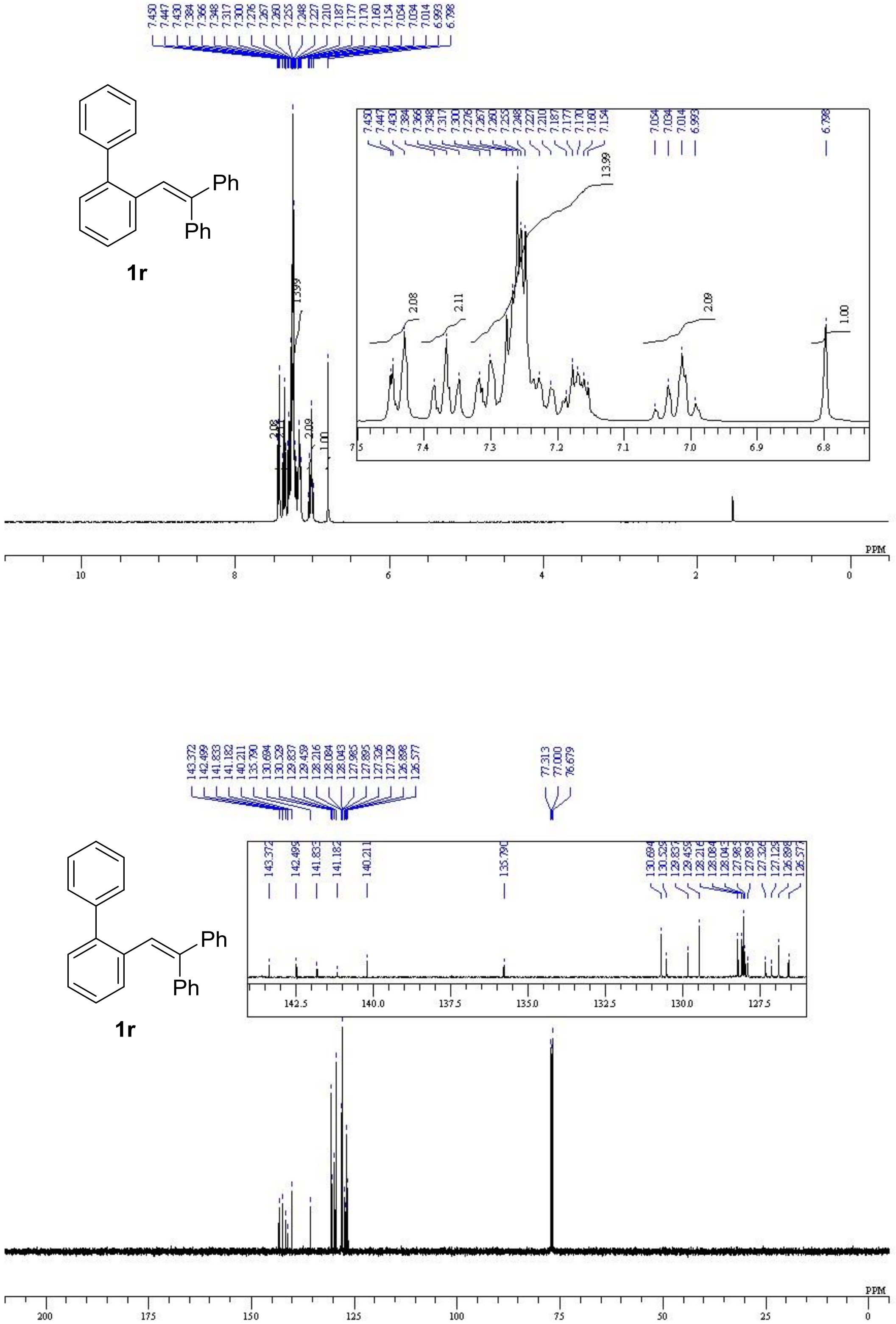

Supplementary Figure 50. ^{1}H and ^{13}C NMR spectra of compound **1r**

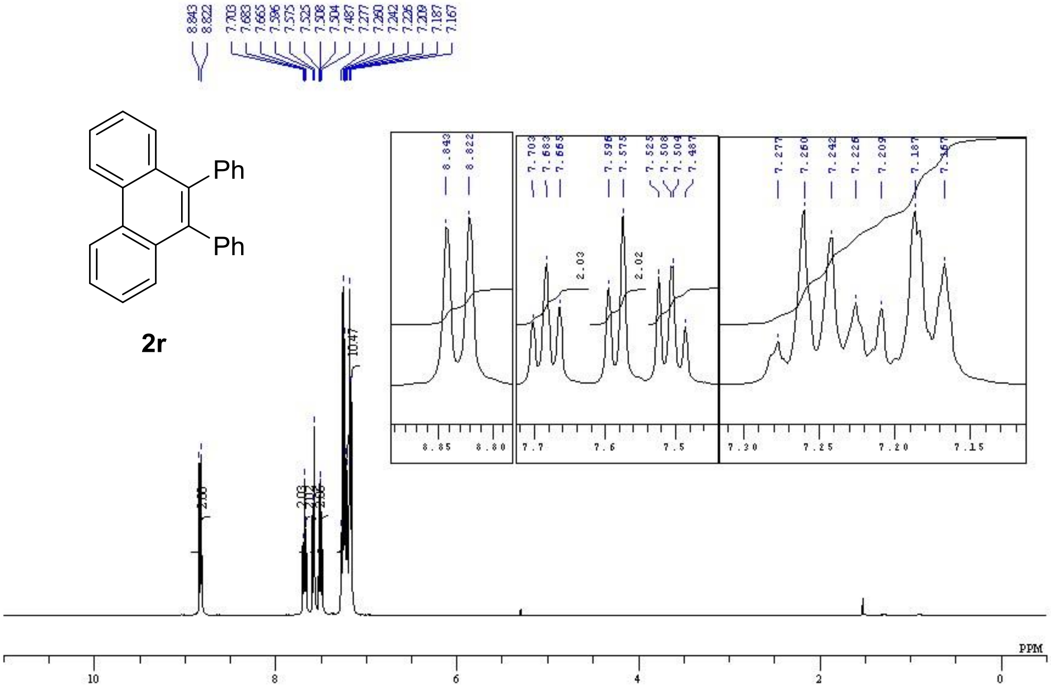

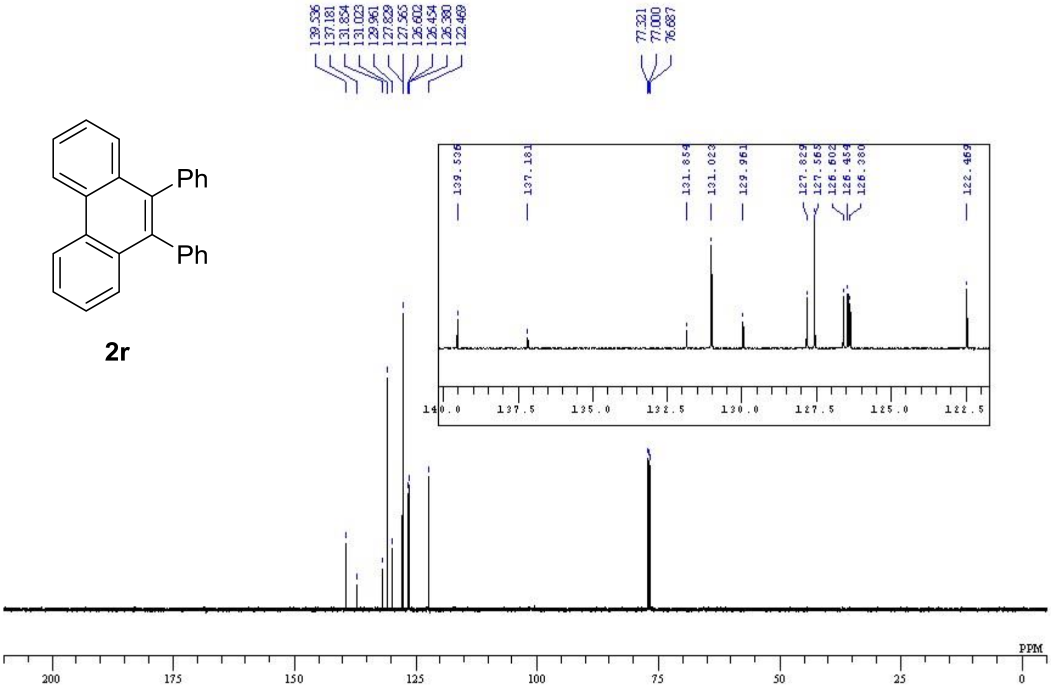

Supplementary Figure 51. ^{1}H and ^{13}C NMR spectra of compound **2r**

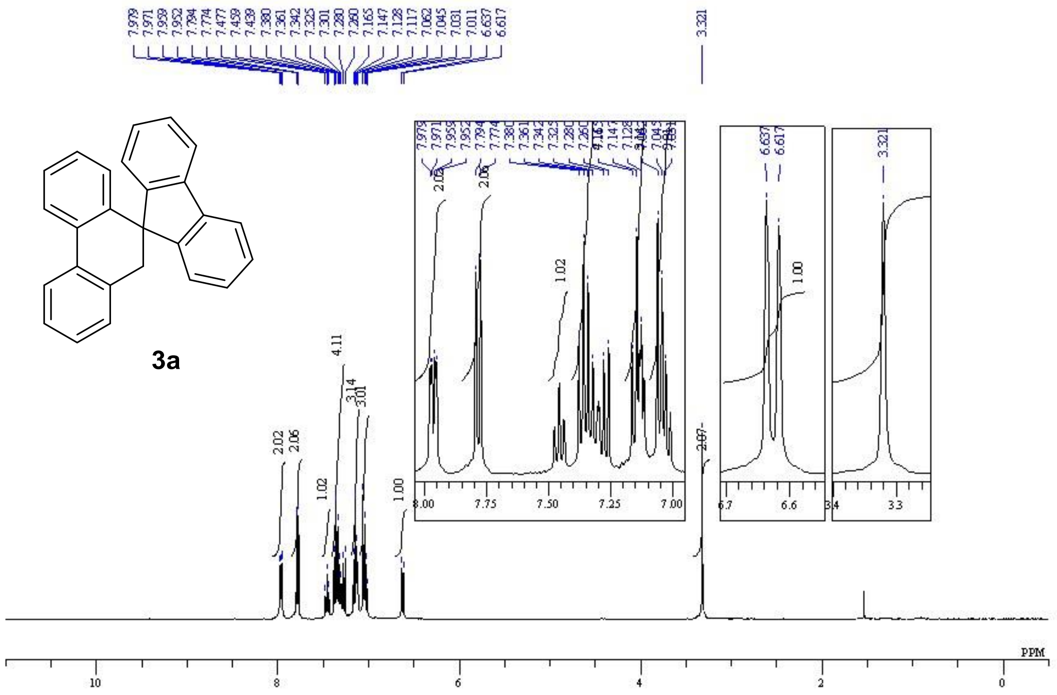

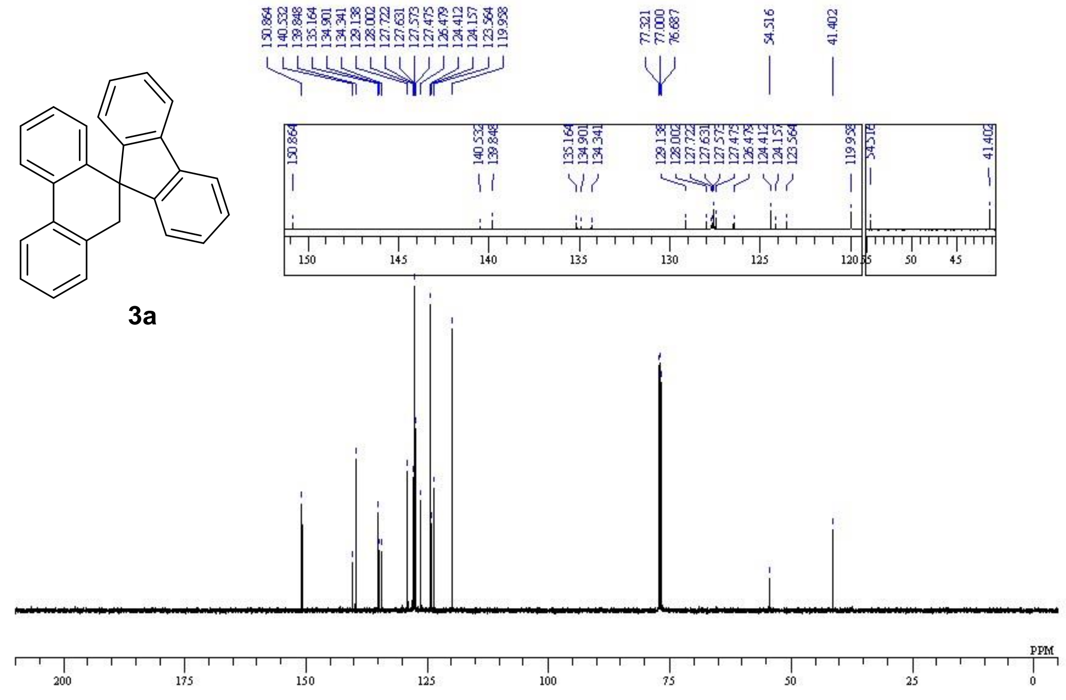

Supplementary Figure 52. ^{1}H and ^{13}C NMR spectra of compound **3a**

Supplementary References

1. Suzuki, N., Fujita, T. & Ichikawa, J. Method for the Synthesis of Dibenzo[*g*,*p*]Chrysenes: Domino Friedel–Crafts-Type Cyclization of Difluoroethenes Bearing Two Biaryl Groups. *Org. Lett*. **17**, 4984–4987 (2015).
2. Ozaki, K., Kawasumi, K., Shibata, M., Ito, H. & Itami, K. One-shot K-region-selective annulative π-extension for nanographene synthesis and functionalization. *Nat. Commun*. **6**, 6251 (2015).
3. Kashihara, H., Asada, T. & Kamikawa, K. Synthesis of a Double Helicene by a Palladium-Catalyzed Cross-Coupling Reaction: Structure and Physical Properties. *Chem.–Eur. J*. **21**, 6523–6527 (2015).
4. Yano, Y., Ito, H., Segawa, Y. & Itami, K. Helically Twisted Tetracene: Synthesis, Crystal Structure, and Photophysical Properties of Hexabenzo[*a,c,fg,j,l,op*]tetracene. *Synlett* **27**, 2081-2084 (2016).
5. Moleele, S. S., Michael, J. P. & de Koning, C. B. Methodology for the synthesis of 1,2-disubstituted arylnaphthalenes from α-tetralones. *Tetrahedron* **62**, 2831–2844 (2006).
6. Shi, Z., Zhang, X., Yang, G., Su, Z. & Cui, Z. Synthesis and characterizations of novel spindle-like terphenyl-type chromophores for non-linear optical materials. *Tetrahedron* **67**, 4110–4117 (2011).
7. Braddock, D. C., Cailleau, T., Cansell, G., Hermitage, S. A., Pouwer, R. H., Redmond, J. M. & White, A. J. P. The reaction of aromatic dialdehydes with enantiopure 1,2-diamines: an expeditious route to enantiopure tricyclic amidines. *Tetrahedron: Asymmetry* **21**, 2911–2919 (2010).
8. Xia, C. & Advincula, R. C. Ladder-Type Oligo(*p*-phenylene)s Tethered to a Poly(alkylene) Main Chain: The Orthogonal Approach to Functional Light-Emitting Polymers. *Macromolecules* **34**, 6922–6928 (2001).
9. Bodzioch, A., Owsianik, K., Skalik, J., Kowalska, E., Stasiak, A., Różycka-Sokołowska, E., Marciniak, B. & Bałczewski, P. Efficient Synthesis of Bis(dibromomethyl)arenes as Important Precursors of Synthetically Useful Dialdehydes. *Synthesis* **48**, 3509–3514 (2016).
10. Ma, Y., Zheng, Q., Wang, L., Cai, D., Tang, C., Wang, M., Yin, Z. & Chen, S.-C. Improving the photovoltaic performance of laddertype dithienonaphthalene-containing copolymers through structural isomerization. *J. Mater. Chem. A* **2**, 13905–13915 (2014).
